# Ratiometric Detection of pH‐Induced i‐Motif Folding Based on a Dual Emissive Cytosine Analog

**DOI:** 10.1002/cbic.202500526

**Published:** 2025-10-08

**Authors:** Nicolas P. F. Barthes, Hoang‐Ngoan Le, Benoît Y. Michel, Alain Burger

**Affiliations:** ^1^ Université Côte d’Azur Institut de Chimie de Nice, UMR 7272 CNRS Parc Varose 06108 Nice Cedex 2 France

**Keywords:** cytosine analogs, dual‐emission probes, i‐motif DNAs, pH sensors, ratiometric fluorescence

## Abstract

A dual‐emissive cytosine analog (**TCC**), based on a 2‐thienyl‐3‐hydroxychromone scaffold, is incorporated into oligodeoxynucleotides to monitor the folding state of DNA i‐motif structures. This modified nucleobase exhibits two distinct emission bands (IN* and IT*), each responding differently to microenvironmental changes, enabling ratiometric detection. The photophysical properties of TCC are systematically characterized in various solvents and DNA contexts, including single‐stranded, double‐stranded, and i‐motif‐forming sequences. The IN*/IT* emission ratio and the wavelength of the IT* band act as robust and orthogonal reporters of hydration, base stacking, and protonation states. In fully paired duplexes, the T* band is quenched and blue‐shifted, while i‐motif folding results in both fluorescence enhancement and a redshift of the T* emission. Additionally, the probe distinguishes mismatched base pairs and abasic sites, offering further insights into local structural defects. Overall, this ratiometric nucleobase analog enables real‐time, multiparametric monitoring of i‐motif folding with high sensitivity, and holds promise for extension to other noncanonical DNA structures. The findings further establish the 3‐hydroxychromone platform as a powerful tool for the rational design of fluorescent sensors targeting dynamic nucleic acid architectures.

## Graphical abstract

A dual‐emissive cytosine analog enables ratiometric fluorescence detection of i‐motif DNA folding. This study reveals a sensitive method to monitor pH‐dependent i‐motif conformational changes, providing a valuable tool for investigating DNA secondary structures in vitro.

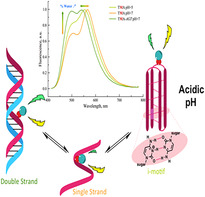

## 1 Introduction

**Figure 1:**
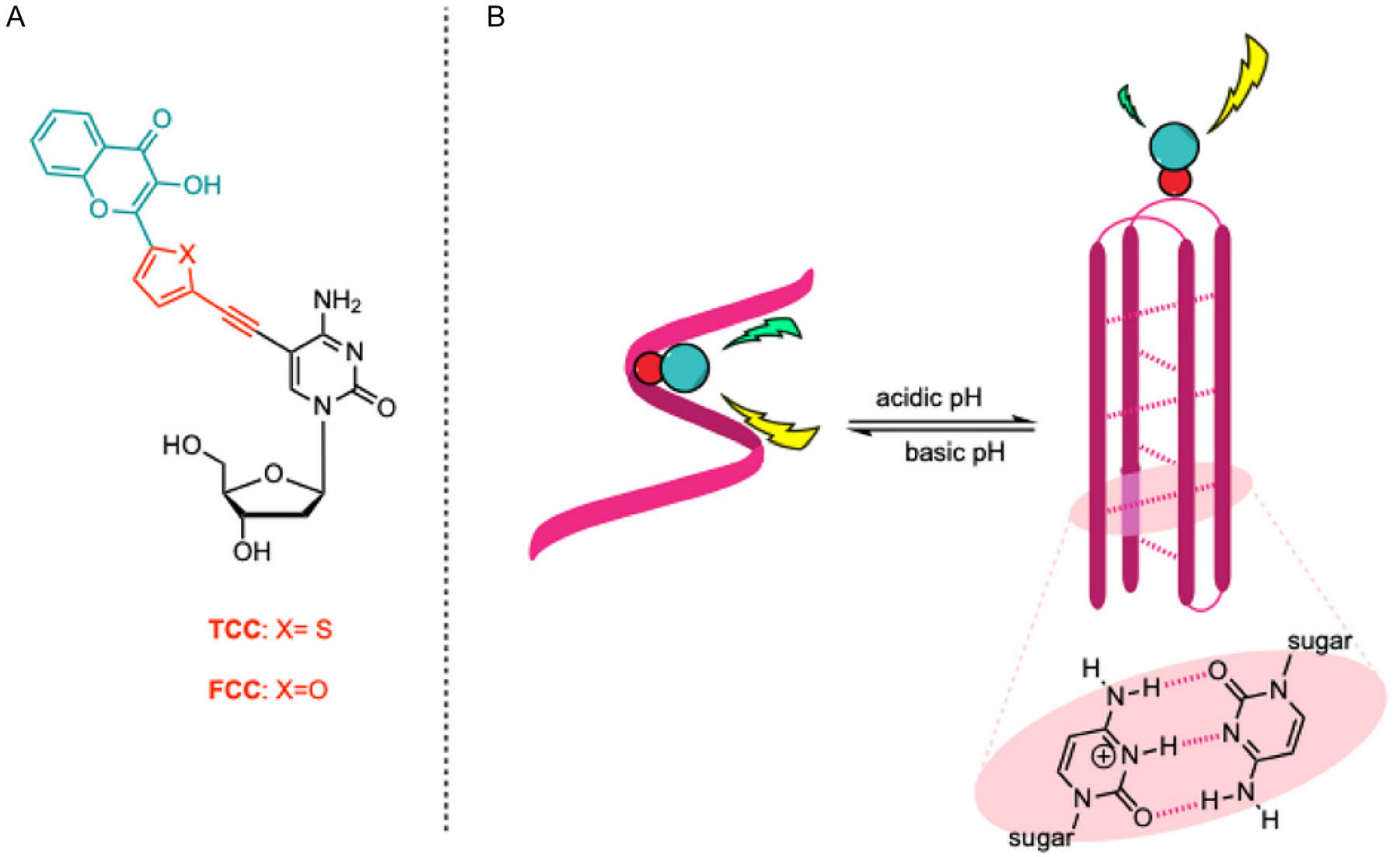
A) Representative concept of the ratiometric detection for the conformational study of acidic pH‐folded i‐motif. B) Structure of the dual emissive 3HC‐cytidine conjugate **TCC** and **FCC**.

**Figure 2:**
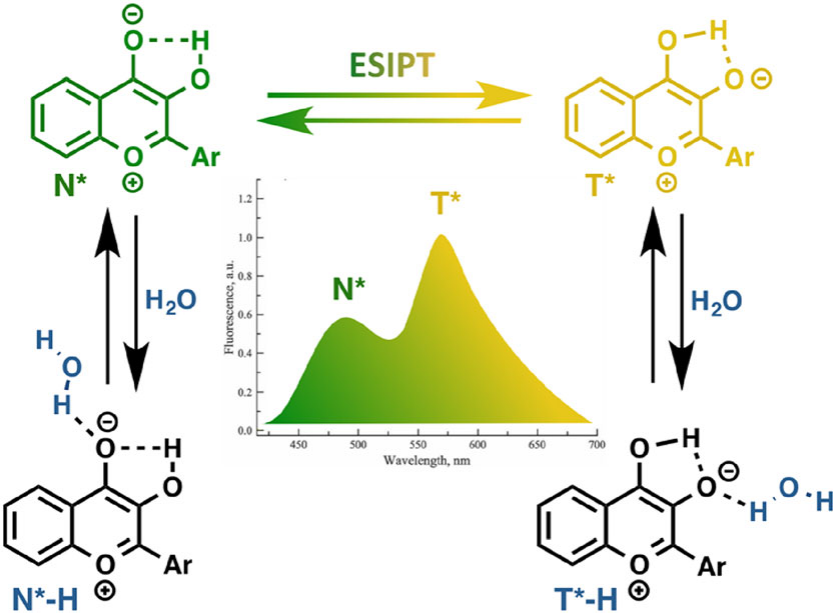
Proposed mechanism for the 3HC‐sensing of local hydration. Excited states N^*^ and N^*^‐H, and T^*^ and T^*^‐H of the normal and tautomer forms are in equilibrium. N^*^‐H undergoes ESIPT reaction on a much slower time scale than N^*^. N^*^ and N^*^‐H, and T^*^ and T^*^‐H generate the higher and lower energy emission bands, respectively, referred as N^*^ and T^*^ on the spectrum for simplification.

Noncanonical nucleic acid structures such as i‐motifs have attracted growing interest for their potential regulatory roles. These four‐stranded DNA architectures, stabilized by hemiprotonated C·C^+^ base pairs under mildly acidic conditions, adopt compact conformations distinct from B‐form duplexes.^[^ ^1^ ^,^ ^2^ ^]^ Monitoring i‐motif folding is essential to uncover their biological relevance.^[^ ^1^ ^–^ ^3^ ^]^ While Förster resonance energy transfer (FRET)‐based assays allow dynamic tracking,^[^ ^4^, ^5^ ^–^ ^6^ ^]^ they require dual labeling and are sensitive to fluorophore distance constraints.^[^ ^7^ ^]^ In contrast, single probes for intensity based sensing^[^ ^8^, ^9^, ^10^, ^11^, ^12^, ^14^ ^–^ ^15^ ^]^ offer simplicity but lack internal referencing, making them vulnerable to variations in dye concentration or instrumental settings. Ratiometric fluorescent probes based on single dyes with dual emission bands address these issues by reporting emission ratios rather than absolute intensities.^[^ ^16^ ^]^ Wavelength shift probes can also be employed for ratiometric measurements; however, this possibility has not been explored in the case of DMAC, a push–pull derivative of cytidine.^[^ ^13^ ^]^ Integrating single probes into DNA without disrupting structure remains a challenge. Here, we present **TCC**, a dual‐emissive cytosine analog bearing a 3‐hydroxychromone (3HC) moieties (**Figure** **1**). This fluorophore displays two emission bands (N* and T*) arising from excited‐state intramolecular proton transfer (ESIPT) (**Figure** **2**) and show strong solvatofluorochromism.^[^ ^17^ ^–^ ^19^ ^]^ Positioned in the loop of an i‐motif‐forming sequence, **TCC** allows nondisruptive, ratiometric detection of pH‐induced folding transitions. The emission ratio reflects local hydration and polarity, offering a sensitive, internally calibrated readout of DNA conformational changes.

## 2 Results and Discussion

### 2.1 Synthesis of the Fluorescent Nucleosides TCC and FCC

**Scheme 1:**
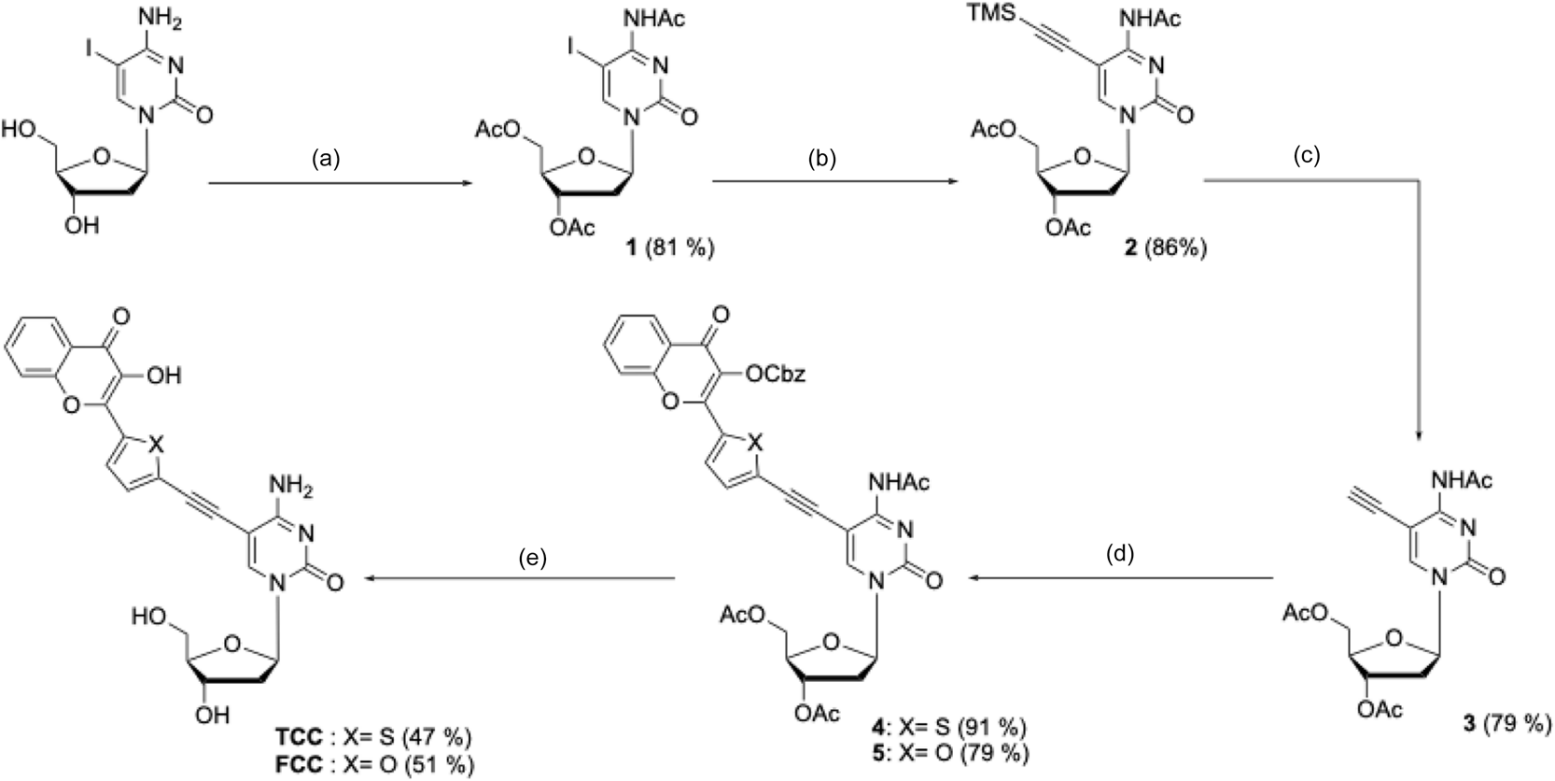
Synthesis of the cytidine analogs bearing 3‐hydroxychromone as a fluorescent reporter: a) DMAP, Ac_2_O, Pyr, 40 °C, 3 h; b) TMS‐acetylene, CuI/PdCl_2_(Ph_3_)_2_ (7 mol%), Et_3_N, THF, 55 °C, 2 h; c) Et_4_NF, THF, rt, 30 min; d) 6 or 7, CuI/PdCl_2_(Ph)_2_ (7 mol%), Et_3_N, THF, 55 °C, 2 h; e) NH_4_OH, MeOH, rt, 3 h.

Dual‐emissive nucleoside analogs **TCC** and **FCC** were synthesized following a retrosynthetic strategy adapted from the deoxyuridine series.^[^ ^20^ ^]^ This approach relies on the Algar–Flynn–Oyamada reaction for the construction of the 3HC core, and Sonogashira couplings for the final assembly (**Scheme** **1**). More specifically, peracetylation of 5‐iodo‐2′‐deoxycytidine was followed by an efficient coupling with TMS‐acetylene, affording the alkynyl intermediate **2**. In previous studies on the deoxyuridine series, N3 protection with a toluoyl group proved essential during fluorophore assembly to prevent side reactions leading to bicyclic byproducts. In the present case, monoacetylation at the N4 position plays a similar protective role, as 5‐endo‐dig cyclization is even more favored in the cytidine series, as previously reported.^[^ ^21^ ^,^ ^22^ ^]^ After mild deprotection of the silyl group using tetraethylammonium fluoride, the resulting ethynyl derivative was coupled via a Sonogashira reaction with the Cbz‐protected 3HC precursor. Final treatment with aqueous ammonia yielded the target emissive nucleosides **TCC** and **FCC** in satisfactory yields suitable for spectroscopic characterization.

### 2.2 Photophysical Studies of TCC and FCC

Photophysics of both derivatives **TCC** and **FCC** were evaluated in different solvents over a wide range of polarity. To this end, five protic (1,1,1,3,3,3‐hexafluoroisopropanol (HFIP), H_2_O, MeOH, EtOH, and BuOH) and six aprotic solvents (acetonitrile, 1,2‐dichloroethane, chloroform, ethyl acetate, tetrahydrofuran, and dioxane) were selected. Their polarity were estimated and ranked by the use of the Reichardt's polarity parameter ET(30) that takes into account both their H‐bond donor ability and dielectric constant.^[^ ^23^ ^]^ The absorption and emission properties of **TCC** and **FCC** are reported in **Table** **1** **.**

**Table 1:** Spectroscopic properties of TCC and FCC in different solvent polarities.

Solvent	*E*_T_(30)a)	*λ*_Abs_ b) [nm]		*λ*_N*_ c) [nm]		*λ*_T*_ d) [nm]		IN*/IT*e)		Φf) [%]		Bg)	[M^−^^1^ cm^−^^1^]
		TCC	FCC	TCC	FCC	TCC	FCC	TCC	FCC	TCC	FCC	TCC	FCC
HFIP	65.3	394	387	512	510	–	–	–	–	0.23	0.37	6270	11,670
H_2_O	63.1	396	389	529	515	–	–	–	–	0.06	0.26	1635	8200
MeOH	55.4	393	385	517	511	569	552	0.65	0.88	0.22	0.24	5995	7570
EtOH	51.9	395	387	511	502	575	563	0.37	0.53	0.20	0.22	5450	6940
BuOH	49.7	399	390	499	491	574	564	0.29	0.39	0.22	0.19	5995	5995
CH_3_CN	45.6	392	381	492	484	581	572	0.29	0.26	0.20	0.17	5450	5365
DCE	41.3	394	384	468	466	579	571	0.11	0.08	0.20	0.22	5450	6940
CHCl_3_	39.1	398	389	467	481	575	565	0.15	0.10	0.26	0.28	7085	8835
EtOAc	38.1	394	383	475	464	588	576	0.20	0.11	0.19	0.20	5180	6310
THF	36.2	398	386	475	464	590	579	0.20	0.11	0.19	0.20	5180	6310
Dioxane	36.0	397	388	467	454	587	576	0.39	0.20	0.18	0.28	4900	8835

a) Reichardt's empirical solvent polarity index;^[^ ^23^ ^]^

b) Position of the absorption maximum;

c) Position of the N* band maximum in nm;

d) Position of the T* band maximum in nm;

e) Ratio of the two intensities at their maxima; ±2.5% mean standard deviation;

f) Quantum yields determined using as references, 3‐hydroxyflavone in toluene (Φ = 0.29),^[^ ^42^ ^]^ 4′‐(dimethylamino)‐3‐hydroxyflavone in ethanol (Φ = 0.27),^[^ ^43^ ^]^ or quinine sulfate in 0.1 M H_2_SO_4_ (Φ = 0.54); ±10% mean standard deviation;^[^ ^44^ ^]^

g) Brightness: B = *ε*.ϕ.

**Figure 3:**
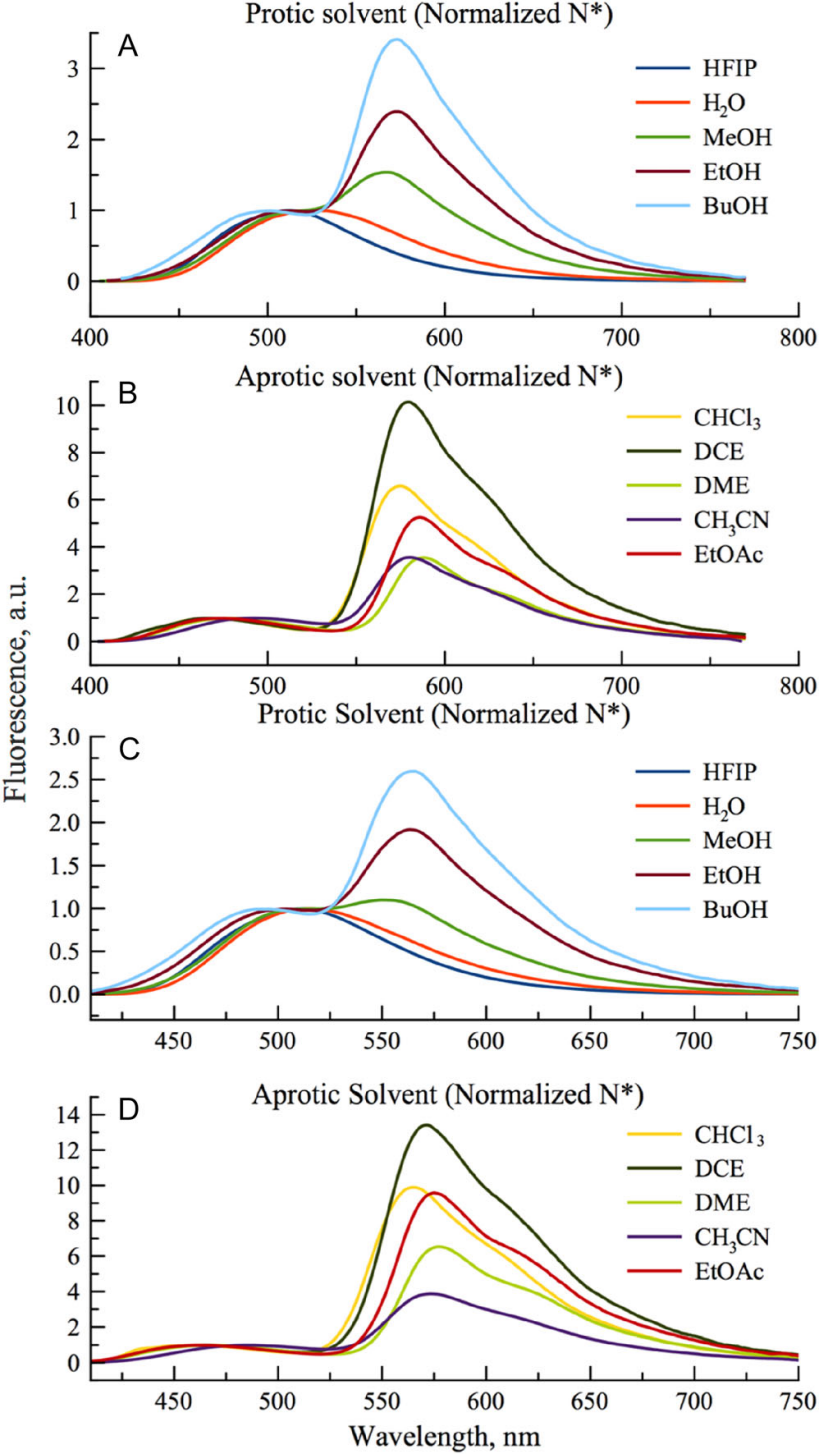
Normalized fluorescence spectra of **TCC** and **FCC** in protic A) and C), respectively, and aprotic solvents B) and D), respectively. All the spectra are normalized by the N^*^ band (excitation wavelength corresponds to the absorbance maximum of each compound in the considered solvent).

**TCC** and **FCC** absorbed intensively (*ε*_**TCC**_ = 27.250 M^−1^ cm^−1^ and *ε*_**FCC**_ = 31.550 M^−1^ cm^−1^ in dioxane) and showed large Stokes shifts, the difference between absorption and emission (N*) maxima (66 to 133 expressed in nm for conveniency). They exhibited a dual emission in all the considered solvents, except for water and HFIP (Table 1, **Figure** **3**, and Figure S1 and S2, Supporting Information). The short emission wavelength (454–529 nm) can be attributed to the N* band and the longer one (552–590 nm) to the T* band. The positive solvatochromism of the N* band is directly related to the intramolecular charge transfer (ICT) occurring between the uracil N‐1 (acting as a donor) and the ketone of 3HC (acceptor).^[^ ^20^ ^,^ ^24^, ^25^ ^–^ ^25^, ^26^ ^]^ The polarity scale of Lippert–Mataga proved informative to estimate such ICT character (Section S2.2, Supporting Information).^[^ ^7^ ^,^ ^27^ ^,^ ^28^ ^]^ Plotting the Stokes shift Δ*ν* as a function of the orientation polarizability Δf correlated linearly with positive slopes for both compounds. This reflects an increase of the dipole moments in the N* states of **TCC** and **FCC** after Franck–Condon light absorption. Interestingly, these transition dipole moments are more important in cytidine (Δμ_**TCC**_ and Δμ_**FCC**_ = 14,2 and 14,9 D) than in uridine series (Δμ_**TCU**_ and Δμ_**FCU**_ = 13 and 14,5 D), previously reported.^[^ ^19^ ^]^ Such differences could be attributed to the stereoelectronic properties of the amidine at C‐4. Significant blueshifts of the T* maxima (20–25 nm) were observed in protic solvents indicating H‐bond formation between the 3‐phenoxide oxygen in the T* state and the donor proton from the solvent (Figure 1).^[^ ^29^ ^,^ ^30^ ^]^

Both fluorescent analogs showed a similar trend in their IN*/IT* ratio, along the increasing polarity and H‐bond donor strength of the solvents. A two‐ to threefold increase of the intensity ratio was observed from MeOH to CH_3_CN, for **TCC** and **FCC**, respectively. It is noteworthy these two probes demonstrated higher sensitivity to H‐bonding than polarity effects since both solvents present similar dielectric constants. Such interpretation is supported by the Lippert–Mataga plots. Indeed, since Stokes shifts in protic solvents were much higher, they appeared out of the linear fit (Figure S7, Supporting Information) suggesting specific interactions between the ratiometric fluorophore and the protic solvent, as already reported for donor–acceptor solvatofluorochromic dyes.^[^ ^7^ ^]^ To sum up, **TCC** and **FCC** appeared as sensitive as reference 3HCs.^[^ ^18^ ^,^ ^31^, ^32^ ^–^ ^33^ ^]^

The quantum yields ranged from 18% (B = 4.900 M^−1^ cm^−1^) to 37% (B = 11.670 M^−1^ cm^−1^), except for **TCC** in water (6%, B = 1635M^−1^ cm^−1^), consistent with the behavior commonly observed for push–pull dyes.^[^ ^34^ ^]^ Notably, both dyes exhibited their highest quantum yields in HFIP, a solvent more polar than water, whose strong acidity completely suppressed the ESIPT process, leading to a single N* emission band. Therefore, the reduced quantum yield in water is unlikely to be attributed solely to hydrogen‐bond strength. A more plausible explanation for the fluorescence quenching may involve the concerted action of multiple water molecules trapping electrons from the excited‐state species.^[^ ^35^ ^]^ Interestingly, **FCC** presents an attractive quantum yield in water as previously observed for the dU series.^[^ ^20^ ^]^

**Figure 4:**
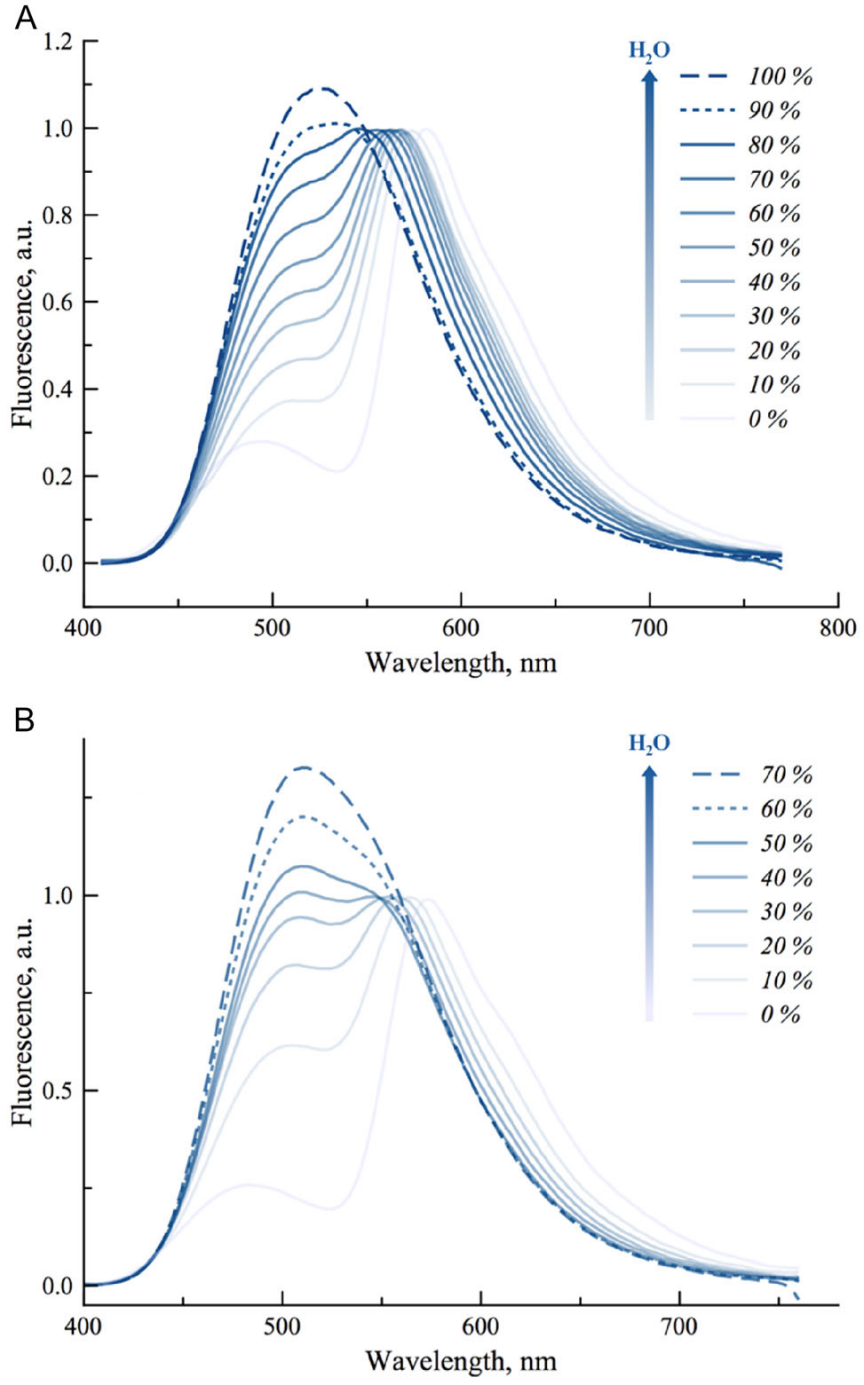
Sensitivity of the emission spectra to hydration for A) **TCC** and B) **FCC**. Data were recorded in gradual mixtures of acetonitrile and water and normalized by T^*^ band.

Since the ultimate goal of these emissive nucleosides is their incorporation into the hydrated major groove of nucleic acids, additional studies were conducted in acetonitrile–water and dioxane–water mixtures to further investigate the hydration sensitivity of compounds **TCC** and **FCC** (**Figure** **4**). By comparing spectra in HFIP vs. H_2_O, the slight shoulder suggests that ESIPT reaction is not totally disrupted in bulk water. Regardless of the organic cosolvent used, a ratiometric fluorescence response was maintained up to 80% and 60% water content for **TCC** and **FCC**, respectively. For instance in acetonitrile, the gradual addition of water led to an increase in the IN*/IT* ratio (from 0.28 to 0.95 for **TCC**, and from 0.26 to 1.1 for **FCC**), along with a blueshift of the T* band by 52 nm and 59 nm, respectively. Plotting the IN*/IT* ratio as a function of water content yielded a linear correlation. Similar results were observed in dioxane/water mixtures, supporting the idea that these ratiometric variations arise primarily from hydration effects (Supporting Information, Section S2.2, Supporting Information). Comparison of the slopes revealed that **FCC** exhibits greater sensitivity to hydration than **TCC**. Although **FCC** showed a marked change (42%) upon addition of the first 10% of water, its dual emission became barely detectable above 50%, which may limit its applicability in highly hydrated environments such as nucleic acids for sensing conformational changes.^[^ ^19^ ^]^ Nevertheless, **FCC** appears better suited for detecting hydration in low‐water environments, such as biological membranes or nucleic acids engaged in protein interactions.

Notwithstanding its moderate quantum yield in bulk water, **TCC** remains significantly brighter than most solvatofluorochromic dyes.^[^ ^34^ ^]^ **FCC** was excluded from incorporation into DNA strands and further investigations due to its poor fluorescence in aqueous environments (see Figure 4), whereas **TCC** retained sufficient emission for reliable ratiometric measurements. With its pronounced sensitivity to hydration and broader dynamic range, **TCC** emerges as a promising fluorescent probe for detecting DNA conformational changes driven by subtle variations in local hydration.

### 2.3 Preparation of the Phosphoramidite M and Incorporation into ODNs

Owing to its attractive photophysical properties (*vide* *supra*), **TCC** was selected for incorporation into ODN via a phosphoramidite strategy as described in Supporting Information (Sections S1.2 and 3.1, Supporting Information). High pressure liquid chromatography, Ultra Violet (UV)/visible and mass spectrometry analysis ascertained the purity and integrity of the synthesized ODNs (Table S2 and Figure S3–S7, Supporting Information).

### 2.4 Photophysical Characterization of Labeled 15‐Mer ODNs

Pentadecamer model sequences d(CGT TTT XMX TTT TGC), where X = T, A, C and G, and M = conjugate **TCC**, referred as TMT, AMA, CMC, and GMG, were selected to investigate the effects on the spectroscopic properties of the bases flanking **TCC**. The dye was located at the center of the DNA sequences in order to avoid structural disorders occurring at the terminal parts of the duplex. Control studies were conducted with the corresponding wild‐type 15‐mers d(CGT TTT XCX TTT TGC) where a C substitutes the fluorescent reporter. The resulting duplexes were then annealed mixing each XMX or XCX single strands with its complementary sequence (denoted in italics) presenting a natural base or an abasic site (Ab) at the position opposite to **TCC** (**Table** **2** and Table S4, Supporting Information). Thereby, complementary strands were d(*GCA AAA YXY AAA ACG*), where Y = *A, T, C* or *G*, and *X* corresponds to *G* (matched pair), *T, C, A* (mismatched cases) or an abasic site.

**Table 2:** Melting temperatures and photophysical properties of labeled single strand and duplexes.

Duplexesa)	*T*_m_ b) [°C]		*λ*_abs_ c) [nm]	*λ*_N*_ d) [nm]	*λ*_T*_ e) [nm]	IN*/IT*c)	Φg) [%]
	TCC	WT					
**TMT**	–	–	401	506	569	0.61	18
**TMT**‐*AGA*	45.1	49.7 [51.5]	402	499	548	0.97	17
**AMA**	–	–	408	505	569	0.63	19
**AMA**‐*TGT*	45.0	50.3 [50.9]	398	501	541	1.03	13
**CMC**	–	–	404	509	569	0.64	12
**CMC**‐*GGG*	50.0	54.2 [56.6]	403	508	547	0.87	6
**GMG**	–	–	403	506	571	0.50	2
**GMG**‐*CGC*	52.8	59.1 [58.3]	397	505^h)^	545	0.7	<1

a) 2 µM concentration of ODN in pH 7.0 buffer (20 mM sodium phosphate, 150 mM NaCl, 1 mM EDTA);

b) Tm of the corresponding duplex formed from unmodified duplexes (melting temperatures: ±0.5 °C) and its theoretical values given in square brackets;

c−g) See Table 1.

**TCC** was expected to base‐pair preferentially *G*. Temperature‐induced melting experiments confirmed that the most stable duplexes were the fully complementary sequences (M‐*G*). (Tables 2 and S4, Figure S14 and S15, Supporting Information). Thermal denaturation studies revealed that both labeled matched and mismatched duplexes generally exhibited a reduced stability compared to the corresponding wild‐type duplexes (ΔTm = −0.2 to −6.3 °C), with the extent of destabilization ranging from negligible to moderate depending on the sequence context. Notably, an exception was observed for C opposite **TCC**, which resulted in a stabilizing effect (ΔTm = +1.9 to +3.5 °C, Table 2). As reported in dU series,^[^ ^19^ ^]^ stabilization of labelled duplexes in mismatched case with C may originate from the significant π‐stacking of the accommodated **TCC** with the surrounding bases as well as the three H‐bonds of the extra‐helical cytosine, formed with water coating. Consistent with earlier findings on dU analogs,^[^ ^19^ ^]^ labeled duplexes containing an abasic site opposite to 3HC‐conjugated nucleobase displayed significantly higher stability than the corresponding controls (ΔTm = +4.7 to +7.6 °C). Such increase in Tm would suggest that the fluorescent cytosine accommodates well the abasic lesion by means of its large stacking area and its hydrophobic character.^[^ ^36^ ^]^ Further circular dichroism experiments showed that the probe did not affect the B‐helix morphology of the labeled DNA duplexes (Figure S16, Supporting Information).

**Figure 5:**
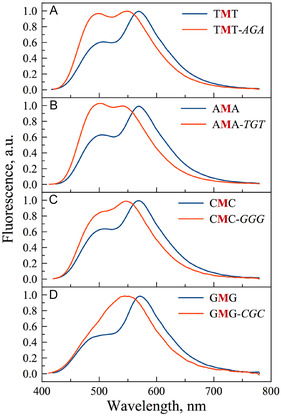
Fluorescence spectra of labeled ss and matched duplexes. A) TMT and TMT‐AGA, B) AMA, AMA‐TGT, C) CMC, CMC‐GGG, and D) GMG, GMG‐CGC recorded in pH 7.0 buffer (20 mM cacodylate, 150 mM NaCl, 1 mM EDTA) and normalized by the T^*^ band.

UV–visible and fluorescence spectra of single‐ and double‐stranded DNA incorporating **TCC** were subsequently recorded in pH 7.0 cacodylate buffer (section S4.1, Supporting Information). Compared to their matched double‐stranded counterparts, the labeled single strands exhibited a red‐shifted visible absorption (397–403 nm vs. 401–408 nm). This redshift in the single‐stranded context may be attributed to more efficient π‐stacking of the hydrophobic fluorophore with neighboring bases. Redshifts arising from π‐stacking interactions are commonly observed for a variety of dyes incorporated into nucleic acids. Fluorescence spectra revealed two well‐resolved emission bands for nearly all strand combinations (**Figure** **5** and S23, Supporting Information). Among these, the fully matched duplexes displayed the highest IN*/IT* ratios and the most pronounced blueshifts of the T* band. Specifically, single‐stranded sequences exhibited IN*/IT* values in the range of 0.50–0.63 with the T* band centered around 570 nm, whereas the fully complementary duplexes showed values of ≈0.87–1.03 and a blue‐shifted T* maximum at 545 nm.

These dual readouts—intensity ratio and emission wavelength—thus enable straightforward discrimination between single strands and fully matched duplexes. Moreover, the IN*/IT* ratio also confirmed that A*T* base pairs are more hydrated than G*C* pairs (≈1.0 vs. ≈0.85). The blueshifts in both absorption and T* emission, along with the marked increase in the N* band intensity, strongly support the positioning of the dye within the hydrated major groove. In addition, a 1.5‐ to 2‐fold decrease in quantum yield upon hybridization with fully complementary strands further confirms the localization of the fluorophore in a more hydrated environment. Taken together, our data closely mirror those obtained with the dU series, reinforcing the generalizability of this design strategy for emissive nucleosides.^[^ ^19^ ^]^

To further evaluate the discriminatory power of the dual‐emissive cytosine analog **TCC**, we investigated the spectroscopic behavior of mismatched duplexes bearing the **TCC**‐modified base opposite a noncanonical partner (*T, A*, or *C*) or an abasic site. These mismatches are relevant not only as models of point mutations but also to probe the microenvironmental sensitivity of the ratiometric reporter. Compared to the fully matched duplexes, mismatched constructs as well as duplexes containing abasic sites opposite the probe exhibited markedly altered photophysical properties (Table S4 and Figure S23, Supporting Information). In particular, fluorescence measurements revealed a reduction in IN*/IT* ratio, typically ≈0.55–0.75, consistent with a more hydrophobic and less hydrated local environment around the probe. Notably, the T* band showed a redshift (≈10–27 nm) compared to matched counterparts, reinforcing the idea that the environment is less structured and less solvent‐exposed likely due to increased stacking interactions. This behavior echoes previous observations for 3HC‐modified uridines, where stacking with flanking bases partially compensates for the absence of base‐pairing, and hydration remains limited due to the local cavity.^[^ ^19^ ^]^ The matched duplexes of CMC and both GMG single and double strands exhibited the lowest quantum yields (<6%). These low quantum yields can easily be explained by a direct contact quenching of **TCC** by G, a well‐known fluorescence quencher.^[^ ^16^ ^,^ ^37^ ^]^ By contrast, TMT and AMA single strands and duplexes revealed the highest sensitivity to discriminate the conformational changes (IN*/IT* variation), the most important quantum yields (13–34%) and the best resolution between the two emission bands (Figure S23, Supporting Information). For all these reasons, A and T appeared as the most suitable flanking bases for **TCC** in order to obtain an informative site‐specific response during further applications. Overall, the dual‐emission system enables clear discrimination between matched and mismatched duplexes through both ratiometric and spectral criteria. These results confirm that the probe is not only sensitive to hydration changes but also capable of reporting subtle local structural perturbations—highlighting its potential utility for mutation detection assays or mapping non‐Watson–Crick pairing events.

As the photophysical studies rely on the ESIPT process—an acid–base reaction—it was essential to confirm the stability of the fluorophore across the pH range relevant to i‐motif folding. To this end, the effect of pH was first evaluated by UV spectroscopy using the model AMA and TMT 15‐mer sequences in various buffers spanning pH 2.2–10.8 (Table S3, Supporting Information). No significant pH‐dependent changes were observed in UV, fluorescence, or CD spectra between pH 2.2 and 8.2 (Figure S21 and S22, Supporting Information). However, above pH 8.2, the anionic form of the 3HC fluorophore began to emerge, as evidenced by a characteristic redshift in the absorption band (Δ*λ* ≈ 60 nm), reaching a maximum at 470 nm. Importantly, no conformational changes were detected in the CD spectra across the entire pH range (Figure S18, Supporting Information). The apparent pKa of the 3HC moiety within model ODNs was determined to be ≈8.9 (Figure S22, Supporting Information). This value is well suited for monitoring i‐motif folding, which typically occurs around pH 5.9–6.5.

### 2.5 Spectroscopic Studies of Labeled i‐Motifs

This labeling strategy, based on **TCC**, was then applied to monitor the single‐stranded to i‐motif transition. The position of **TCC** within the i‐motif was carefully chosen to preserve the native folding and stability of the structure. Incorporation into the C:C^+^ core significantly disrupted i‐motif formation (data not shown), likely due to protonation changes or steric hindrance from the extended aromatic system. As a result, **TCC** was introduced in a loop region, minimizing perturbation while allowing ratiometric sensing. A 21‐mer telomeric sequence was used, and the dual‐emissive probe (**TCC**= M) was inserted into the central loop, yielding the sequence 5′‐d(CCCTAACCCTMACCCTAACCC)‐3′, hereafter referred to as i‐TMA. Thermal denaturation and circular dichroism studies confirmed that the fluorescent label did not destabilize or perturb either the B‐form duplex or the i‐motif structure (**Table** **3**, Figure S15, S16 & S17, Supporting Information). The CD spectra showed the characteristic positive peaks at ≈290 nm for i‐motif in comparison to the ≈275 and≈265 nm for the unfolded single strand and B‐form duplex, respectively (Figure S17, Supporting Information).^[^ ^38^ ^]^ CD measurements performed under variable pH conditions of both wild‐type and labeled samples revealed a distinct conformational transition from the unfolded to the i‐motif structure, with a midpoint pH (pH_m_) of 6.1 ± 0.1 (Figure S18, Supporting Information). Steady‐state fluorescence measurements enabled the monitoring of conformational transitions, either through single‐wavelength emission detection or by calculating the IN*/IT* ratios. Results consistent with CD observations were obtained by tracking the fluorescence emission of TMA as a function of pH, demonstrating that **TCC** can act as a fluorescent reporter for sensing this conformational change (Figure S24, Supporting Information). As an additional control, the reversibility of i‐motif folding was confirmed by repeated additions of 5 M HCl and NaOH aliquots to alternately adjust the pH between 5.3 and 7.2 (Figure S25, Supporting Information).

**Table 3:** Spectroscopic data for labeled i‐TMA in single and double stand forms in various pH conditions.

pH	Sequencesa)	*T*_m_ b) [°C]		*λ*_N*_ c) [nm]	*λ*_T*_ d) [nm]	IN*/IT*e)	Φf) [%]
		TCC	WT				
5.3	**TMA**	50.7	50.5	507	557	0.83	22
7.2	**TMA**	–	–	506	566	0.70	35
	**TMA**‐*AGT*	63.0	62.7	500	543	0.99	12

a–f) See Table 1.

**Figure 6:**
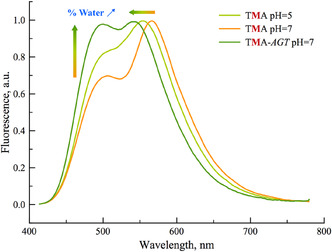
Ratiometric fingerprinting: normalized fluorescence spectra of the folded (pH = 5) and unfolded (pH = 7) i‐TMA, as well as its corresponding mached duplex i‐TMA–AGT.

**Figure 7:**
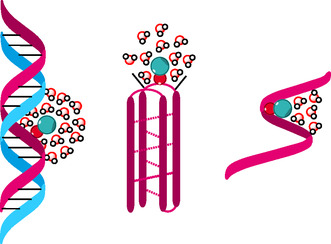
Hydrated environment of **TCC** represented in different morphologies (matched duplex, folded i‐motif, and unfolded single strand).

From steady‐state fluorescence measurements, **TCC** allows clear discrimination between single‐stranded, duplex, and i‐motif conformations, as illustrated in **Figure** **6**. Notably the IN*/IT* ratio shifts from ≈0.7 in the unfolded single‐stranded form to ≈0.8 in the folded i‐motif, while the duplex exhibits a larger value (≈1.0), enabling unambiguous structural assignment. As expected, the matched duplex TMA–*AGT* exhibited the highest IN*/IT* ratio, the most pronounced blueshifts in both the absorption and T* emission bands, and the lowest quantum yield. These observations support the localization of the fluorophore within the hydrated major groove, consistent with TCC–*G* base pairing and an environment comparable to 90% water content (**Figure** **7**). In contrast, the unfolded TMA sequence in Na_2_HPO_4_/citric acid buffer at pH 7.2 displayed the lowest IN*/IT* ratio, a pronounced redshift of the T* band, and a threefold increase in quantum yield (Figure 7). These results indicate that the dye is positioned in a significantly less hydrated environment, equivalent to ≈50% water content. Comparable hydration levels for single‐ and double‐stranded contexts were previously reported in the 3HC‐dU series.^[^ ^19^ ^]^ Remarkably, analysis of the folded TMA i‐motif at pH 5.3 revealed an intermediate IN*/IT* ratio, between those observed for the single strand and the duplex at pH 7.2 (see % variation and Figure 7). Taken together, these findings indicate that the probe, anchored in the central loop of the i‐motif, is oriented toward a moderately hydrated environment corresponding to ≈65% water content. Compared to the matched duplex, the accessibility of the fluorophore to surrounding water molecules is partially reduced due to a shielding effect from flanking bases—though this effect is less pronounced than in the unfolded single‐stranded case (Figure 7). This difference may stem, in the case of the i‐motif, from limited loop flexibility, which likely prevents the π‐stacking from reaching the extent observed in the single‐stranded context. Recent advancements include ^DMA^C, a wavelength shifting cytidine push–pull analog revealing slow exchange of duplex and i‐motif structures,^[^ ^13^ ^]^ BT Cy 1, a supramolecular cyanine probe enabling multichannel discrimination of i‐motifs, G4s, and duplex DNA in serum at nanomolar sensitivity;^[^ ^39^ ^]^ IMCC 6, a coumarin–carbazole dye capable of visualizing i motifs in live cells and zebrafish nucleoli;^[^ ^40^ ^]^ and light up probes such as quinaldine red for selective detection of promoter i‐motifs.^[^ ^41^ ^]^ In contrast to these systems, **TCC** offers the unique combination of site‐specific integration, dual‐band ratiometric response, and fine sensitivity to the microenvironment, enabling quantitative monitoring of conformal transitions as a function of local hydration.

## 3 Conclusion

In this work, we developed and characterized a dual‐emissive cytosine analog incorporating a 3‐hydroxychromone (**TCC**). This bright fluorescent reporter exhibits large Stokes shifts for easier detection and efficiently senses local hydration. This cytidine analog complements the existing set of U and A nucleobases functionalized with chromone fluorophores, thereby expanding the fluorescent genetic alphabet. The two‐color probe **TCC** was successfully incorporated into model DNA oligonucleotides, and its multiparametric fluorescence properties—combining ratiometric response and emission wavelength shift—proved sensitive enough to distinguish perfectly matched duplexes from single strands or mismatched sequences, including abasic sites. This covalent labeling strategy was then applied to monitor pH‐induced folding of i‐motif structures, leveraging both emission channels. A clear and reversible color change (from green to yellow) enabled reliable differentiation between matched duplexes and folded or unfolded i‐motifs. Future work will include comparative studies with our previously reported dU analogs as well as investigations across a broader set of i‐motif sequences, to further explore the generalizability and sequence‐dependence of the ratiometric response. By sensing hydration changes in the vicinity of the probe, this approach eliminates the need for tedious double‐labeling typically required in FRET‐based assays. This opens promising avenues for designing two‐color fluorescent assays to screen for new i‐motif‐targeting molecules, with potential applications in therapeutic development. This work lays the foundation for future *in* *cellula* applications of ratiometric probes for monitoring i‐motif folding. Its ability to distinguish between DNA conformations could prove valuable for imaging dynamic structural transitions, detecting sequence‐dependent polymorphisms, or probing protein–DNA interactions in complex biological environments.

## Supporting Information

Electronic Supplementary Information (ESI) available: Electronic supporting material includes additional figures, full experimental part with synthesis and characterization of all compounds, biochemical procedures, spectroscopic measurements, image analysis, computational methods and copies of NMR spectra. The authors have cited additional references within the Supporting Information.^[45–48]^

## Conflict of Interest

The authors declare no conflict of interest.

## Supporting information

Supplementary Material

## Data Availability

The data that support the findings of this study are available in the supplementary material of this article.
