# Supplementary material for: Ratiometric Detection of pH‐Induced i‐Motif Folding Based on a Dual Emissive Cytosine Analog
Source: Chembiochem. 2025 Oct 8;26(20):e202500526. doi: 10.1002/cbic.202500526 (PMC12582153; doi:10.1002/cbic.202500526)
Supplement: Supplementary file 1 — Supplementary Material [file CBIC-26-e202500526-s001.pdf]

Supporting Information To:

Ratiometric detection of pH-induced i-motif folding based on a dual emissive cytosine analogue

Nicolas P. F. Barthes,[a] Hoang-Ngoan Le,[a] Benoît Y. Michel,*[a] Alain Burger,*[a]

a) Institut de Chimie de Nice, UMR 7272, Université Côte d'Azur, CNRS, Parc Valrose, 06108 Nice Cedex 2, France.

Corresponding authors: alain.burger@univ-cotedazur.fr; benoit.michel@univ-cotedazur.fr

Graphical Abstract:

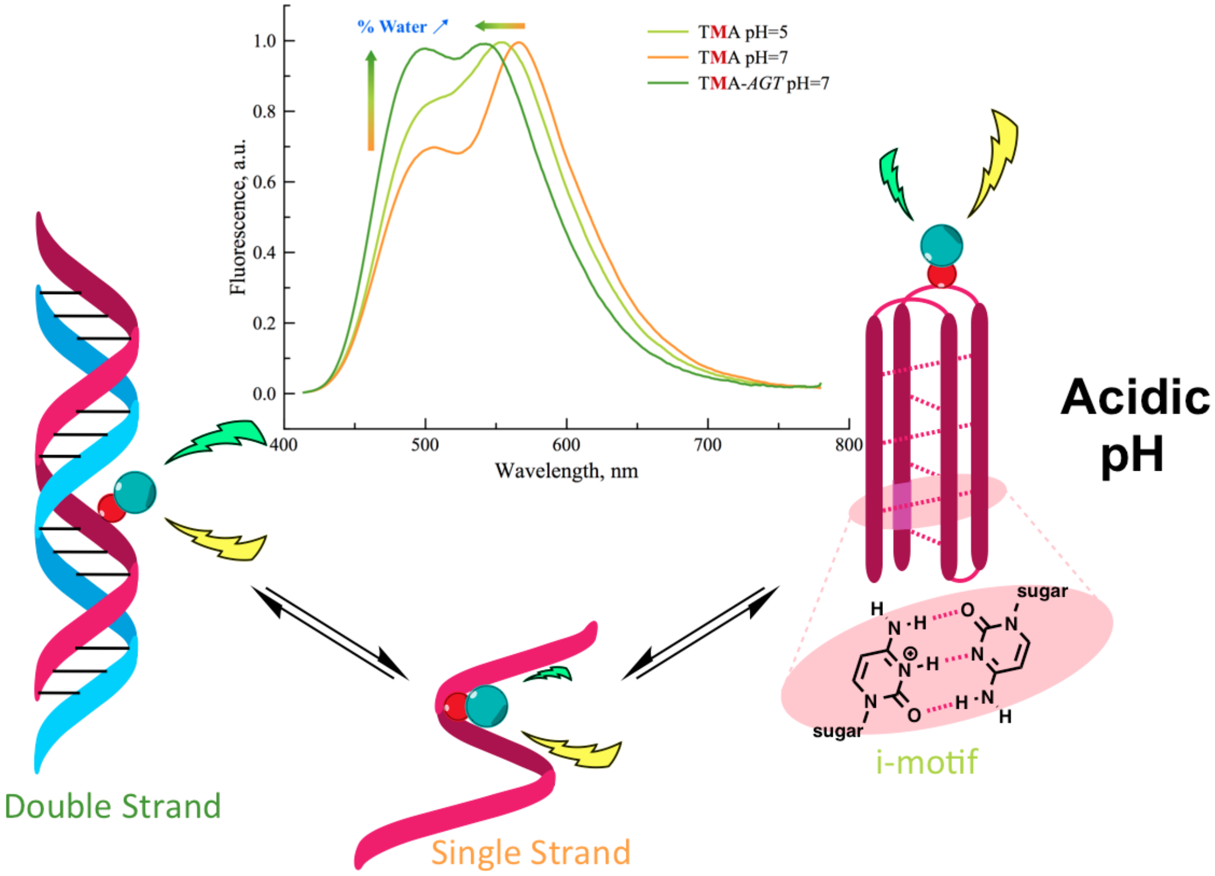

Table of contents

Experimental Section ***Pages***

General Methods...3

Supporting Information

1. Synthetic Procedures...4

1.1 Preparation of the 3-HC fluorophores 6 & 7...................................4

1.2 Preparation of the dual-emissive nucleosides TCC & FCC………4

1.3 Preparation of the emissive amidite 20...8

2. Spectroscopic studies of dual-emissive nucleosides TCC & FCC…...............8

2.1 Purification of the free nucleosides TCC & FCC...........................8

2.2 Steady-state fluorescent measurements..9

2.3 Hydration studies on the emissive nucleosides TCC & FCC..........10

3. ODN synthesis, purification & mass characterizations................................11

3.1 ODN synthesis and purification..12

3.2 MALDI-TOF/TOF analysis of ODNs...13

4. Spectroscopic characterizations of wild-type and labeled ODNs................14

4.1 Preparation of the samples and buffers..14

4.2 Denaturation studies and melting temperatures............................15

4.3 Circular dichroism...17

4.4 Absorbance and Fluorescence spectra..19

5. NMR spectra..24

Experimental section

General methods:

All reactions involving water-sensitive reagents were performed in oven-dried glassware under argon using dry solvents. The synthetic intermediates were beforehand co-evaporated twice with toluene and dried in *vacuo* before use. All chemical reagents were obtained from commercial sources (Sigma-Aldrich, Acros, Alfa Aesar) and were used as supplied. Anhydrous solvents were obtained according to standard procedures.[1] The reactions were monitored by thin-layer chromatography (TLC, Merck silica gel 60 F254 plates) and visualized both by UV radiation (254 & 365 nm) and by spraying with vanillin in ethanol containing H_2SO_4 followed by a subsequent warming with a heat gun. Column chromatography[2] was performed with flash silica gel (40–63 mm). All NMR spectra (^{1}H, ^{13}C, ^{2}D) were recorded on 200 or 500 Bruker Advance Spectrometers (200 or 500 MHz). ^{1}H NMR (200 and 500 MHz), ^{13}C NMR (50 and 125 MHz, recorded with complete proton decoupling), ^{31}P NMR (80 MHz, proton decoupling) spectra were obtained with samples dissolved in $CDCl_3$, CD_2Cl_2, CD_3OD, DMSO-d^6, acetone-*d*6, or CD_3CN with the residual solvent signals used as internal references: 7.26 ppm for C*H*Cl$_3$, 5.32 ppm for CD*H*Cl$_2$, 3.31 ppm for CD$_2$*H*OD, 2.50 ppm for (CD$_3$)(CD$_2$*H*)S(O), 2.05 ppm for (CD$_3$)(CD$_2$*H*)C(O), 1.94 ppm for CD$_2$*H*CN for ^{1}H NMR experiments, and 77.0 ppm for $CDCl_3$, 53.8 ppm for CD_2Cl_2, 49.0 ppm for CD_3OD, 39.4 ppm for $(CD_3)_2S(O)$, 30.8 ppm for $(CD_3)_2C(O)$ for ^{13}C NMR experiments.[3] Chemical shifts (δ) are given in ppm to the nearest 0.01 (^{1}H) or 0.1 ppm (^{13}C). The coupling constants (J) are given in Hertz (Hz). The signals are reported as follows: (s=singlet, d=doublet, t=triplet, m=multiplet, br=broad). Assignments of ^{1}H and ^{13}C NMR signals were achieved with the help of D/H exchange, COSY, DEPT, APT, HMQC, HSQC, HMBC experiments. Regular mass spectra (MS) were recorded on an Esquire 3000 Plus apparatus with ESI in both positive and negative mode. High-resolution mass spectrometry was conducted with a FINIGAN MAT 95 spectrometer with EI or ESI ionization techniques. Supplementary data associated with this article, including the experimental protocols for the synthesis of intermediates **1-20**, the ^{1}H, ^{13}C, ^{31}P, ^{1}H–^{1}H COSY, ^{1}H–^{13}C HMQC, ^{1}H–^{13}C HSQC and ^{1}H–^{13}C HMBC NMR spectra of all compounds, can be consulted in this *Supporting Information*. Systematic flavone and nucleobase nomenclatures are used below for the assignments of each spectrum. All solvents for absorption and fluorescence experiments were of spectroscopic grade. Absorption spectra were recorded on a Cary 300 spectrophotometer (Varian) using 1cm quartz cells. Stock solution of the TCC model **1** was prepared using dioxane. The samples used for spectroscopic measurements contained ≈ 0.1% v/v of the stock solvent. Fluorescence spectra were recorded on FluoroMax 4.0 spectrofluorometer (Jobin Yvon, Horiba). Excitation wavelength was used as mentioned in the corresponding experiments. Photostability studies were conducted in a 100 µl fluorescence cell, excitation and emission slits were set to 4 nm. The concentrations of the samples were 2 µM.

---

1 W. L. F. Armarego and C. L. L. Chai, *Purification of Laboratory Chemicals*, 7th ed.; Butterworth-Heinemann: Oxford, 2012, p. 1024.
2 W. C. Still, M. Kahn and A. Mitra, *J. Org. Chem.* **1978**, *43*, 2923–2925.
3 *a*) H. E. Gottlieb, V. Kotlyar and A. Nudelman, *J. Org. Chem.*, 1997, **62**, 7512–7515; (*b*) G. R. Fulmer, A. Miller, N. H. Sherden and H. E. Gottlieb, *Organometallics*, 2010, **29**, 2176–2179.

1. Synthetic Procedures

1.1 Preparation of the 3-HC fluorophores 6 & 7

The bromothienyl- and bromofuryl-chromones (**6** and **7**) required for Sonogashira coupling (*vide infra*) were prepared as reported (Scheme S1).[4]

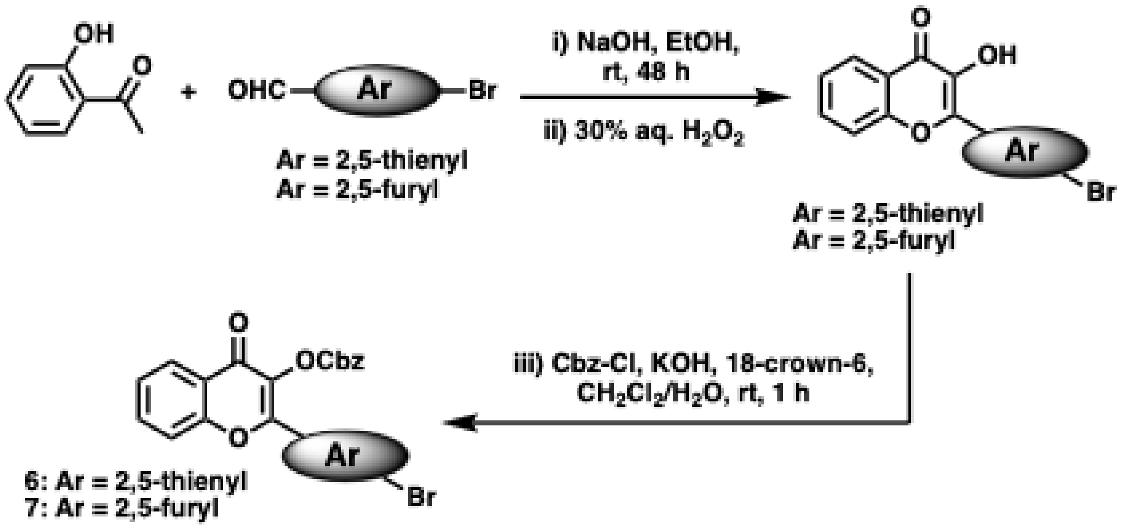

Scheme S1. Synthesis of bromothienyl- and bromofuryl-chromones **6** and **7**.

1.2 Preparation of the dual-emissive nucleosides TCC & FCC

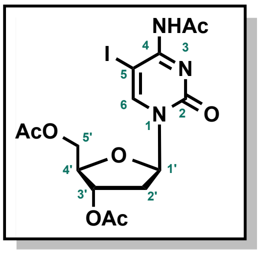

3′,5′-Di-*O*-acetyl-4-*N*-acetyl-5-iodo-2′-deoxycytidine (1): To a stirred solution of 5-Iodo-2'-deoxycytidine (1.27 mmol, 500 mg) in pyridine (10 mL) warmed at 40 °C was added Ac_2O (670 µL, 6.37 mmol, 5 eq.). The reaction mixture was stirred for 3 h at this temperature and then cooled down to rt. The mixture was dissolved in EtOAc and extracted with water (3 x). The combined organic layers were washed with $NaHCO_3$, brine, dried over $MgSO_4$, filtered and reduced under vacuum. The residue was purified by flash chromatography on silica gel eluted with Toluene/Acetone (1:14 → 1:2.5, v/v) to provide the desired product **1** as a white solid (494 g, 1.03 mmol, 81 %). $C_{15}H_{18}IN_3O_7$ (479.23). R_f = 0.53 (Toluene/Acetone = 1:1); ^{1}H-NMR ($CDCl_3$, 200 MHz): δ = 8.07 (1H, s, H6), 6.08 (1H, dd, ^3J=6.4 Hz, ^3J=6.2 Hz, H1'), 5.09 (1H, m, H3'), 4.24 (3H, m, H4', H5'), 2.58–2.65 (1 H, dd, ^2J=13.2 Hz, ^3J=2.6 Hz, H2'$_A$), 2.45 (3H, s, *CH₃*-C(O)-NH), 2.09 (1H, m, H2'$_B$), 2.02 (3H, s, OAc), 1.98 (3H, s, OAc); ^{13}C-NMR ($CDCl_3$, 50 MHz): δ = 170.2, 170.0, 147.9, 87.3, 83.1, 73.9, 63.5, 38.9, 26.2, 21.0, 20.8; MS (ESI$^+$, MeOH) *m/z*: 480.2 $[M+H]^+$, 502.1 $[M+Na]^+$, 519.1 $[M+K]^+$

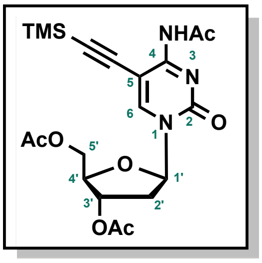

3′,5′-Di-*O*-acetyl-4-*N*-acetyl-5-trimethylsilylethynyl-2′-deoxycytidine (2): To a stirred solution of **1** (2.09 mmol, 1.00 g, previously azeotropically coevaporated with dry pyridine) in THF (21 mL) under argon, NEt_3 (5 eq, 10.43 mmol, 1.5 mL), TMS-acetylene (1.3 eq., 2.71 mmol, 404 µL), $PdCl_2(PPh_3)_2$ (7 mol %, 0.15 mmol, 104 mg), and CuI (7 mol %, 0.15 mmol, 28 mg) were sequentially added. The reaction mixture was refluxed for 2 h then cooled down to rt, filtered through a Celite® 545 pad and the volatiles were evaporated in vacuo. The residue was purified by flash chromatography on silica gel eluted with Toluene/Acetone (1:11 → 1:5, v/v) to provide the desired compound **2** as a beige solid (674 mg, 1.79 mmol, 86 %). $C_{20}H_{27}N_3O_7Si$ (449.54). R_f = 0.65 (Toluene/Acetone = 1:1). ^{1}H-NMR ($CDCl_3$, 200 MHz): δ = 8.11 (1H, s, H6), 6.23 (1H, dd, ^3J=6.6 Hz, ^3J=6.2 Hz, H1'), 5.18–5.21 (1H, m, H3'), 4.33–4.35 (3H, m, H4', H5'), 2.75 (1H, m, H2'$_A$), 2.67 (3H, s, CH_3-C(O)-NH), 2.17 (1H, m, H2'$_B$), 2.12 (3H, s, OAc), 2.09 (3H, s, OAc), 0.24 (9H, s, Si(CH_3)$_3$); ^{13}C-NMR ($CDCl_3$,

[4] Barthes, N.P.F., Karpenko, I.A., Dziuba, D., Spadafora, M., Auffret, J., Demchenko, A.P., Mely, Y., Benhida, R., Michel, B.Y., Burger, A., *RSC Adv.* **2015**, 5, 33536–33545.

50 MHz): δ = 172.0, 170.5, 170.2, 160.0, 152.9, 145.1, 104.6, 94.1, 92.9, 87.5, 83.1, 73.6, 63.6, 39.2, 26.6, 21.0, 21.0; HRMS (ESI$^+$): *m/z* calcd for $C_{20}H_{28}N_3O_7Si$ [M+H]$^+$: 450.1697; found: 450.1689.

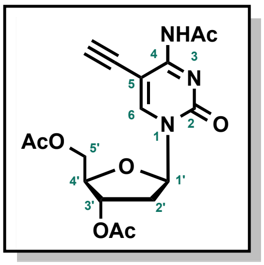

3′,5′-Di-*O*-acetyl-4-*N*-acetyl-5-ethynyl-2′-deoxycytidine (3): Et_4NF (650 mg, 3.9 mmol, 3 eq.) was added to a stirred solution of the alkyne **2** (1.3 mmol, 650 mg) in THF (13 mL). After 25 min, Et_2O was added and the resulting mixture was extracted with water (3 x), dried over $MgSO_4$, filtered and concentrated in vacuo. The residue was purified by flash chromatography on silica gel eluted with Toluene/Acetone (1:1 → 1:9, v/v) to provide the desired compound **3** as a white solid (386 g, 1.02 mmol, 79 %). $C_{17}H_{19}N_3O_7$ (377.35). R_f = 0.52 (Toluene/Acetone = 1:1); ^{1}H-NMR ($CDCl_3$,200 MHz): δ = 8.20 (1H, s, H6), 6.24 (1H, dd, ^3J=6.4 Hz, ^3J=6.4 Hz, H1’), 5.20–5.23 (1H, m, H3’), 4.38 (3H, s, H4’, H5’), 3.51 (1H, s, C≡C*H*), 2.81 (1H, ddd, ^2J=14.4 Hz, ^3J=5.8 Hz, ^4J=3.0 Hz, H2’$_A$), 2.71 (3H, s, *CH*$_3$-C(O)-NH), 2.18 (3H, s, OAc), 2.13 (3H, s, OAc), 2.13 (1H, m, H2’$_B$); ^{13}C-NMR ($CDCl_3$, 50 MHz): δ = 172.1, 170.6, 170.4, 160.2, 152.9, 146.0, 91.6, 87.7, 86.5, 83.3, 73.9, 63.7, 39.3, 26.8, 21.1, 21.0; HRMS (ESI$^+$): *m/z* calcd for $C_{17}H_{19}N_3NaO_7$: 400.1121; found: 400.1105.

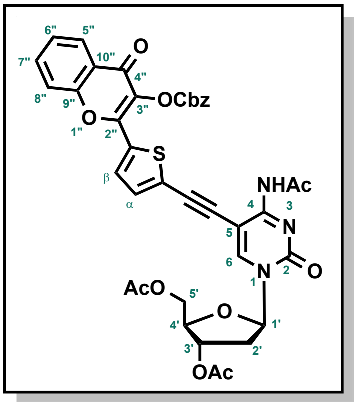

3′,5′-Di-*O*-acetyl-4-*N*-acetyl-5-(5-(3-(benzyloxycarbonyloxy)-4-oxo-chromen-2-yl)thien-2-yl)ethynyl-2′-deoxycytidine (4): To a stirred solution of the chromone **6** (0.31 mmol, 158 mg) and the terminal alkyne **3** (0.24 mmol, 100 mg) in dry DMF (8 mL) under argon, NEt_3 (5 eq, 166 µL, 1.2 mmol), $Pd(PPh_3)_2Cl_2$ (7 mol %) and CuI (7 mol %) were sequentially added. The mixture was warmed for 2 h at 55 °C and then cooled down to rt. The volatiles were reduced in vacuo and the resulting crude was purified by flash chromatography on silica gel eluted with Toluene/Acetone (1:11 → 1:5, v/v) to provide the desired compound **4** as a yellow solid (163 mg, 91 %). $C_{38}H_{31}N_3O_{12}S$ (737.74.81). R_f = 0.5 (Toluene/Acetone = 1:1); ^{1}H-NMR ^{1}H-NMR ($CDCl_3$, 200 MHz): δ = 8.26 (1H, s, H6), 8.24 (1H, m, H5”), 7.80 (1H, d, ^{3}J=4.1 Hz, Hα), 7.73 (1H, ddd, ^{3}J=8.5 Hz, ^{3}J=7.0 Hz, ^{4}J=1.6 Hz, H7”), 7.33–7.57 (9H, m, H8”, H6”, Hβ), 6.26 (1H, dd, ^{3}J=6.4 Hz, ^{3}J=6.0 Hz, H1’), 5.38 (2H, s, CH_2-Cbz), 5.23 (1H, m, H3’), 4.40 (3H, m, H4’, H5’), 2.82 (1H, ddd, ^{2}J=14.4 Hz, ^{3}J=5.8 Hz, ^{4}J=3.2 Hz, H2’$_A$), 2.72 (3H, s, *CH*$_3$-C(O)-NH), 2.24 (1H, m, H2’$_B$), 2.13 (3H, s, OAc), 2.11 (3H, s, OAc); ^{13}C-NMR ($CDCl_3$, 50 MHz): δ = 171.4, 170.4, 170.1, 159.3, 155.1, 151.8, 150.2, 145.7, 137.9, 134.5, 134.4, 133.8, 133.1, 132.3, 130.8, 129.1, 128.9, 128.8, 128.5, 128.3, 127.1, 126.2, 125.6, 125.4, 123.7, 118.0, 87.7, 83.2, 73.4, 71.5, 63.4, 39.2, 26.7, 21.5, 21.0, 20.9; HRMS (ESI$^+$): *m/z* calcd for $C_{38}H_{31}N_3O_{12}S$: 754.1707 [M+H]$^+$; found 754.1680.

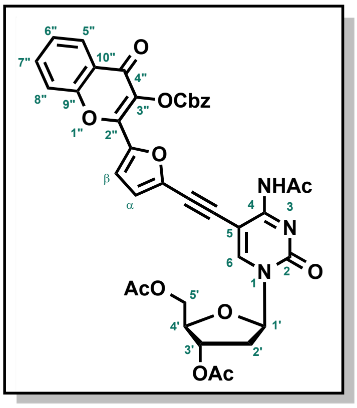

3′,5′-Di-*O*-acetyl-4-*N*-acetyl-5-(5-(3-(benzyloxycarbonyloxy)-4-oxo-chromen-2-yl)fur-2-yl)ethynyl-2′-deoxyuridine (5): To a stirred solution of the chromone **7** (0.31 mmol, 152 mg) and the terminal alkyne **3** (0.24 mmol, 100 mg) in dry DMF (8 mL) under argon, NEt_3 (5 eq, 166 µL, 1.2 mmol), $Pd(PPh_3)_2Cl_2$ (7 mol %), and CuI (7 mol %) were sequentially added. The mixture was warmed for 2 h at 55 °C and then cooled down to rt. The volatiles were reduced in vacuo and the resulting crude was purified by flash chromatography on silica gel eluted with Toluene/Acetone (1:11 → 1:2.5, v/v) to provide the desired compound **5** as a yellow solid (140 mg, 79 %). $C_{38}H_{31}N_3O_{13}$ (721.68). R_f = 0.5 (Toluene/Acetone = 1:1); ^{1}H-NMR ($CDCl_3$, 200 MHz): δ = 8.31 (1H, s, H6), 8.27 (1H, dd, ^{3}J=8.0 Hz, ^{4}J=1.3 Hz, H5”), 7.75 (1H, ddd, ^{3}J=8.4 Hz, ^{3}J=6.9 Hz, ^{4}J=1.5 Hz, H7”), 7.64 (1H, d, ^{3}J=8.0 Hz, H8”), 7.42 (1H, m, H6”), 7.15-7.24 (5H, m, CH-Cbz, Hα), 6.87 (1H, d, ^{3}J=3.7 Hz, Hβ), 6.26 (1H, dd, ^{3}J=6.4 Hz, ^{3}J=6.4 Hz, H1’), 5.35 (2H, s, CH_2-Cbz), 5.23–5.25 (1H, m, H3’), 4.39 (3H, s, H4’, H5’), 2.79 (1H, m, H2’$_A$), 2.72 (3H, s, *CH*$_3$-C(O)-NH), 2.27 (1H, m, H2’$_B$), 2.17 (3H, s, OAc), 2.13 (3H, s, OAc); ^{13}C-NMR ($CDCl_3$, 50 MHz): δ = 171.7, 170.5, 170.4, 169.8, 160.5, 155.1, 153.8, 152.4, 149.0, 147.4,143.7, 136.1, 134.6, 134.2, 132.3, 132.1, 129.1, 128.8, 128.7, 128.4, 128.3, 126.1, 125.4, 123.9, 118.3, 118.1, 113.5, 108.7, 107.3, 88.7, 83.5, 74.0, 71.3, 63.6, 39.4, 32.0, 30.4, 29.8, 29.8, 27.9, 22.8, 21.0, 21.0, 20.9; HRMS (ESI$^+$): *m/z* calcd for $C_{38}H_{31}N_3NaO_{13}$: 760.1755 [M+Na]$^+$; found 760.1754.

5-(5-(3-Hydroxy-4-oxo-chromen-2-yl)thien-2-yl)ethynyl-2′-deoxycytidine (13: TCC): To a stirred suspension of **11** (0.146 mmol, 110 mg) in MeOH (8.6 mL) was dropwise added a 33 % ammonium hydroxide aq. solution (5.2 mL). Protected from light, the reaction mixture was stirred at rt for 3 h and then acidified with AcOH (5.2 mL). After addition of milliQ® water (38 mL), the resulting solution was stored 12 h at 0 °C. The heterogeneous mixture was centrifuged for settling solid particles and the supernatant washings were carefully taken out with a syringe with a fine needle. The precipitate was triturated with acetonitrile, and after centrifugation the supernatant was removed by suction. The solid was dried under high vacuo to provide the desired compound **13** as a brown solid (34 mg, 47 %). $C_{24}H_{19}N_3O_7S$ (493.49). R_f= 0.17 (DCM/MeOH = 9:1). ^{1}H-NMR (DMSO-d^6, 500 MHz): δ = 8.37 (1H, s, H6), 8.11 (1H, dd, ^{3}J=8.0 Hz, ^{4}J=1.4 Hz, H5”), 7.90 (1H, d, ^{3}J=4.0 Hz, Hα), 7.81 (1H, ddd, ^{3}J=8.5 Hz, ^{3}J=7.1 Hz, ^{4}J=1.5 Hz, H7”), 7.72 (1H, d, ^{3}J=8.4 Hz, H8”), 7.56 (1H, d, ^{3}J=4.0 Hz, Hβ), 7.47 (1H, dd, ^{3}J=8.0 Hz, ^{3}J=7.0 Hz, H6”), 6.13 (1H, dd, ^{3}J=6.5 Hz, ^{3}J=6.0 Hz, H1’), 5.22 (1H, d, ^{3}J=4.3 Hz, 3’-O*H*), 5.14 (1H, t, ^{3}J=5.1 Hz, 5’-O*H*), 4.23 (1H, td, ^{3}J=6.7 Hz, ^{4}J=2.9 Hz, H3’), 3.81 (1H, q, ^{4}J=3.4 Hz, H4’), 3.66 (1H, ddd, ^{2}J=11.9 Hz, ^{3}J=5.0 Hz, ^{4}J=3.6 Hz, H5’$_A$), 3.56–3.61 (1H, m, H5’$_B$), 2.19 (1H, ddd, ^{2}J=13.3 Hz, ^{3}J=6.0 Hz, ^{4}J=3.8 Hz, H2’$_A$), 2.04 (1H, dt, ^{2}J=13.4 Hz, ^{3}J=6.8 Hz, H2’$_B$); ^{13}C-NMR (DMSO-d^6, 125 MHz): δ = 188.8, 164.8, 147.1, 146.8, 137.1, 125.5, 124.6, 120.1, 118.5, 116.6, 116.4, 113.7, 109.7, 79.6, 78.7, 62.1, 52.9, 33.1; HRMS (ESI$^-$): *m/z* calcd for $C_{24}H_{20}N_3O_7S$: 494.1022 [M+H]$^+$; found 494.1021.

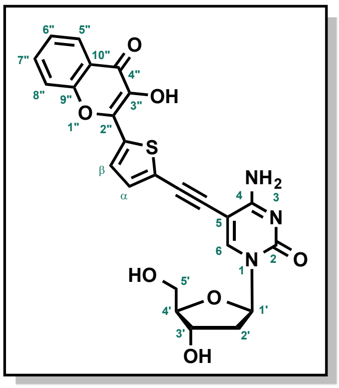

5-(5-(3-Hydroxy-4-oxo-chromen-2-yl)fur-2-yl)ethynyl-2′-deoxyuridine (14: FCC): To a stirred solution of **12** (0.136 mmol, 100 mg) in MeOH (8 mL) was dropwise added a 33 % ammonium hydroxide aq. solution (4.9 mL). Protected from light, the reaction mixture was stirred at rt for 1.5 h and then acidified with AcOH (4.8 mL). After addition of milliQ® water (35 mL), the resulting solution was stored 12 h at 0 °C. The heterogeneous mixture was centrifuged for settling solid particles and the supernatant washings were carefully taken out with a syringe with a fine needle. The precipitate was triturated with acetonitrile, and after centrifugation the supernatant was removed by suction. The final product was dried under high vacuo to provide the desired compound **14** as a brown solid (33 mg, 51 %). ($C_{24}H_{19}N_3O_8$ (477.43). R_f= 0.20 (CH_2Cl_2/MeOH = 9:1). ^{1}H-NMR (MeOD-d^3, 500 MHz): δ = 8.59 (1H, s, H6), 8.18 (1H, ddd, ^{3}J=8.0 Hz, ^{4}J=1.6 Hz, ^{5}J=0.4 Hz, H5”), 7.78 (1H, ddd, ^{3}J=8.6 Hz, ^{3}J=7.0 Hz, ^{4}J=1.6 Hz, H7”), 7.68 (1H, dd, ^{3}J=8.5 Hz, ^{5}J=0.5 Hz, H8”), 7.46 (1H, ddd, ^{3}J=8.0 Hz, ^{3}J=7.0 Hz, ^{4}J=1.0 Hz, H6”), 7.41 (1H, d, ^{3}J=3.7 Hz, Hα), 7.01 (1H, d, ^{3}J=3.7 Hz, Hβ), 6.24 (1H, t, ^{3}J=6.2 Hz, H1’), 4.40 (1H, dt, ^{3}J=6.2 Hz, ^{4}J=4.1 Hz, H3’), 3.98 (1H, q, ^{4}J=3.5 Hz, H4’), 3.89–3.86 (1H, m, H5’A), 3.77 (1H, dd, ^{2}J=12.1 Hz, ^{4}J=3.5 Hz, H5’$_B$), 2.44 (1H, ddd, ^{2}J=13.6 Hz, ^{3}J=6.2 Hz, ^{4}J=4.2 Hz, H2’$_A$), 2.20 (1H, dt, ^{2}J=13.6 Hz, ^{3}J=6.4 Hz, H2’$_B$); ^{13}C-NMR (MeOD-d^3, 125 MHz): δ = 174.1, 165.8, 156.6, 156.3, 147.2, 146.8, 140.3, 139.5, 139.5, 135.0, 126.1, 125.9, 123.2, 119.5, 119.4, 117.8, 91.3, 89.2, 88.2, 87.8, 85.2, 71.6, 62.3, 42.6; HRMS (ESI$^+$): *m/z* calcd for $C_{24}H_{20}N_3O_8$: 478.1250 [M+H]$^+$; found 478.1240.

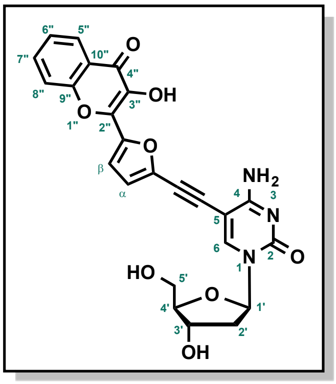

1.3 Preparation of the emissive amidite 14

5-Iodo-4-*N*-acetyl-2’-deoxycytidine (15): To a stirred solution of 5-iodo-2'-deoxycytidine (7.00 g, 19.8 mmol), in dry pyridine (25 mL), previously cooled down to 0 °C, was dropwise added Me_3SiCl (10.4 mL, 79.3 mmol, 4 eq). Protected from light, the reaction mixture was stirred at rt for 3 h. After completion by monitoring with TLC, the reaction mixture was cooled down to 0 °C. Et_3N (13.3 mL, 95.2 mmol, 4.8 eq) and acetic anhydride (4.5 mL, 47.6 mmol, 2.4 eq) were sequentially added. The reaction mixture was stirred at rt overnight. H_2O (40 mL) was added and the mixture was stirred for 15 min. The organic phase was extracted with CH_2Cl_2 (2 x) and EtOAc (2x), dried over $MgSO_4$, filtered and reduced *in vacuo*. The residue was purified by flash chromatography on silica gel eluted with Toluene/Acetone (9:1 → 3:2, v/v) to provide the desired compound **9** as a white foam (2.5 g, 32%). $C_{11}H_{14}IN_3O_5$ (395.2). R_f=0.13 (Toluene/Acetone = 1:1). ^{1}H-NMR (DMSO-d^6, 200 MHz): δ = 9.46 (1H, s, N*H*-CO), 8.72 (1H, s, H6), 6.02 (1H, t, ^{3}J=5.8 Hz, H1'), 5.30–5.21 (2H, m, 3'-O*H* & 5'-O*H*), 4.25–4.20 (1H, m, H3'), 3.85 (1H, m, H4'), 3.71–3.54 (2H, m, H5'), 2.35–2.03 (5H, m, H2' & OAc); ^{13}C-NMR (DMSO-d^6, 50 MHz): δ =169.9, 169.8, 161.6, 161.4, 153.1, 150.7, 87.9, 86.6, 69.2, 69.1, 60.9, 60.7, 60.3, 24.5; MS (ESI$^+$, MeOH) *m/z*: 812.9 [2M+Na]$^+$.

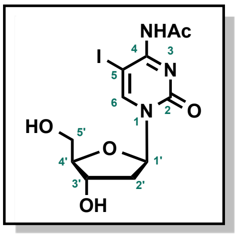

5’-*O*-(4,4’-Dimethoxytrityl)-5-iodo-4-*N*-acetyl-2’-deoxycytidine (16): To a stirred solution of **15** (2.5 g, 6.3 mmol), in dry DMF (26 mL) previously cooled down to 0 °C were sequentially added DIPEA (5.7 mL, 31.6 mmol, 5 eq) and DMTrCl (2.8 g, 8.2 mmol, 1.3 eq). Protected from light, the reaction mixture was stirred at rt overnight. The volatiles were reduced *in vacuo* and the residue was purified by flash chromatography on silica gel eluted with Toluene/Acetone (93:7 → 82:18, v/v) to provide the desired derivative **16** as a beige foam (2.50 g, 54 %). $C_{32}H_{32}IN_3O_7$ (697.5). R_f=0.48 (Toluene/Acetone = 1:1). ^{1}H-NMR (CD_2Cl_2, 200 MHz): δ = 8.40 (1H, s, H6), 7.47–7.43 (2H, m, *m-H*-Ph), 7.35 (4H, d, ^3J=8.8 Hz, *o-H*-PhOMe), 7.29–7.17 (3H, m, *o-H*-Ph, *p-H*-Ph), 6.87 (4H, d, ^3J=8.8 Hz, *m-H*-PhOMe), 6.24 (1H, t, ^3J=6.0 Hz, ^3J=6.6 Hz, H1'), 4.56 (1H, m, H3'), 4.17 (1H, m, H4'), 3.80 (6 H, s, OMe), 3.37 (2H, m, H5'), 2.75–2.52 (4H, m, H2’$_A$, Ac), 2.31–2.21 (1H, m, H2’$_B$); ^{13}C-NMR ($CDCl_2$, 50 MHz): δ = 158.7, 144.6, 137.9, 135.5, 135.4, 130.0, 130.0, 128.9, 128.1, 128.0, 128.0, 126.9, 125.2, 113.3, 86.9, 72.0, 63.3, 55.2, 42.1, 21.1; MS (ESI$^+$, MeOH) *m/z*: 812.9 [M+H]$^+$.

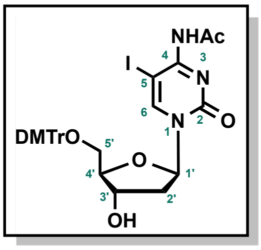

5’-*O*-(4,4’-Dimethoxytrityl)-5-trimethylsilylethynyl-4-*N*-acetyl-2’-deoxycytidine (17): To a stirred solution of **16** (2.3 g, 3.3 mmol) in dry THF (11 mL) under argon, were sequentially added NEt_3 (2.8 mL, 19.8 mmol, 6 eq), TMS-acetylene (612 µL, 4.3 mmol, 1.3 eq), and CuI (7 mol%, 44 mg)/$PdCl_2(PPh_3)_2$ (7 mol%, 164 mg). The reaction mixture was warmed to 55 °C and stirred for 2 h. The mixture was filtered over a 545 Celite® (basic-washed) pad and the volatiles were removed *in vacuo*. The residue was purified by flash chromatography on silica gel eluted with Toluene/Acetone (93:7 → 55:45, v/v) to provide the desired compound **17** as a beige foam (1.64 g, 74 %). $C_{37}H_{41}N_3O_7Si$ (667.8). R_f=0.57 (Toluene/Acetone = 1:1). ^{1}H-NMR (CD_2Cl_2, 200 MHz): δ = 8.33 (1H, s, H6), 8.12 (1H, s, N*H*-CO-), 7.44 (2H, d, ^3J=6.8 Hz, *m-H*-Ph), 7.38–7.20 (10H, m), 6.85 (4H, d, ^3J=8.8 Hz, *m-H*-PhOMe), 6.20 (1H, dd, ^3J=6.4 Hz, 6.4 Hz, H1'), 4.53 (1H, m, H3'), 4.23 (1H, m, H4'), 3.78 (6H, s, OMe), 3.30 (2H, m, H5'), 2.77 (1H, ddd, ^2J=13.8 Hz, ^3J=6.3 Hz, ^3J=2.9 Hz, H2'$_A$), 2.60 (3H, s, Ac), 2.24 (1H, dt, ^3J=13.8 Hz, ^2J=6.3 Hz, H2'$_B$), 0.14 (9 H, s, ($CH_3)_3$Si-); ^{13}C-NMR (CD_2Cl_2, 50 MHz): δ = 171.4, 160.4, 159.0, 159.0, 145.9, 145.0, 136.0, 135.8, 130.31, 130.3, 129.3, 128.5, 128.4, 128.2, 127.2, 125.6, 113.6, 104.4, 94.4, 88.4, 87.6, 87.2, 72.5, 63.8, 55.5, 42.6, 26.4, -0.3; HRMS (ESI$^+$): *m/z* calcd for $C_{37}H_{42}N_3O_7Si$: 668.2792 [M+H]$^+$; found 668.2786.

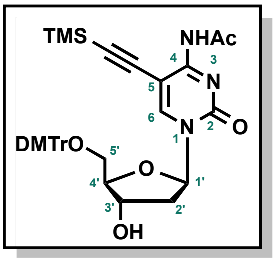

5’-*O*-(4,4’-Dimethoxytrityl)-4-*N*-acetyl-5-ethynyl-2’-deoxycytidine (18): To a stirred solution at 0 °C of the protected ethynyl derivative **17** (1.60 g, 2.4 mmol) in THF (16 mL) was portionwise added $Et_4NF{\cdot}H_2O$ (1.40 g, 9.6 mmol, 4 eq). The reaction mixture was stirred at rt for 3.5 h and then quenched with water. The organic layer was extracted with Et_2O (1 x) and CH_2Cl_2 (2 x), dried over $MgSO_4$, filtered and reduced *in vacuo* to provide the desired compound **18** as a beige foam (1.40 g, 99 %). The compound was sufficiently pure to be used in the next step without further purification. $C_{34}H_{33}N_3O_7$ (595.7). R_f=0.22 (Toluene/Acetone = 7:3, v/v). ^{1}H-NMR (CD_2Cl_2, 200 MHz): δ = 8.45 (1H, s, H6), 8.12 (1H, s, N*H*-CO-), 7.49–7.13 (14H, m), 6.90–6.85 (4H, m, *m-H*-PhOMe), 6.23 (1H, t, ^3J=6.0 Hz, H1'), 4.60 (1H, s, H3'), 4.25 (1H, m, H4'), 3.80 (6H, s, OMe), 3.37 (3H, m, H5’, *H*C≡C-), 2.85–2.76 (1H, m, H2'$_A$), 2.62 (3H, s, Ac), 2.27–2.35 (1H, m, H2'$_B$); ^{13}C-NMR (CD_2Cl_2, 50 MHz): δ = 160.4, 159.0, 147.2, 145.0, 138.3, 136.0, 135.7, 130.35, 130.3, 129.3, 128.5, 128.4, 128.20, 127.2, 125.6, 113.6, 88.3, 87.5, 87.2, 73.8, 72.1, 63.7, 55.6, 42.5, 30.4, 30.0, 26.5, 21.5. MS (ESI$^+$, MeOH) *m/z*: 596.4 [M+H]$^+$.

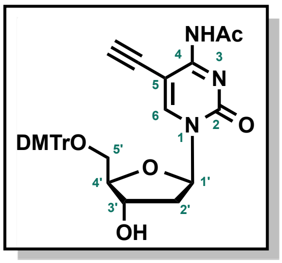

5’-*O*-(4,4’-Dimethoxytrityl)-3-*N*-acetyl-5-(5-(3-benzylcarbonate-4-oxo-chromen-2-yl)thien-2-yl)ethynyl-2’-deoxycytidine (19): To a stirred solution of **18** (186 mg, 0,31 mmol, previously azeotropically coevaporated with dry toluene) and chromone **6** (195 mg, 0.41 mmol, 1.3 eq) in dry DMF (6.2 mL) under argon, were sequentially added NEt_3 (218 µL, 1.56 mmol, 5 eq), and CuI (7 mol %, 0.022 mmol, 4 mg,)/$PdCl_2(PPh_3)_2$ (7 mol %, 0.022 mmol, 15 mg) all together. The reaction mixture was warmed to 55 °C for 30 min. EtOAc (15 mL) was added at 0 °C followed by a 10 % LiCl aq. solution (15 mL). The organic phase was extracted with EtOAc (2 x), dried over $MgSO_4$, filtrated and concentrated under vacuum. The obtained residue was purified by preparative TLC on silica gel eluted with CH_2Cl_2/EtOAc (3:7) to provide the desired compound **19** as a dark yellow foam (142 mg, 47 %). $C_{55}H_{45}N_3O_{12}S$ (972.0). R_f=0.47 (CH_2Cl_2/MeOH=9.5:0.5). ^{1}H-NMR (acetone-d^6, 200 MHz): δ = 8.63 (1H, s, H6), 8.16 (1H, d, ^3J=7.9 Hz, H5"), 7.88 (2H, m, H7", Hβ), 7.78 (1H, d, ^3J=7.9 Hz, H8"), 7.56–7.11 (14H, m, H6", *m-H*-Ph, *o-H*-PhOMe, *o-H*-Ph, *p-H*-Ph), 7.11 (1H, m, Hα), 6.86 (4H, m, *m-H*-PhOMe), 6.20 (1H, m, H1'), 5.36 (2H, s, C*H*$_2$-Cbz), 4.69–4.59 (1H, m, H3'), 4.23 (1H, m, H4'), 3.76–3.71 (6H, s, OMe), 3.40 (2H, m, H5'), 2.63–2.47 (5H, m, H2’, Ac); ^{13}C-NMR (acetone-d^6, 50 MHz): δ = 171.5, 159.6, 156.0, 152.5, 151.0, 148.0, 145.8, 136.8, 136.5, 135.9, 135.5, 134.4, 132.7, 131.7, 130.9, 130.8, 129.5, 129.2, 129.1, 129.0, 128.9, 128.8, 128.7, 128.6, 127.7, 126.5, 126.2, 124.3, 119.1, 114.0, 88.8, 88.2, 87.6, 71.8, 64.2, 55.5, 42.8, 26.4; HRMS (ESI): m/z calcd for $C_{55}H_{46}N_3O_{12}S$: 972.2802 [M+H]$^+$; found 972.2795.

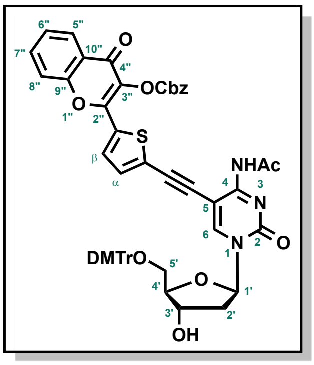

5'-*O*-(4,4'-Dimethoxytrityl)-3-*N*-acetyl-5-(5-(3-benzylcarbonate-4-oxo-chromen-2-yl)thien-2-yl)ethynyl-2'-deoxycytidine, 3'-[(2-cyanoethyl)-*N*,*N*-diisopropyl]-phosphoramidite (20): To a stirred solution of **19** (0.21 mmol, 200 mg, previously dried azeotropically by coevaporation with dry toluene) in CH_2Cl_2 (3 mL) under argon and cooled down at 0 °C, were sequentially added DIPEA (0.54 mmol, 95 µL, 2,6 eq) and 2-cyanoethyl-*N*,*N*-diisopropylchlorophosphoramidite (0.27 mmol, 60 µL, 1.3 eq). The reaction mixture was stirred at rt for 2.5 h. The volatiles were removed in vacuo and the residue was purified by flash chromatography on silica gel eluted with CH_2Cl_2/Et_2O (95:5 → 80:20, v/v) to provide the desired compound **20** as a orange foam (144 mg, 60 %). $C_{64}H_{62}N_5O_{13}PS$ (1172.26). R_f=0.68 (CH_2Cl_2/EtOAc=7:3). ^{1}H-NMR (CD_3CN, 200 MHz): δ = 8.53 (1H, s, H6), 8.45 (1H, s, N*H*-CO), 8.13 (1H, dd, ^3J=8.0 Hz, ^4J=1.5 Hz, H5"), 7.88–7.78 (2H, m, H7", Hβ), 7.68 (1H, d, ^3J=8.0 Hz, H8"), 7.54–7.16 (17H, m, H6", *m-H*-Ph, *o-H*-PhOMe, *o-H*-Ph, *p-H*-Ph), 7.01 (1H, m, Hα), 6.84–6.78 (4H, m, *m-H*-PhOMe), 6.09 (1H, m, H1'), 5.30 (2H, s, C*H*$_2$-Cbz), 4.75–4.65 (1H, m, H3'), 4.22 (1H, m, H4'), 3.75 (1H, m, N-C*H*(CH_3)$_2$), 3.69 (6H, s, OMe), 3.63–3.41 (2H, m, O-C*H*$_2$-CH_2-CN), 3.38–3.31 (2H, m, H5'), 2.69–2.51 (3H, m, H2', O-CH_2-C*H*$_2$-CN), 2.45 (3H, s, Ac), 1.19–0.98 (12H + *26H for the excess of hydrolyzed reagent*, m, N-CH(C*H*$_3$)$_2$); ^{31}P-NMR (CD_3CN, 81 MHz): δ = 148.1, 147.9, *13.7 (excess of hydrolyzed reagent)*; HRMS (ESI): m/z calcd for $C_{64}H_{63}N_5O_{13}PS$: 1172.3881 [M+H]$^+$; found 1172.3887.

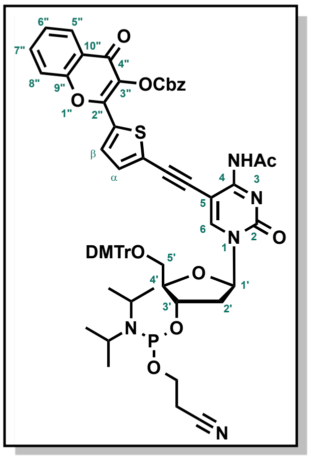

2. Spectroscopic studies of dual-emissive nucleosides (TCC & FCC)

2.1. Purification of the free nucleosides

Fig S1. HPLC profiles of TCC (left) and FCC (right) at 260 nm (Top) and 390 nm (Bottom)

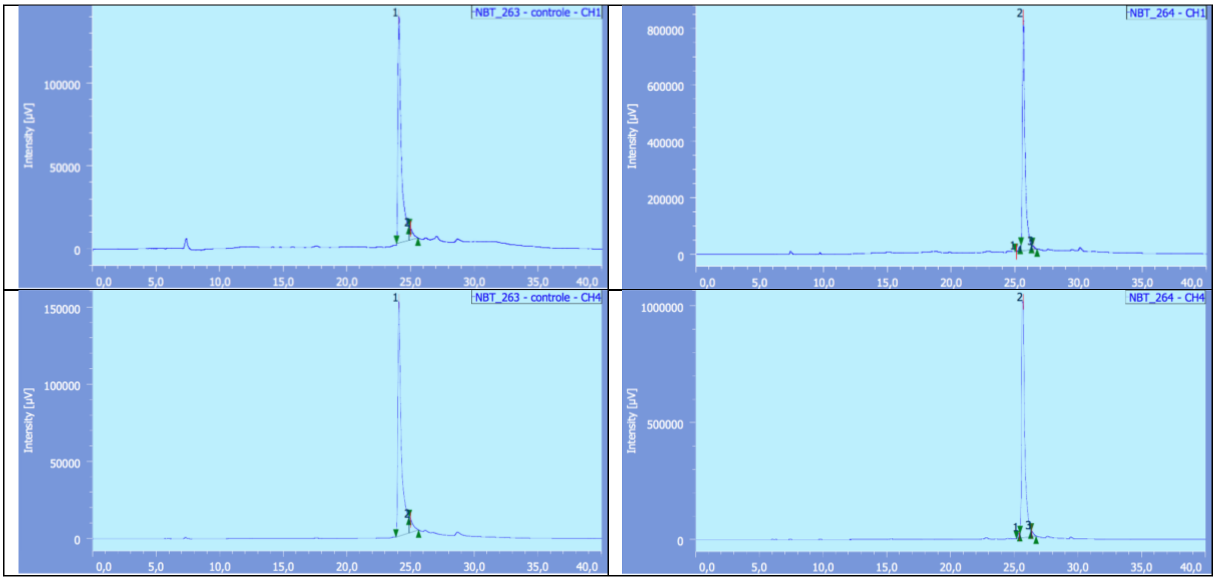

2.2. Steady-state fluorescence measurements

Fig S2. Fluorescence emission spectra of TCC in different solvent polarities

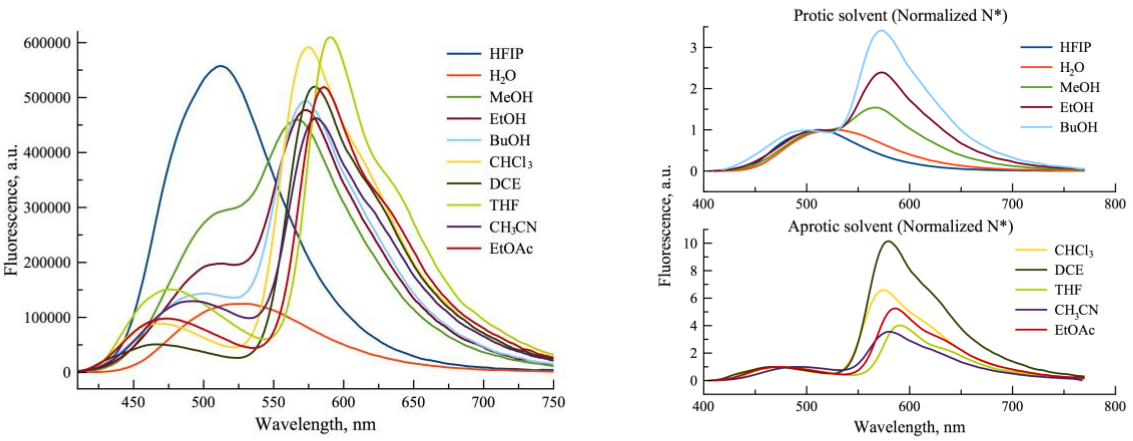

Fig S3. Fluorescence emission spectra of FCC in different solvent polarities

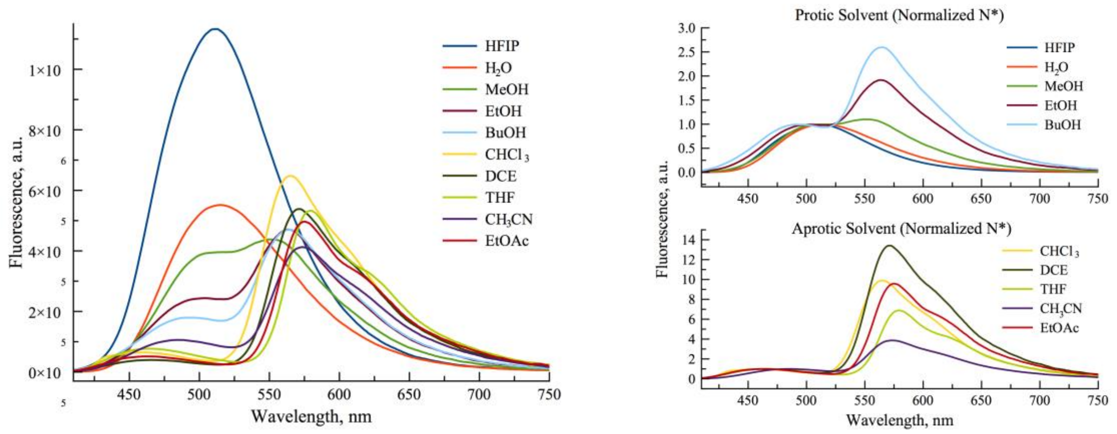

Table S1. Spectroscopic properties of 3HC-labelled cytidine analogues **FCC** & **TCC**.

Solvent	$E_T(30)$[a]	$\Sigma\beta_2$[Hb]	λ_{Abs}[c]		λ_{N*}[d]		λ_{T*}[e]		I_{N*}/I_{T*}[f]		Φ[g]	
			**TCC**	**FCC**	**TCC**	**FCC**	**TCC**	**FCC**	**TCC**	**FCC**	**TCC**	**FCC**
**HFIP**	65.3	0.10	394 (395)	387 (388)	511 (513)	509 (511)	-	-	-	-	0.25 (0.21)	0.35 (0.39)
**H_2O**	63.1	0.35	396 (398)	389 (391)	528 (529)	514 (515)	-	-	-	-	0.06 (0.06)	0.28 (0.25)
**MeOH**	55.4	0.47	393 (391)	385 (384)	516 (517)	509 (514)	569 (568)	553 (551)	0.64 (0.66)	0.87 (0.90)	0.22 (0.21)	0.24 (0.23)
**EtOH**	51.9	0.48	395 (397)	387 (385)	510 (512)	501 (502)	575 (575)	562 (564)	0.36 (0.38)	0.56 (0.51)	0.21 (0.19)	0.21 (0.24)
**BuOH**	49.7		399 (396)	390 (390)	499 (499)	490 (492)	573 (575)	564 (564)	0.29 (0.28)	0.39 (0.38)	0.22 (0.22)	0.17 (0.22)
**CH_3CN**	45.6	0.32	392 (390)	381 (381)	491 (493)	484 (484)	580 (581)	573 (571)	0.29 (0.28)	0.26 (0.26)	0.22 (0.19)	0.16 (0.18)
**DCE**	41.3	0.11	394 (393)	384 (385)	470 (466)	465 (466)	578 (581)	572 (570)	0.12 (0.1)	0.08 (0.08)	0.18 (0.23)	0.21 (0.23)
**$CHCl_3$**	39.1	0.02	398 (396)	389 (389)	466 (467)	480 (462)	574 (576)	566 (564)	0.15 (0.15)	0.10 (0.10)	0.25 (0.26)	0.28 (0.27)
**EtOAc**	38.1	0.45	394 (393)	383 (384)	474 (475)	466 (463)	588 (857)	575 (576)	0.20 (0.19)	0.11 (0.11)	0.18 (0.19)	0.19 (0.20)
**Dioxane**	36.0	0.64	397 (398)	388 (388)	467 (468)	454 (457)	587 (587)	576 (577)	0.39 (0.38)	0.20 (0.20)	0.18 (0.25)	0.28 (0.28)
**THF**	36.2		398 (399)	386 (388)	475 (478)	464 (464)	590 (591)	579 (580)	0.25 (0.26)	0.15 (0.15)	0.25 (0.23)	0.22 (0.22)
**DME**	38.2		395 (396)	386 (385)	478 (479)	464 (466)	587 (589)	578 (577)	0.28 (0.24)	0.15 (0.16)	0.15 (0.20)	0.22 (0.23)

Footnotes: a) Reichardt's empirical solvent polarity index;[5] b) H-bond basicity;[6] c) Position of the absorption maximum; d) Position of the emission maximum of the normal N* band; e) Position of the emission maximum of the tautomer T* band ; f) I_{N*}/I_{T*} corresponds to the intensity ratio of the two emission bands at their maxima, 2.5% mean standard deviation; e) Quantum

[5] C. Reichardt, *Chem. Rev.* **1994**, *94*, 2319–2358.

[6] *a*) A. S. Klymchenko, A. P. Demchenko, *Phys. Chem. Chem. Phys.* **2003**, *5*, 461. *b*) M. H. Abraham, *J. Phys. Org. Chem.* **1993**, *6*, 660–684.

yields determined using 3-hydroxyflavone in toluene (Φ=0.29)[7] or 4'-(dimethylamino)-3-hydroxyflavone in ethanol (Φ=0.27)[8] or quinine sulfate in 0.1 M H_2SO_4 (Φ=0.54)[9] as references;

2.3. Hydration studies on the emissive nucleosides TCC & FCC

Fig S4. TCC: Variation of water % in acetonitrile (left) & dioxane (right)

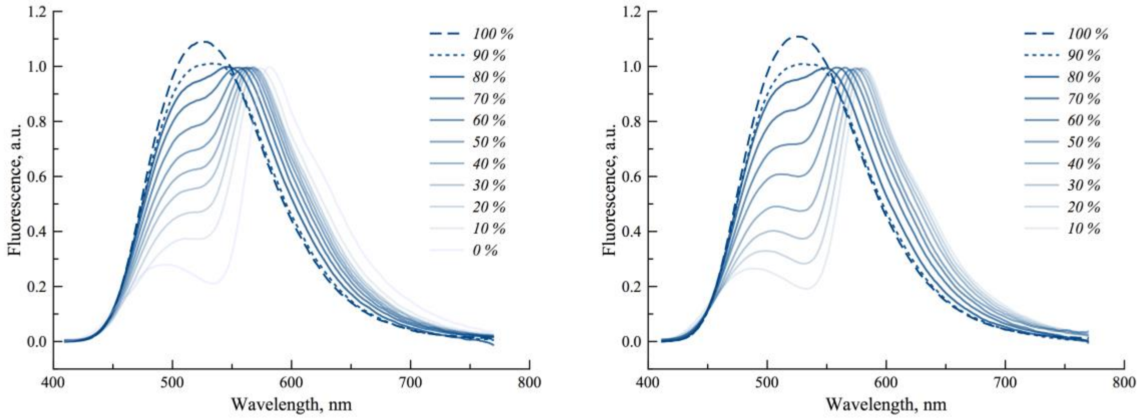

Fig S5. FCC: Variation of water % in acetonitrile (left) & dioxane (right)

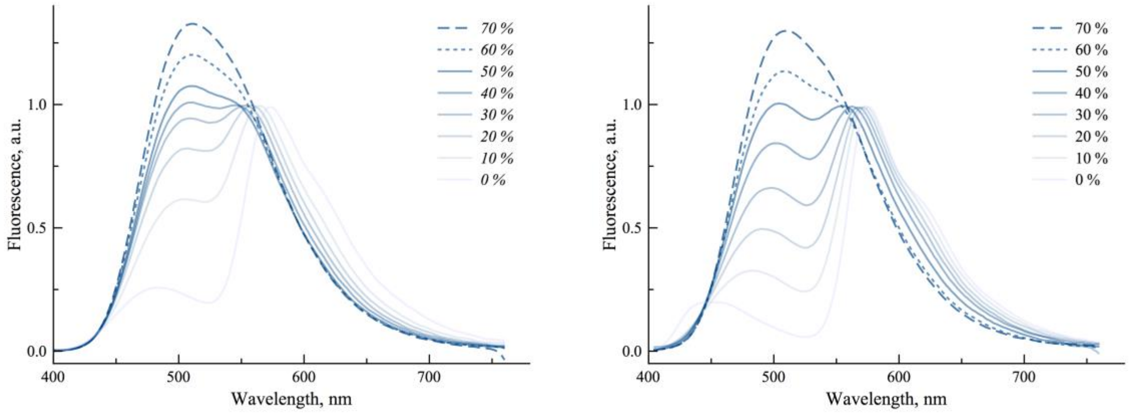

Fig S6. I_{N^*}/I_{T^*} vs. Water % in acetonitrile (left) and dioxane (right) for TCC (blue) and FCC (red)

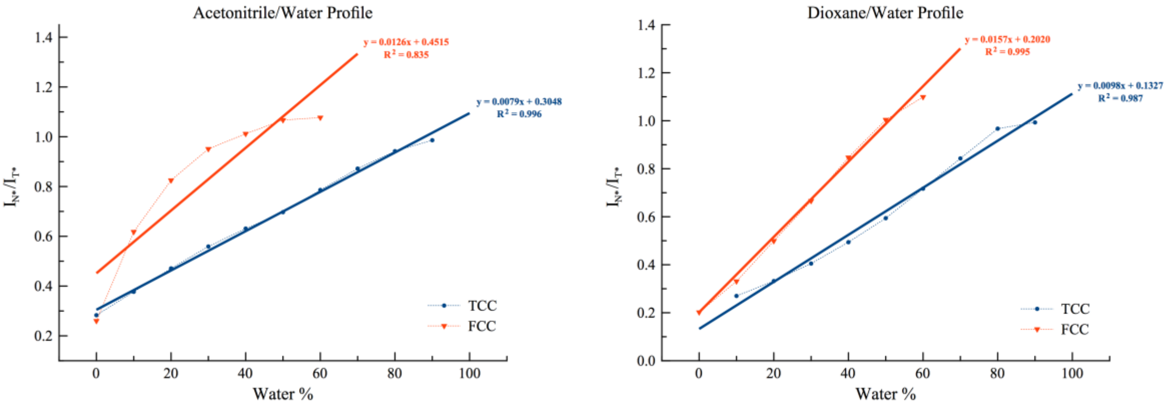

---

[7] A. S. Klymchenko, T. Ozturk, V. G. Pivovarenko and A. P. Demchenko, *Can. J. Chem.* **2001**, *79*, 358–363.
[8] S. M. Ormson, R. G. Brown, F. Vollmer and W. Rettig, *J. Photochem. Photobiol. A: Chem.* **1994**, *81*, 65–72.
[9] W. H. Melhuish, *J. Phys. Chem.* **1961**, *65*, 229–235.

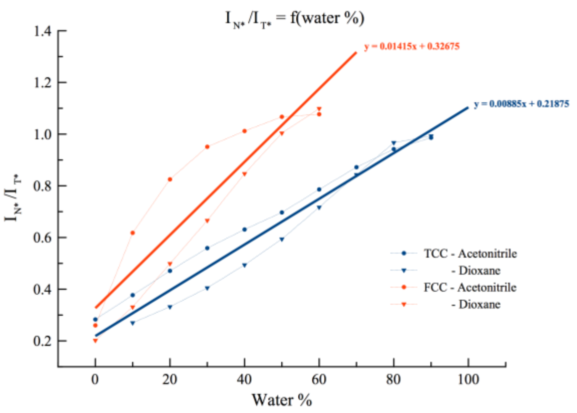

Fig S7. Dependence of the Stokes shift of TCC (left) and FCC (right) on the orientation polarizability function Δf (Lippert's parameter)

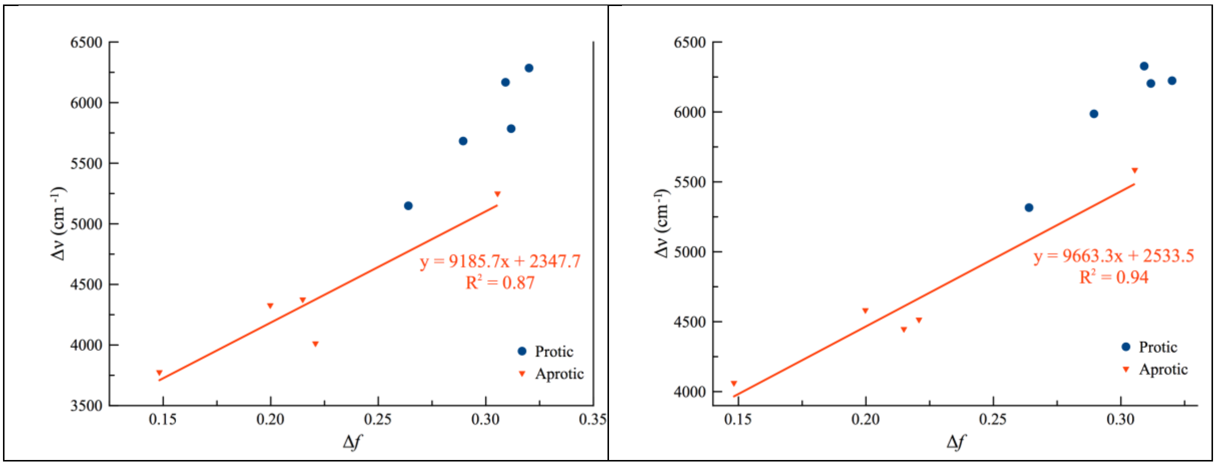

The linear trends were obtained only with aprotic solvents according to the Lippert's equation. Values out of the fitting represent protic solvents. Correlation factors R^2 are respectively 0.87 (**TCC**) and 0.94 (**FCC**).

3. ODN synthesis, purification & mass characterizations

3.1. ODN synthesis and purification

The ODN synthesis was performed on an Expedite 8900 DNA synthesizer (Applied Biosystem) using the "trityl off" mode and ultra-mild Pac phosphoramidite chemistry on a 0.2 µmol scale. Reagents and solvents, as well as dT, Ac-dC, Pac-dA, and iPr-Pac-dG (or dmf-dG) phosphoramidites were purchased from Link Technologies and Chemgenes. The standard DNA assembly protocol "DMT-off" was employed except for the following modifications: 5-Ethylthio-1H-tetrazole (ETT) was used as activating agent; Pac-anhydride was used for capping; a longer coupling time (1200 s) was applied to the 3HC phosphoramidite. Non-labeled ODNs used as wild-type sequences were purchased from Microsynth AG. The ODNs were cleaved from the solid support and deprotected with concentrated aqueous ammonia at room temperature for 12 h. The ODNs were analyzed (0.5 mL/min) and purified (2.5 mL/min) by RP HPLC (HPLC apparatus: WatersTM 600 Controller with WatersTM 996 Photodiode Array Detector and Jasco LC-Net II / ADC apparatus. Columns: analytical, 300 × 4.60 mm, 5 µm particle size, Clarity® 100Å, Phenomenex®; semi-preparative, Clarity® 5µ Oligo-RP column 250 x 10 mm Phenomenex®). The following gradient system was used: 100 % A –(30 min)→ 60 % A / 40 % B –(5 min)→ 100 % B –(5 min)→ 100 % A with A=Buffer pH 7.0 (1.9 L of MilliQ® water, 160 mL acetonitrile, 28 mL triethylamine, 12 mL of acetic acid) and B=0.2 CH_3CN:0.8 Buffer.

Fig S8. HPLC profile of TMT single strand ODN (390 nm)

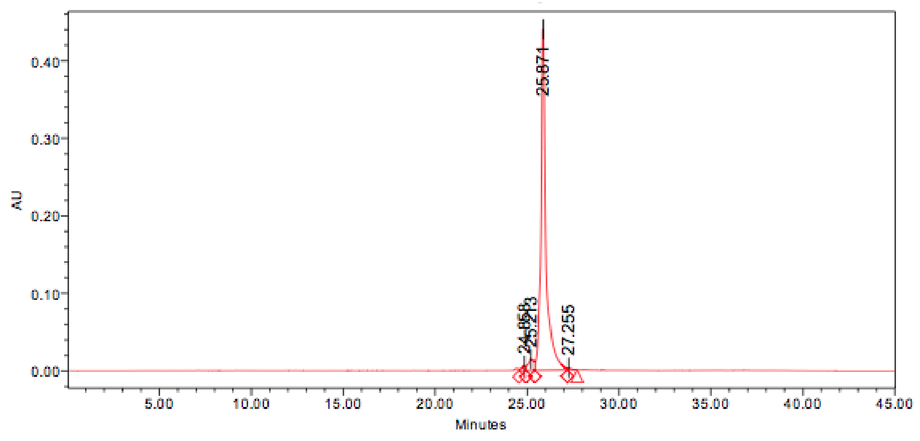

Fig S9. HPLC profile of AMA single strand ODN (390 nm)

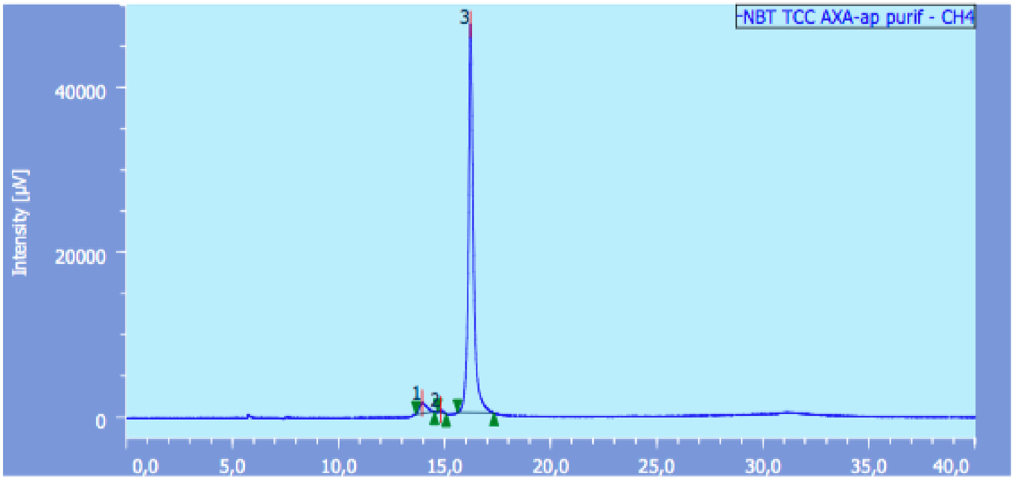

Fig S10. HPLC profile of CMC single strand ODN (390 nm)

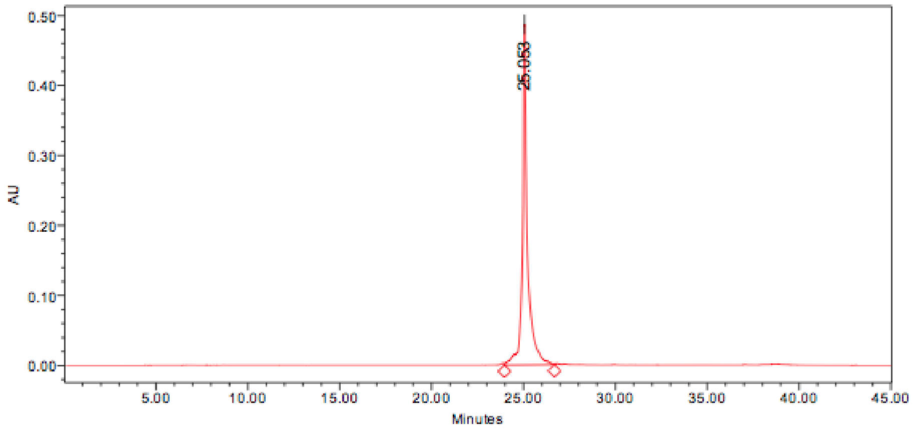

Fig S11. HPLC profile of GMG single strand ODN (390 nm)

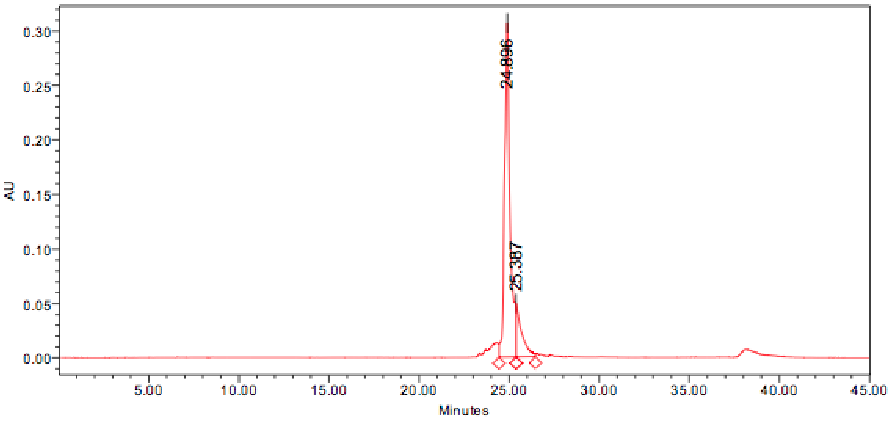

Fig S12. HPLC profile of TMA i-motif ODN (390 nm)

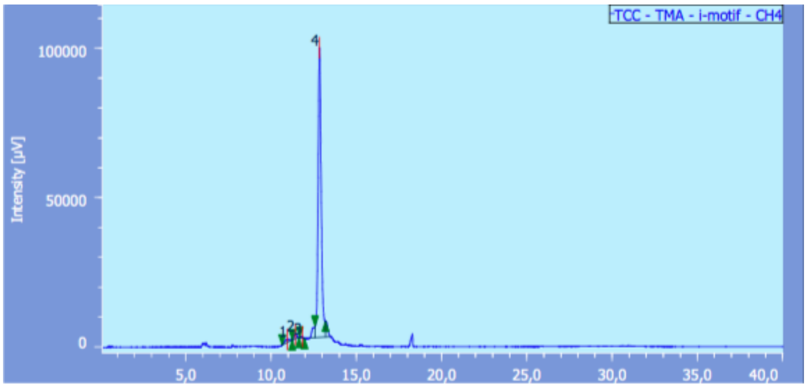

3.2. MALDI TOF/TOF analysis of ODNs

Dibasic Ammonium Citrate (DAC) (98% capillary GC) and acetonitrile (HPLC grade) was purchased from Sigma-Aldrich. Ultrapure 3-Hydroxypicolinic Acid (3-HPA) MALDI matrix was obtained from Protea Biosciences. C4 pipette tips (Zip-Tip) were from Millipore. The samples (500 pmol) were diluted to 10 µL of water and were desalted with a C4 pipette Tips (Zip-tip). The Zip-tip was activated before use with 2 x 5 µL of water: CH_3CN (50:50) and 2 x 5µL of DAC (50 mg/ml diluted in water). The 10 µL of the ODN solution were loaded on Zip-tip by drawing and expelling ten times. Next, the zip-tip was washed with 3 x 5 µL of DAC (50 mg/mL) and 3 x 5 µL of water. Elution was performed with 1.5 µL of 3-HPA matrix (80 mg/mL, 50:50 CH_3CN:DAC) directly on MALDI plate. The ODN profile obtained in a AB Sciex MALDI-TOF/TOF mass spectrometer in reflector mode with external calibration mixture (cal Mix 1+2 distributed by AB Sciex). MALDI-TOF/TOF-MS analysis: MS spectra were recorded manually in a mass range of 500-6000 Da resulting from 400 laser shots of constant intensity fixed at 6200. Data were collected using 4000 series Explorer (AB Sciex) experiments.

Table S2. Mass of the labeled single strand DNA.

ODN	Sequence	MALDI-TOF found (calcd) $[M^+]$
**TMT**	5′-CGTTTTT**M**TTTTTGC-3′	4775.7 (4772.4)
**AMA**	5′-CGTTTTA**M**ATTTTGC-3′	4809.3 (4790.2)
**CMC**	5′-CGTTTTC**M**CTTTTGC-3′	4746.3 (4742.2)
**GMG**	5′-CGTTTTG**M**GTTTTGC-3′	4832.1 (4822.2)
**TMA**	5’-CCCTAACCCT**M**ACCCTAACCC-3’	6438.2 (6441.3)

Fig S13. MALDI-TOF mass spectrograms of labeled ODN sequences (AMA, TMT, CMC, GMG and TMA)

AMA

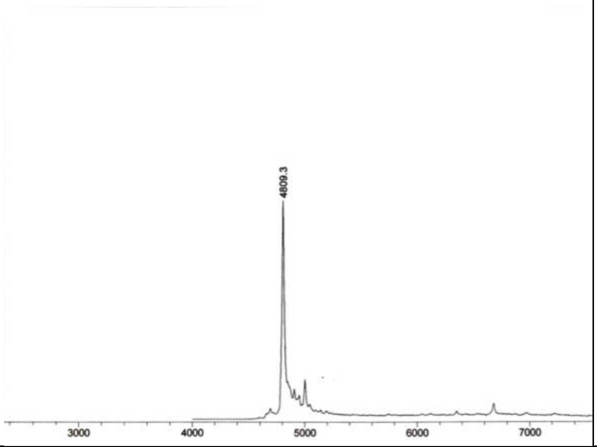

TMT

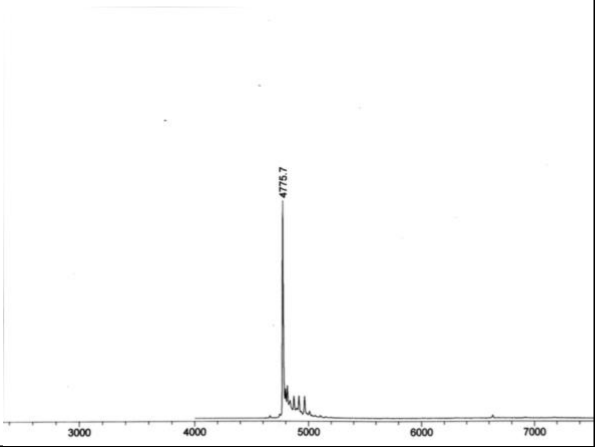

CMC

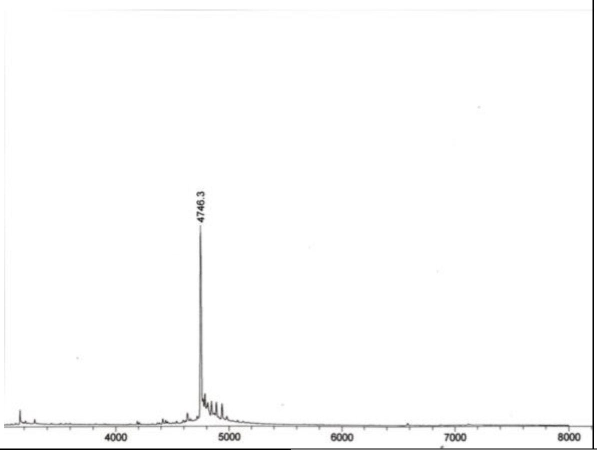

GMG

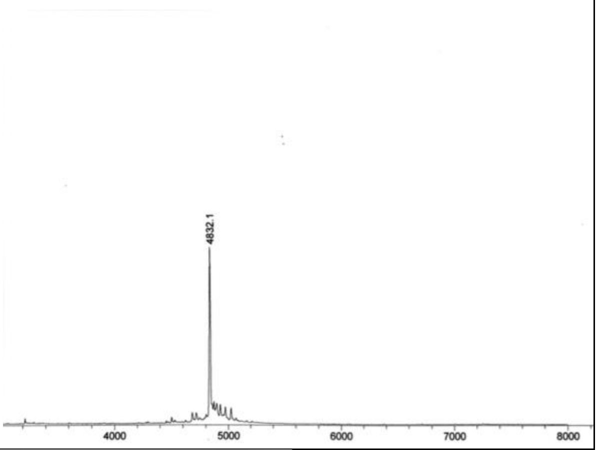

TMA

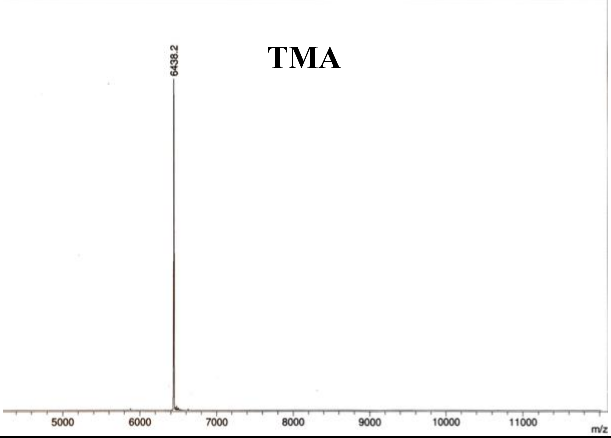

4. Spectroscopic characterizations of wild-type and labeled ODNs

4.1 Preparation of the samples and buffers

The model ODNs were analyzed in duplicate in cacodylate buffer pH 7.0 (10 mM cacodylate, 150 mM NaCl, 1 mM EDTA) whereas the i-motif sequences (single and double strands) were analyzed in duplicate over a range of buffers from pH 2.2 to 10.8.

Preparation of the model single strand solution: the solution of the sample was prepared by mixing 400 µL of a stock solution of 20 mM Cacodylate buffer solution pH 7.0, 80 µL of 1.5 M NaCl solution, 10 µL of 80 mM EDTA solution, 25 µL of 64 µM ssODN and 285 µL of MilliQ® water.

Preparation of the model double strand solution: 400 µL of a stock solution of 20 mM Cacodylate buffer solution pH 7.0, 80 µL of 1.5 M NaCl solution, 10 µL of 80 mM EDTA solution, 25 µL of 64 µM **ODN 1**, 25 µL of 64 µM **ODN 2** and 260 µL of MilliQ® water.

Table S3. Composition of buffers pH 2.2 to 10.8.

pH	Na_2HPO_4 (mM)	Citric Acid (mM)	NaCl (mM)
2.2	4.0	98.0	142
3.1	41.0	79.5	67.7
4.3	77.1	61.5	-
5.3	74.9	35.3	-
6.2	74.9	21.9	-
7.2	75.0	8.0	-
8.2	74.9	1.1	-

pH	NaH_2PO_4 (mM)	NaOH (1M)	NaCl (mM)
9.00	127.8	Few drops to adjust the pH	22.1

pH	Na_2CO_3 (mM)	$NaHCO_3$ (mM)	NaCl (mM)
10.0	50	50	-
10.8	71	7.9	-

For the i-motif studies:

Preparation of the single strand solution: the solution of the sample was prepared by mixing 765 µL of buffer solution, 10 µL of 80 mM EDTA solution, 25 µL of 64 µM ssODN.

Preparation of the double strand solution: 740 µL of buffer solution, 10 µL of 80 mM EDTA solution, 25 µL of 64 µM **ODN 1**, 25 µL of 64 µM **ODN 2**.

4.2 Denaturation studies and melting temperatures

Melting curves were recorded in triplicate by following the temperature-dependence of the absorbance changes at 260 nm of the sample (2 µM concentration of each strand). Absorption spectra were recorded in a Peltier thermostated cell holder on a Cary 300 spectrophotometer (Varian). The path length of cell was 1 cm. The temperature range for denaturation measurement was 5–80 °C. Speed of heating was 0.3 °C/min. Cacodylate buffer pH 7.0 was used (10 mM cacodylate buffer, 150 mM NaCl). The melting curves were converted into a plot of α versus temperature, where α represents the fraction of single-strands in the duplex state. The melting temperatures were extracted from these curves after differentiation as described elsewhere.[10]

Fig S14. Melting temperature curves of the wild-type and labelled ODNs:

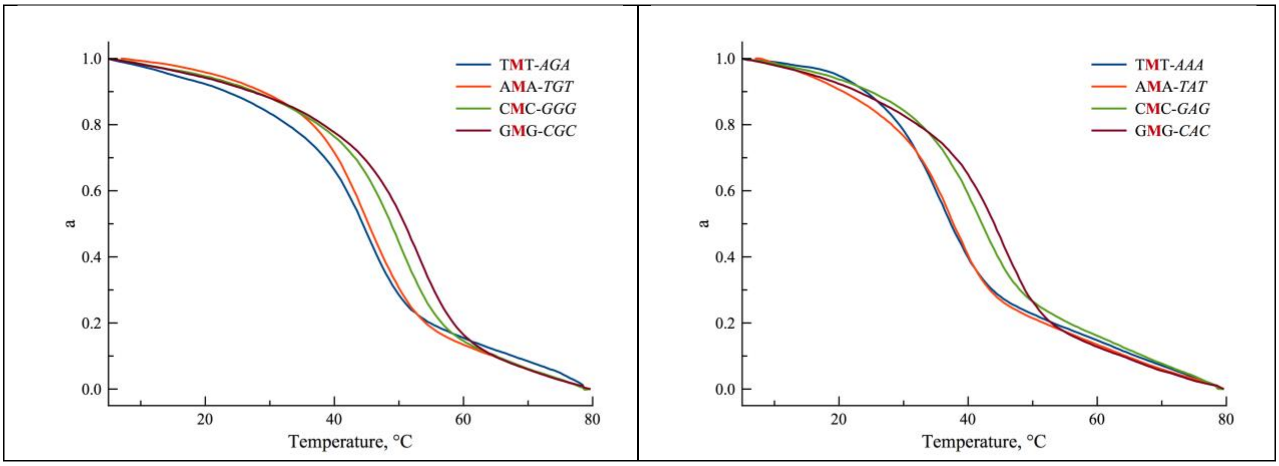

[10] Breslauer, K. J. *Methods Enzymol.* **1995**, *259*, 221−242.

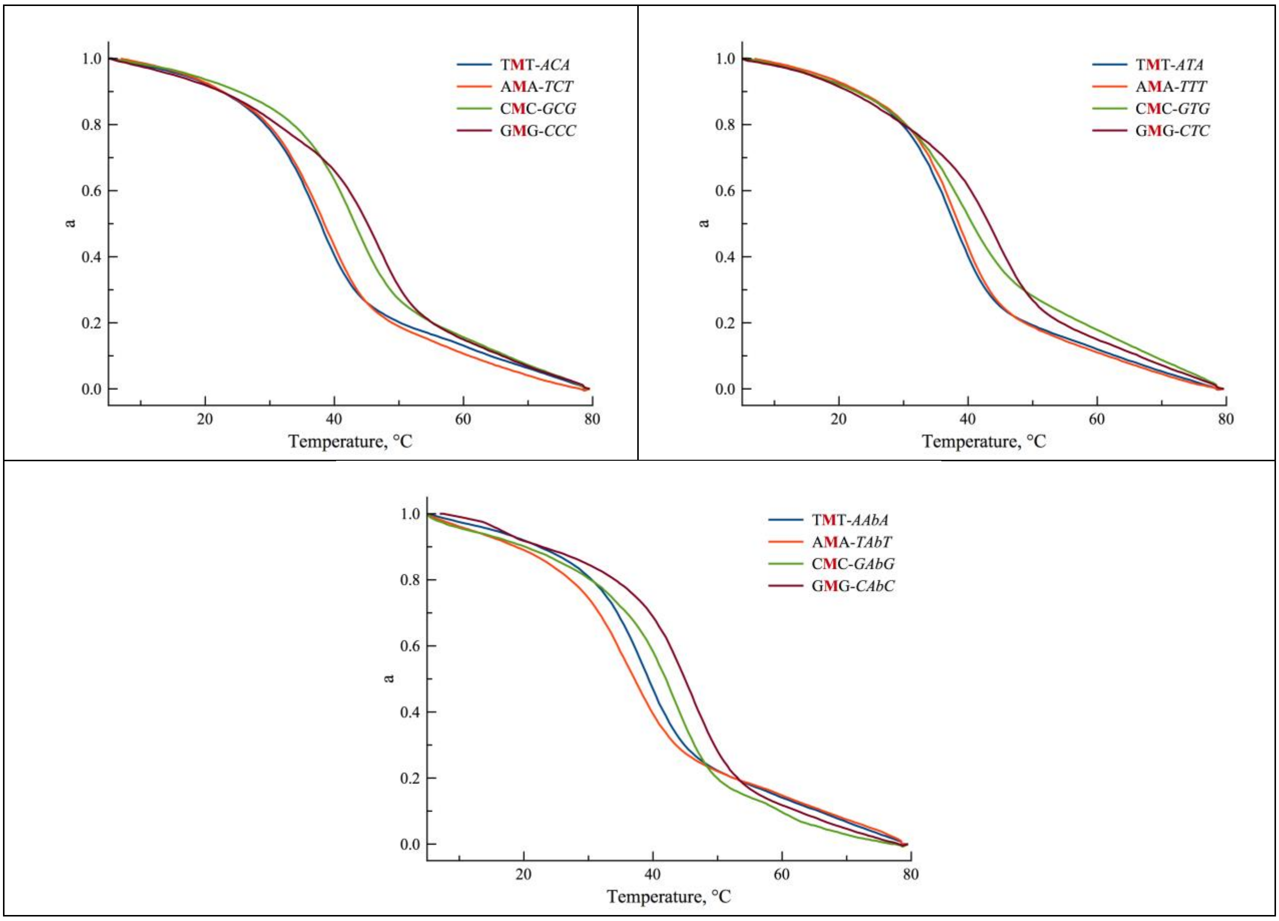

Fig S15. Melting temperature curves of wild-type and labelled i-motif sequences

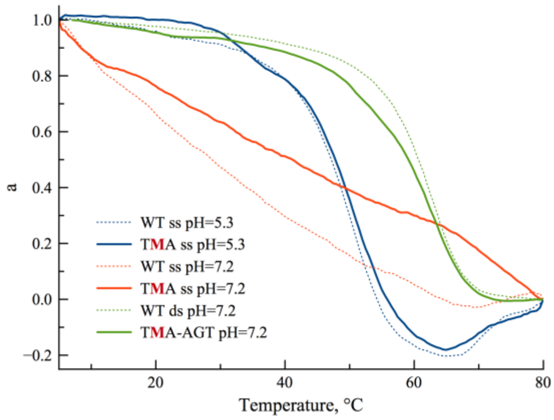

Table S4. Spectroscopic properties of 3HC-labeled ODNs.

Duplexes	T_m (°C)			λ_{Abs} (nm)	λ_{N*} (nm)	λ_{T*} (nm)	*Φ (%)*
	TCC	*Wild Type*[a]	ΔT_m (°C)				
T**M**T	-	-	-	401	505	569	**18**
T**M**T-*AGA*	45.1	49.7 [51.5]	-4.6	402	499	548	**17**
T**M**T-*ATA*	37.8	39.6 [40.8]	-1.8	406	505	565	**34**
T**M**T-*ACA*	38.0	35.9 [39.3]	+2.1	416	503	575	**31**
T**M**T-*AAA*	36.5	37.3 [40.3]	-0.8	406	506	572	**31**
T**M**T-*AAbA*	38.5	30.9	+7.6	413	504	572	**33**
A**M**A	-	-	-	408	503	569	**19**
A**M**A-*TGT*	45.0	50.3 [50.9]	-5.3	398	502	544	**13**

A**M**A-*TTT*	38.5	38.9 [40.2]	-0.4	405	512	570	**28**
A**M**A-*TCT*	38.5	35.4 [37.5]	+3.1	411	506	577	**26**
A**M**A-*TAT*	38.2	37.2 [40.2]	+1.0	406	510	573	**27**
A**M**A-*TAbT*	36.5	29.8	+6.7	409	511	570	**28**
C**M**C	-	-	-	404	508	569	**12**
C**M**C-*GGG*	50.0	54.2 [56.6]	-4.2	403	508	549	**6**
C**M**C-*GTG*	39.4	40.8 [43.9]	-1.4	409	510	559	**6**
C**M**C-*GCG*	42.7	40.8 [43.7]	+1.9	414	507	568	**3**
C**M**C-*GAG*	41.9	42.1 [44.2]	-0.2	406	514	562	**7**
C**M**C-*GAbG*	43.1	38.4	+4.7	411	508	566	**3**
G**M**G	-	-	-	403	502	572	**2**
G**M**G-*CGC*	52.8	59.1 [58.3]	-6.3	397	-	545	**< 1**
G**M**G-*CTC*	44.4	44.9 [46.2]	-0.5	405	504	571	**< 2**
G**M**G-*CCC*	46.4	42.9 [44.6]	+3.5	405	501	567	**< 2**
G**M**G-*CAC*	45.4	47.5 [45.6]	-2.1	408	509	567	**2**
G**M**G-*CAbC*	45.1	37.9	+7.2	405	502	570	**1**

[a] T_m of the corresponding duplexe formed from unmodified ODNs and its theoretical values given in square brackets.

4.3 Circular dichroism

Circular dichroism experiments were recorded at 20 °C on a Jasco J-810 spectropolarimeter. All the spectra were run in duplicate with 2 µM solution of the canonical model dsDNA and labelled model dsDNA (3HC (**M**) opposite **A**, **T**, **G**, **C** or **Ab**) in buffer pH 7.0 (10 mM cacodylate buffer, 150 mM NaCl, 1 mM EDTA) for the model ODNs and in a range of buffers from pH 2.2 to 10.8 for the i-motif sequences Two maxima were observed in CD spectra: one negative at 249 nm and the other positive at 282 nm.

Fig S16. Representative CD spectra of wild-type and labelled duplexes

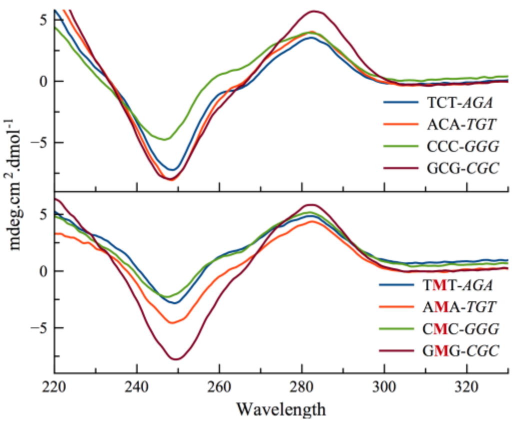

Fig S17. CD spectra of wild-type and labelled i-motif sequences (single strand: pH=5.3 and 7.2 and double strand: pH=7.2)

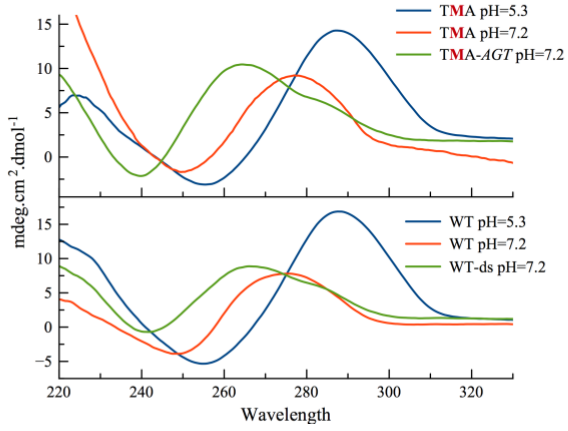

Fig S18. CD spectra of model AMA, wild-type and labelled i-motif sequences, and midpoint determination of i-motifs (pH=2.2 to 10.8)

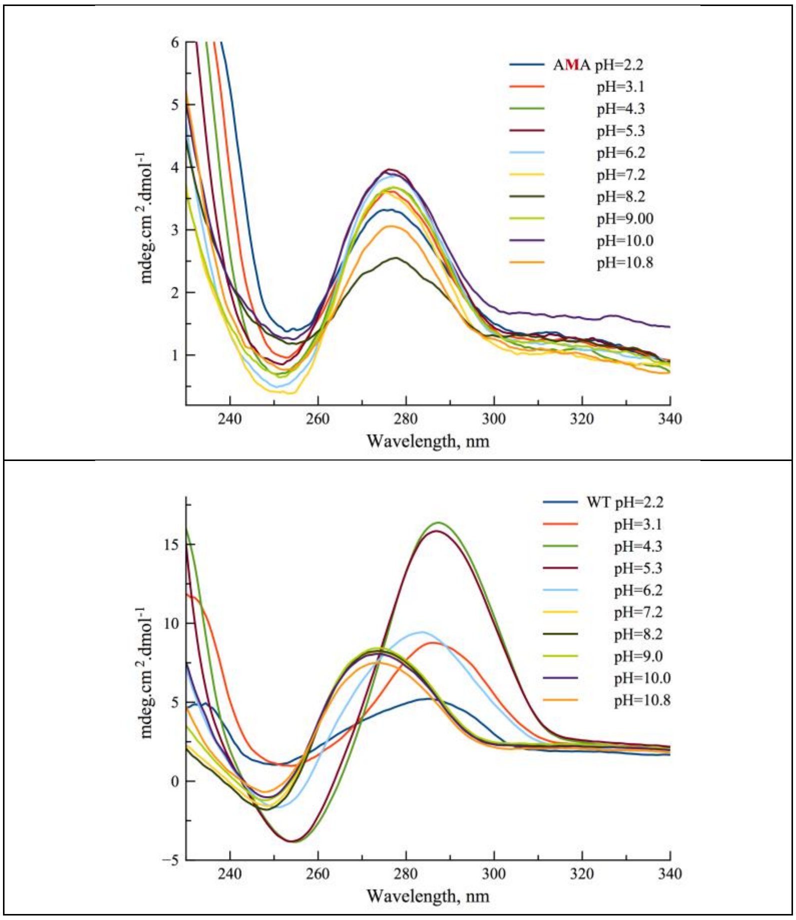

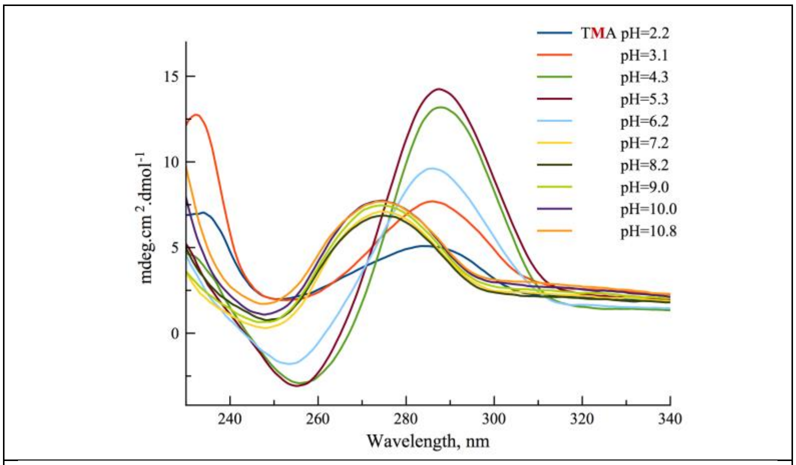

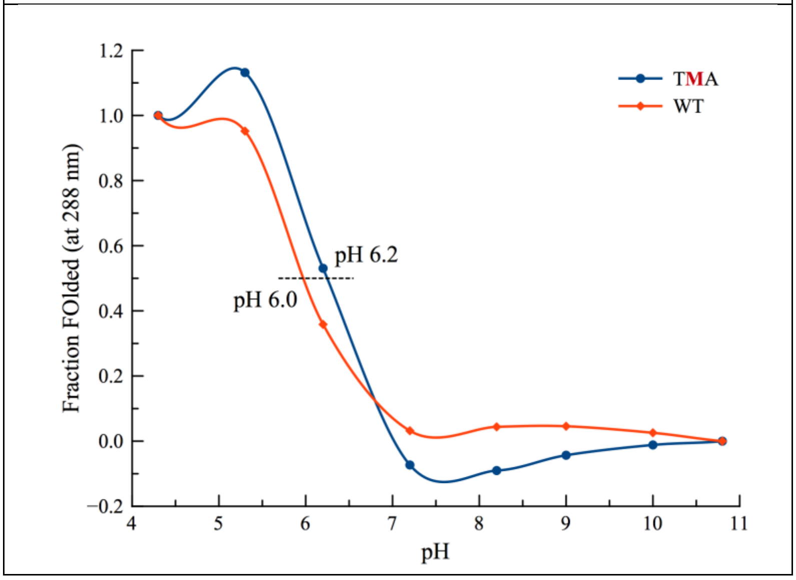

4.4 Absorption and Fluorescence spectra

The absorption and fluorescence experiments were conducted in duplicate in pH 7.0 phosphate buffer (20 mM sodium phosphate, 100 mM NaCl, 1 mM EDTA) for the model ODNs and in a range of buffers from pH 2.2 to 10.8 for the i-motif sequences. The absorption spectra were recorded on a Cary 300 Scan spectrophotometer (Varian) using 1 cm quartz cells at 20 °C. The fluorescence spectra were recorded on a FluoroMax 4.0 spectrofluorometer (Jobin Yvon, Horiba) by using excitation and emission slits of 2 nm and were corrected at excitation and emission. They were taken with absorbance of about 0.05 at 20 °C at the excitation wavelength mentioned in the corresponding experiments. The quantum yields were corrected according to the variation of the refractive index of the different solvents. Quantum yields were determined by using quinine sulfate (QS) in 0.1 M HCl solution (λ_{ex} = 350 nm, Φ□= 0.54)[11] and *p*-dimethylaminoflavone (dMAF) in EtOH (λ_{ex} = 404 nm, Φ= 0.27)[12] as standard references.

Fig S19. Absorbance spectra of model labelled single and double strands

[11] S. M. Ormson, R. G. Brown, F. Vollmer, W. Rettig, *J. Photochem. Photobiol. A* **1994**, *81*, 65-72.
[12] W. H. Melhuish, *J. Phys. Chem.* **1961**, *65*, 229-235.

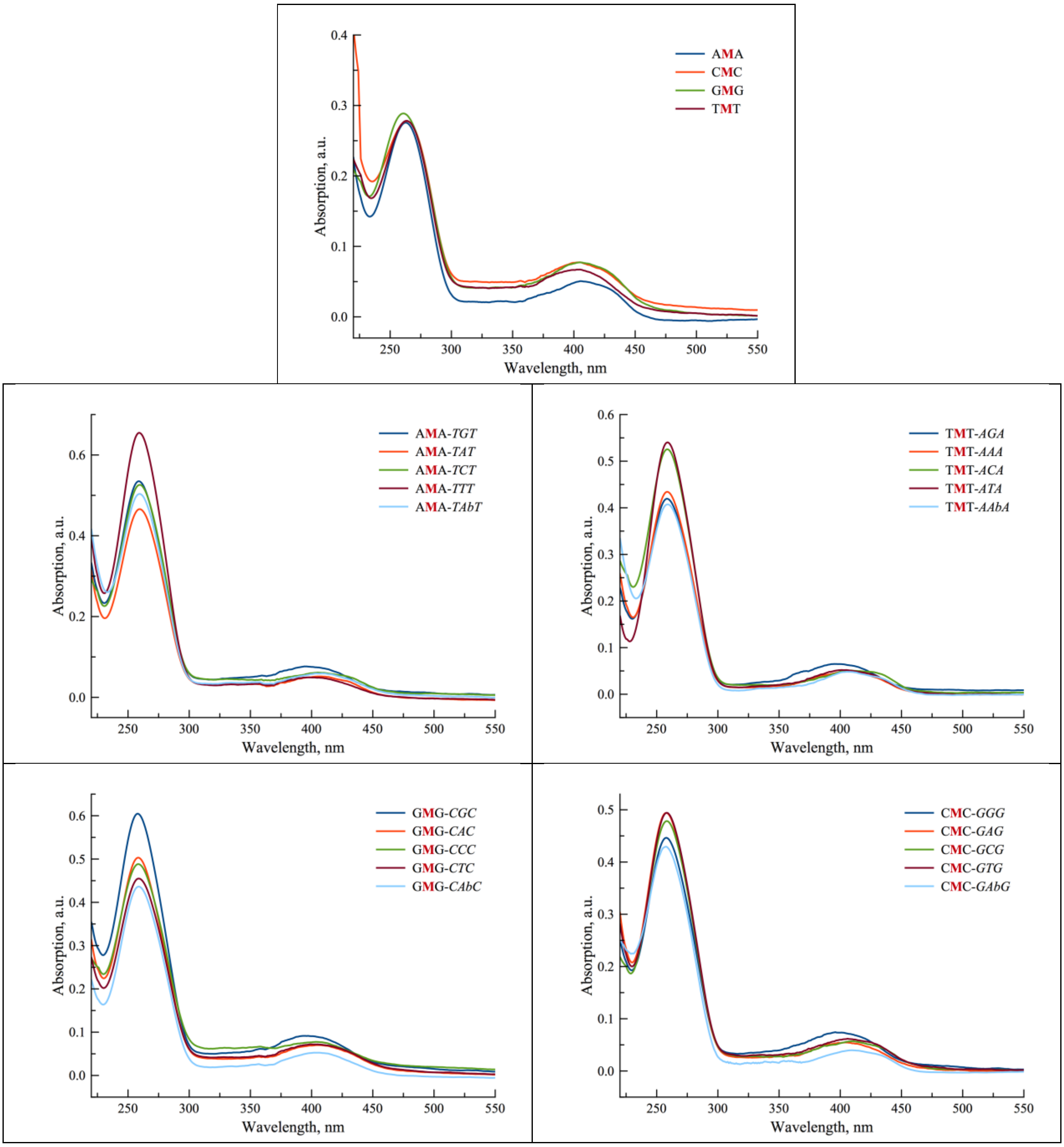

Fig S20. Absorbance spectra of single (pH 5.3 and 7.2) and double (pH 7.2) strand TMA

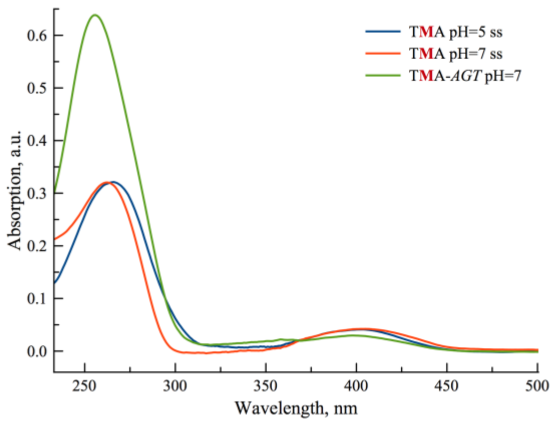

Fig S21. Absorbance spectra of AMA, TMA and wild-type sequences over the range pH=2.2 to 10.8

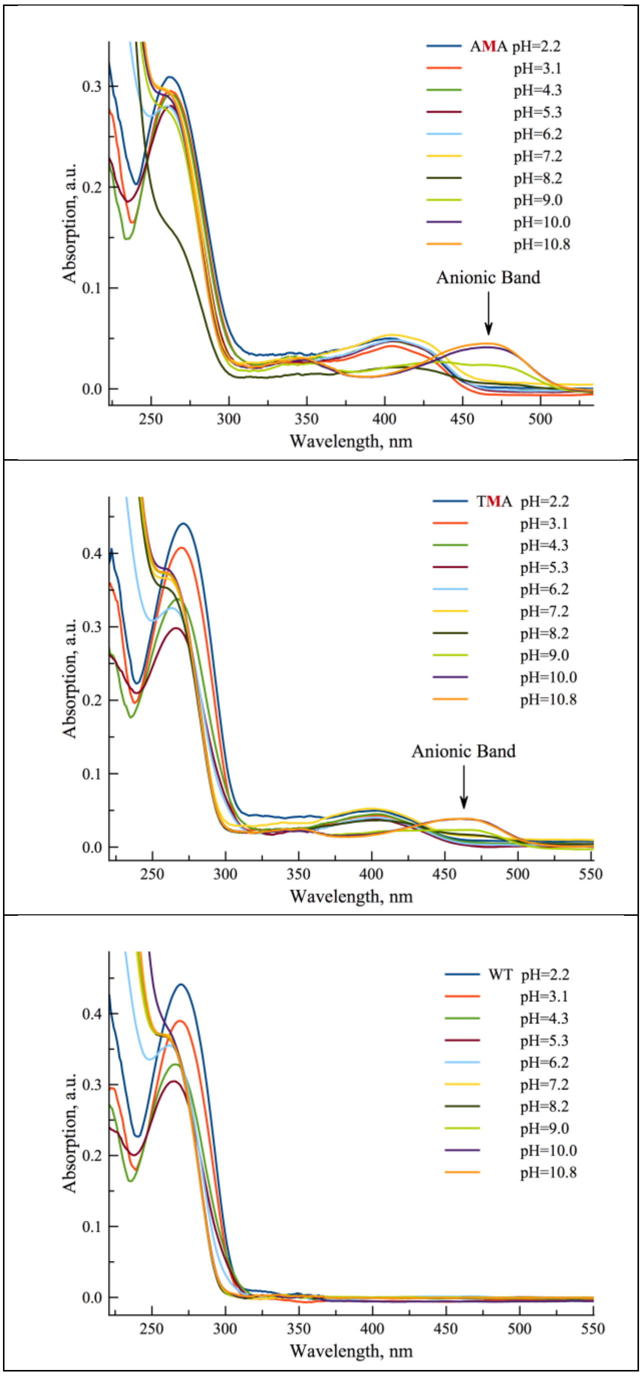

Fig S22. Determination of the pK$_A$ for the 3-OH of TCC incorporated into ss AMA (Blue) and TMA (green) sequences

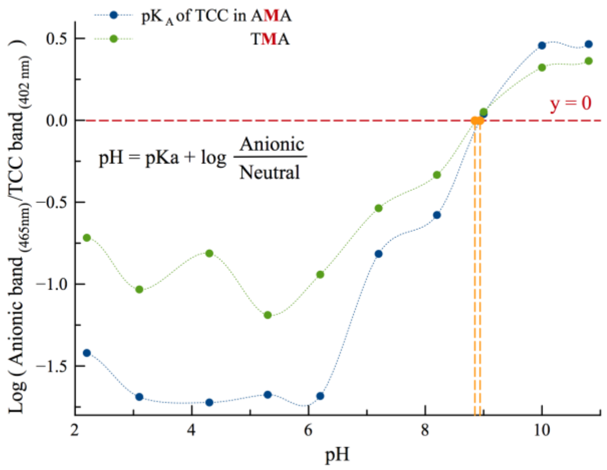

Sample	pK_A 3-O*H*
A**M**A	≈ 8.9
T**M**A	≈ 8.8

Fig S23. Fluorescence emission spectra of model labelled DNAs in different contexts of flanking bases

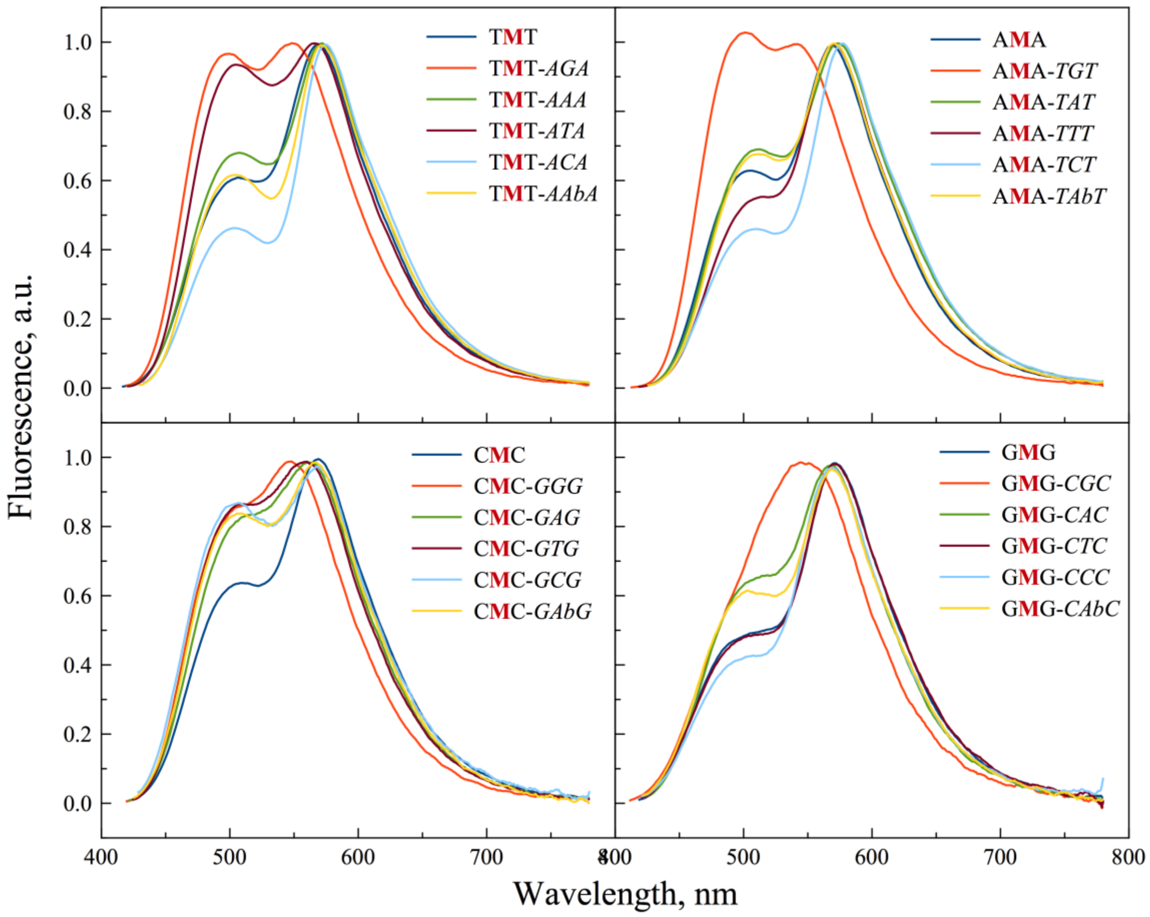

Fig S24. Midpoint determination of TMA i-motif (pH 4.5 ↔ pH 7.8)

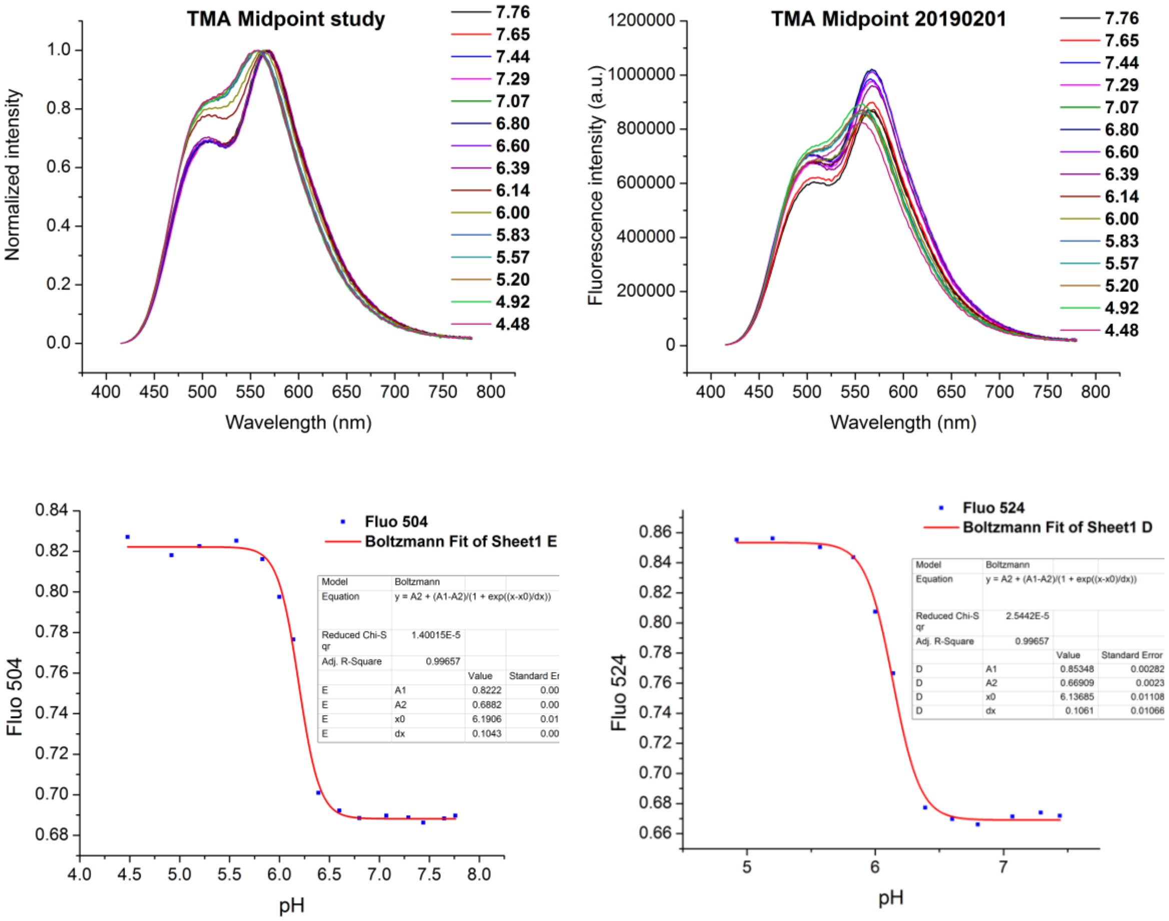

pH	7.76	7.65	7.44	7.29	7.07	6.8	6.6	6.39	6.14	6	5.83	5.57	5.2	4.92	4.48
QY (dMAF)	22.3	21.3	21.9	20.4	20.8	20.2	19.7	18.9	17.6	17.2	17.1	16.3	16.1	16.1	14.6

Fig S25. Determination of folding reversibility of TMA monitored through I_{N*}/I_{T*} variation (pH 5.3 ↔ pH 7.2)

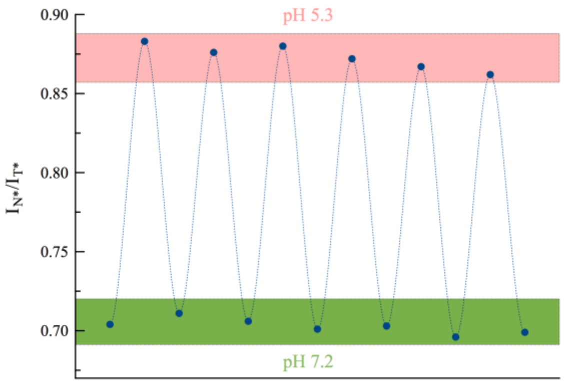

5. NMR spectra

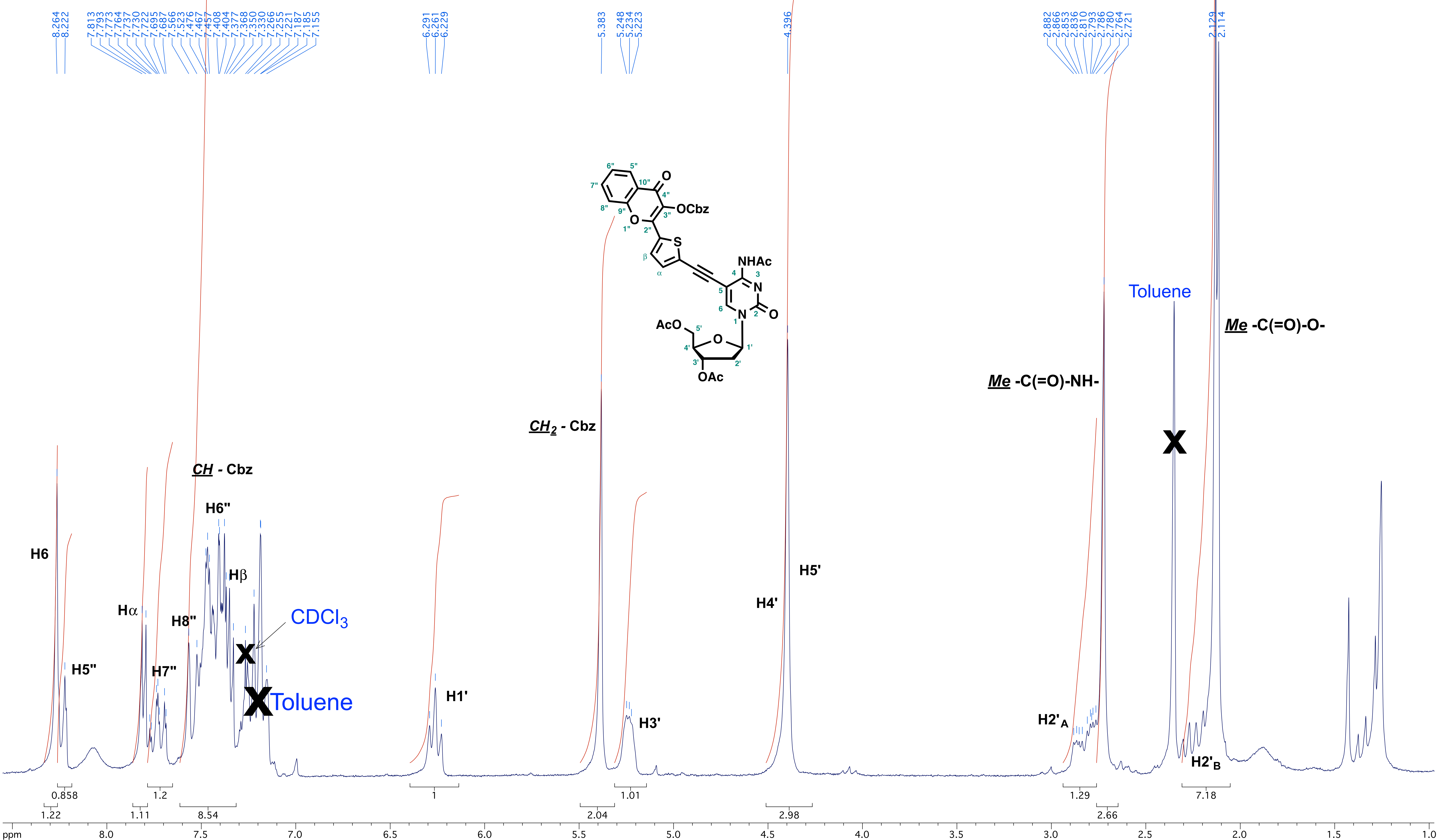

H6
H5"
Hα
H7"
H8"
CH - Cbz
H6"
Hβ
CDCl3
Toluene
H1'
CH2 - Cbz
H3'
H4'
H5'
Me -C(=O)-NH-
H2'A
Toluene
H2'B
Me -C(=O)-O-
AcO
OAc
NHAc
OCbz
ppm

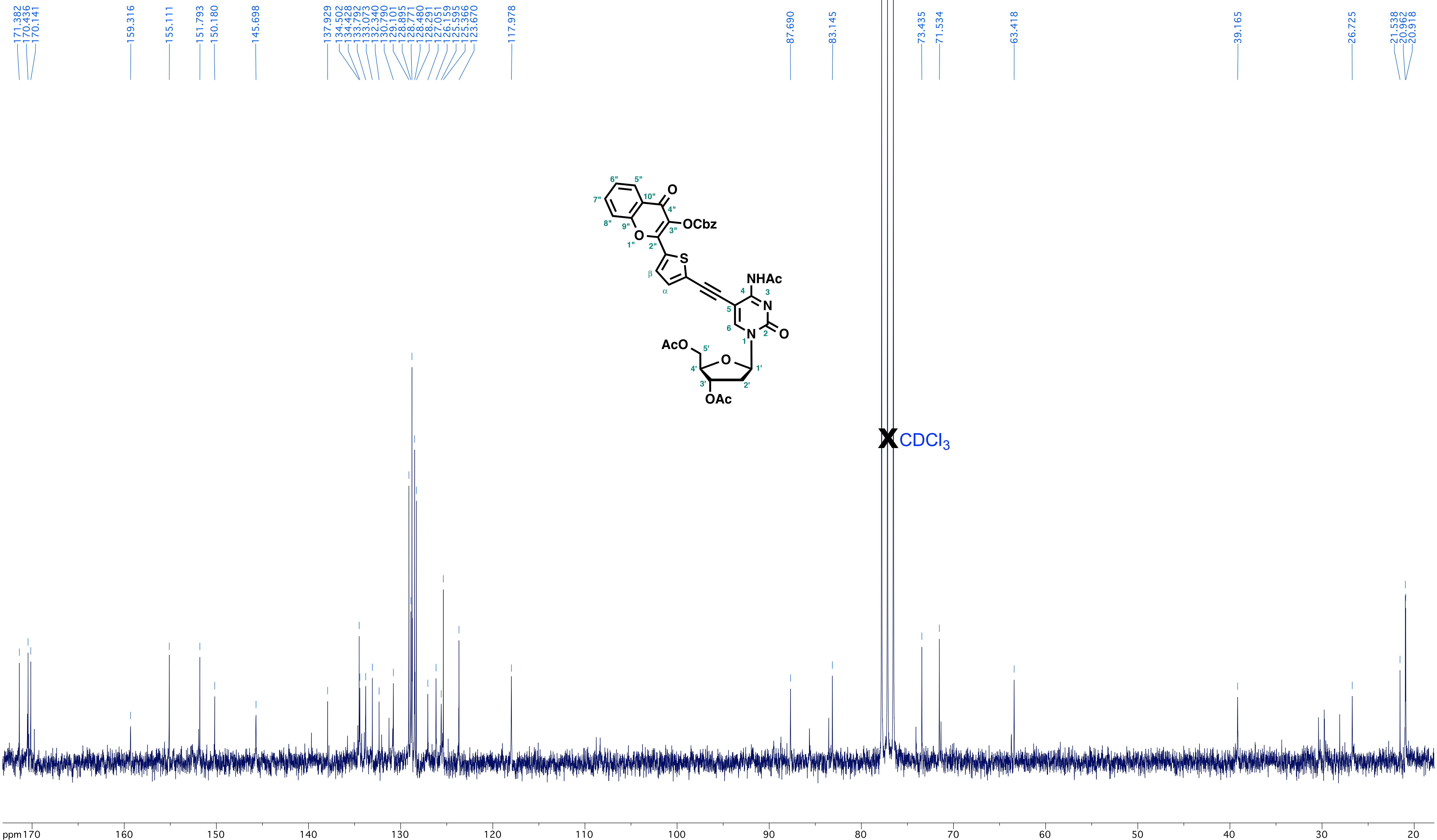

171.382
170.436
170.141
159.316
155.111
151.793
150.180
145.698
137.929
134.502
134.428
133.792
133.073
132.340
130.790
129.101
128.895
128.771
128.480
128.291
127.051
126.159
125.595
125.366
123.670
117.978
87.690
83.145
73.435
71.534
63.418
39.165
26.725
21.538
20.962
20.918
OCbz
NHAc
AcO
OAc
CDCl3
ppm 170
160
150
140
130
120
110
100
90
80
70
60
50
40
30
20

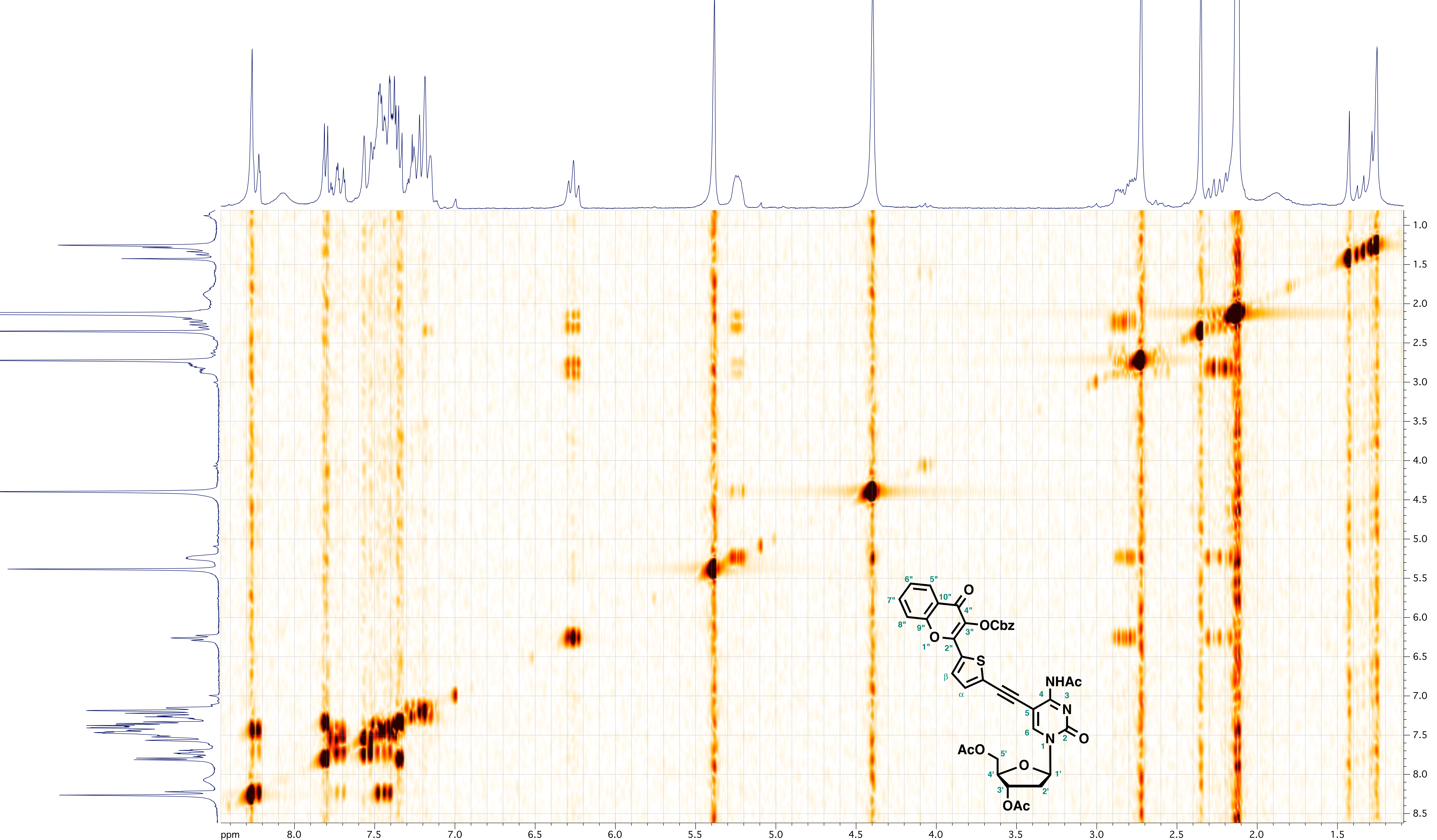
ppm
8.0
7.5
7.0
6.5
6.0
5.5
5.0
4.5
4.0
3.5
3.0
2.5
2.0
1.5
1.0
OCbz
NHAc
AcO
OAc

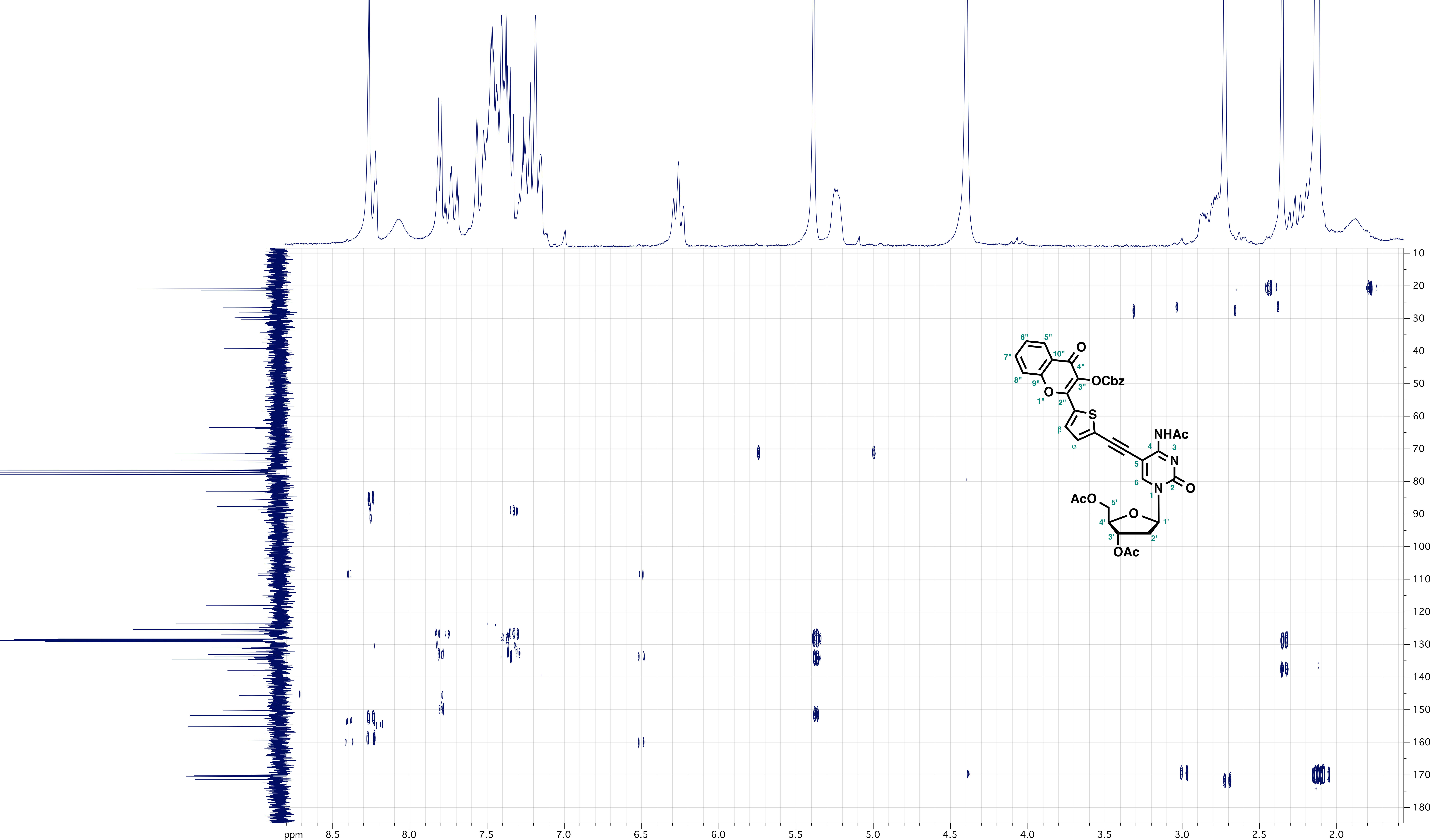

ppm
8.5
8.0
7.5
7.0
6.5
6.0
5.5
5.0
4.5
4.0
3.5
3.0
2.5
2.0
10
20
30
40
50
60
70
80
90
100
110
120
130
140
150
160
170
180
OCbz
NHAc
AcO
OAc
S
O
N

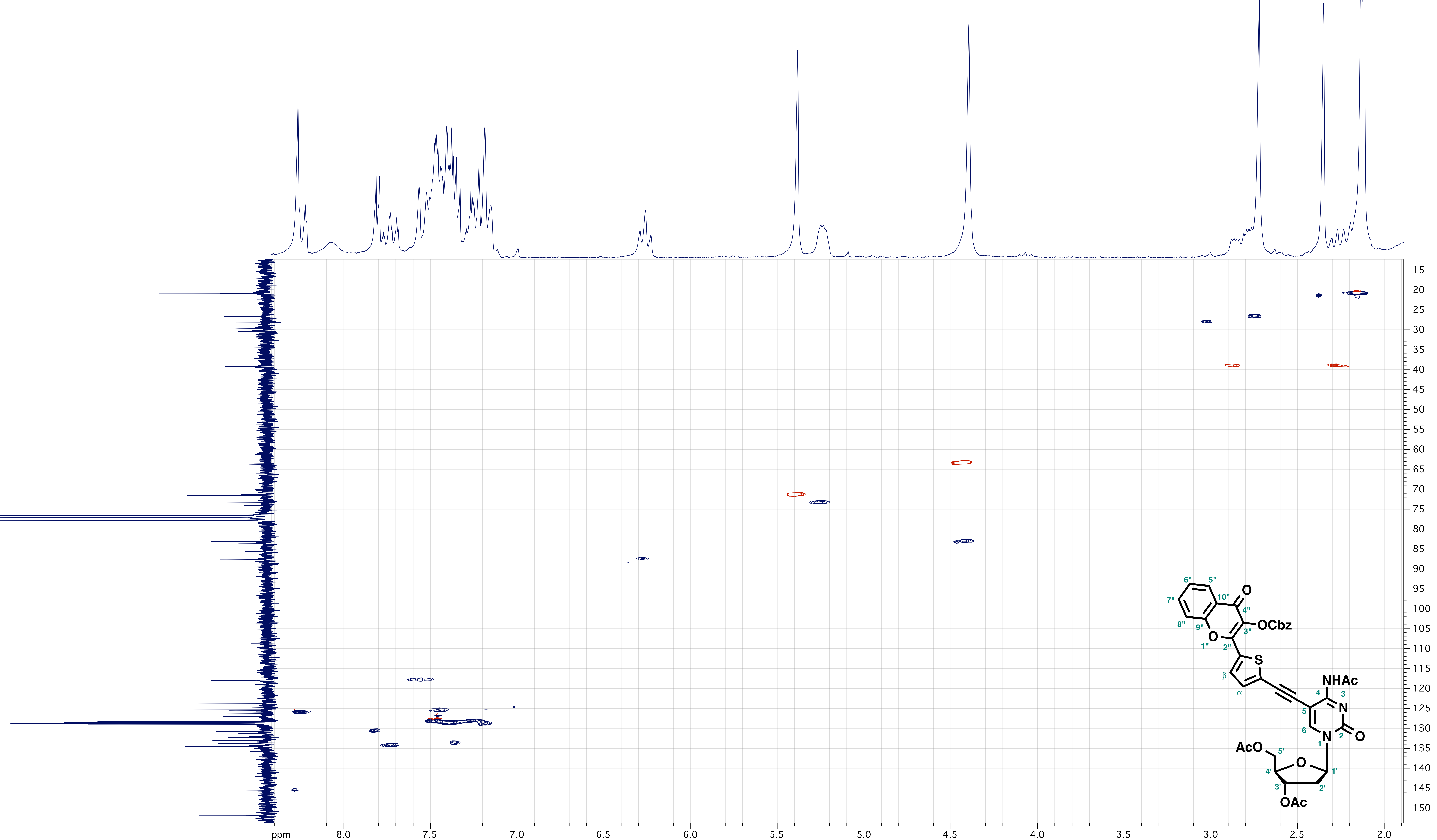
ppm
8.0
7.5
7.0
6.5
6.0
5.5
5.0
4.5
4.0
3.5
3.0
2.5
2.0
15
20
25
30
35
40
45
50
55
60
65
70
75
80
85
90
95
100
105
110
115
120
125
130
135
140
145
150
O
OCbz
S
NHAc
N
O
N
AcO
O
OAc

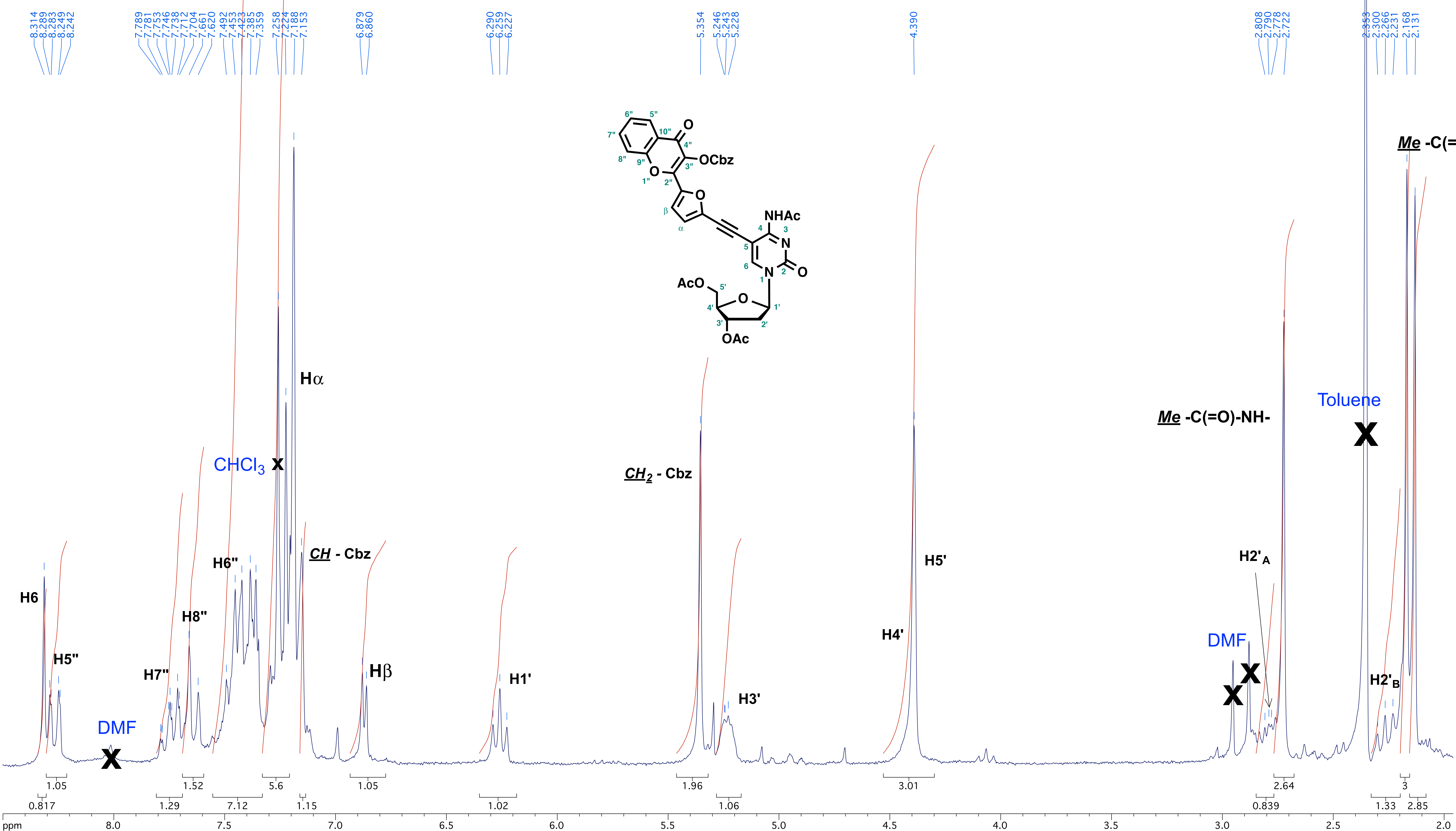

8.314
8.289
8.283
8.249
8.242
7.789
7.781
7.753
7.746
7.738
7.712
7.704
7.661
7.620
7.492
7.453
7.423
7.385
7.359
7.258
7.224
7.188
7.153
6.879
6.860
6.290
6.259
6.227
5.354
5.246
5.243
5.228
4.390
2.808
2.790
2.778
2.722
2.353
2.300
2.266
2.231
2.168
2.131
6"
5"
7"
10"
O
4"
OCbz
8"
9"
3"
O
1"
2"
O
β
α
NHAc
4
3
N
5
6
N
O
2
1
AcO
5'
O
1'
4'
3'
2'
OAc
Hα
CHCl3
CH2 - Cbz
CH - Cbz
H6
H5"
DMF
H7"
H8"
H6"
Hβ
H1'
H3'
H4'
H5'
DMF
H2'A
Me -C(=O)-NH-
Toluene
H2'B
Me -C(=
1.05
0.817
1.29
1.52
7.12
5.6
1.15
1.05
1.02
1.96
1.06
3.01
0.839
2.64
1.33
3
2.85
ppm
8.0
7.5
7.0
6.5
6.0
5.5
5.0
4.5
4.0
3.5
3.0
2.5
2.0

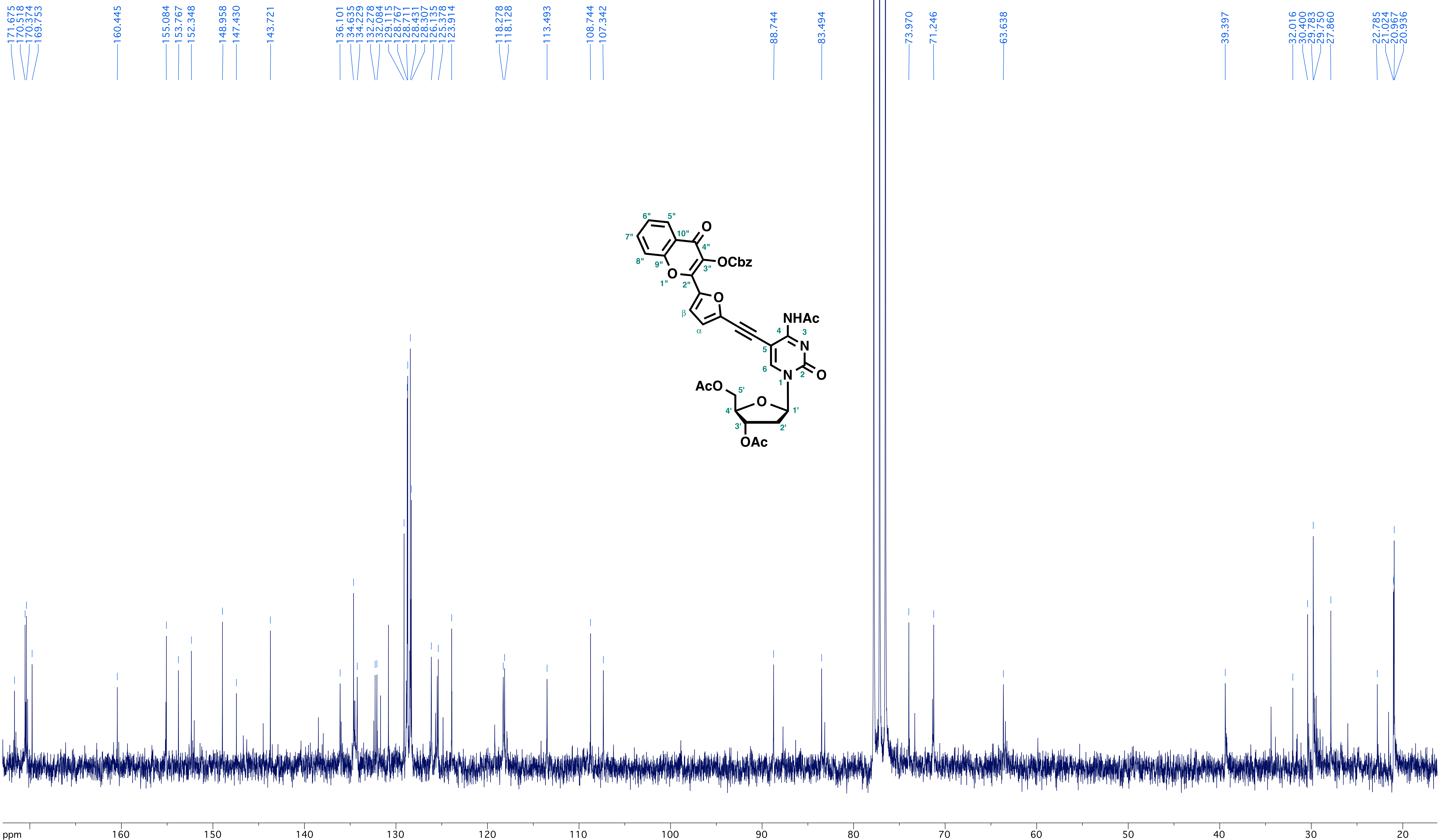

171.675
170.518
170.374
169.753
160.445
155.084
153.767
152.348
148.958
147.430
143.721
136.101
134.635
134.229
132.278
132.084
129.115
128.767
128.711
128.431
128.307
126.135
125.378
123.914
118.278
118.128
113.493
108.744
107.342
88.744
83.494
73.970
71.246
63.638
39.397
32.016
30.400
29.783
29.750
27.860
22.785
21.024
20.967
20.936
OCbz
NHAc
AcO
OAc
ppm
160
150
140
130
120
110
100
90
80
70
60
50
40
30
20

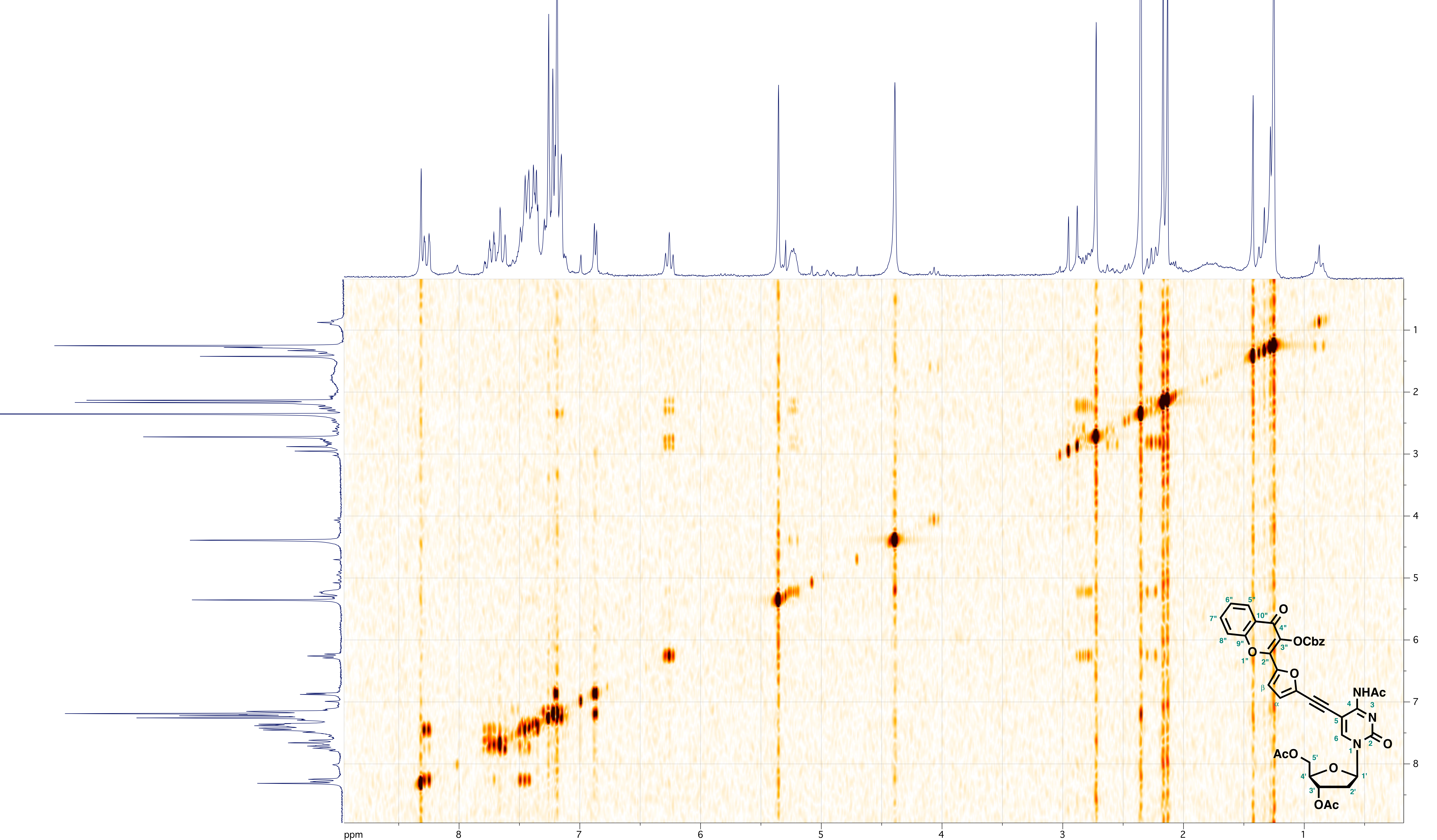

ppm
8
7
6
5
4
3
2
1
OCbz
NHAc
AcO
OAc
O
N
1"
2"
3"
4"
5"
6"
7"
8"
9"
10"
β
α
1
2
3
4
5
6
1'
2'
3'
4'
5'

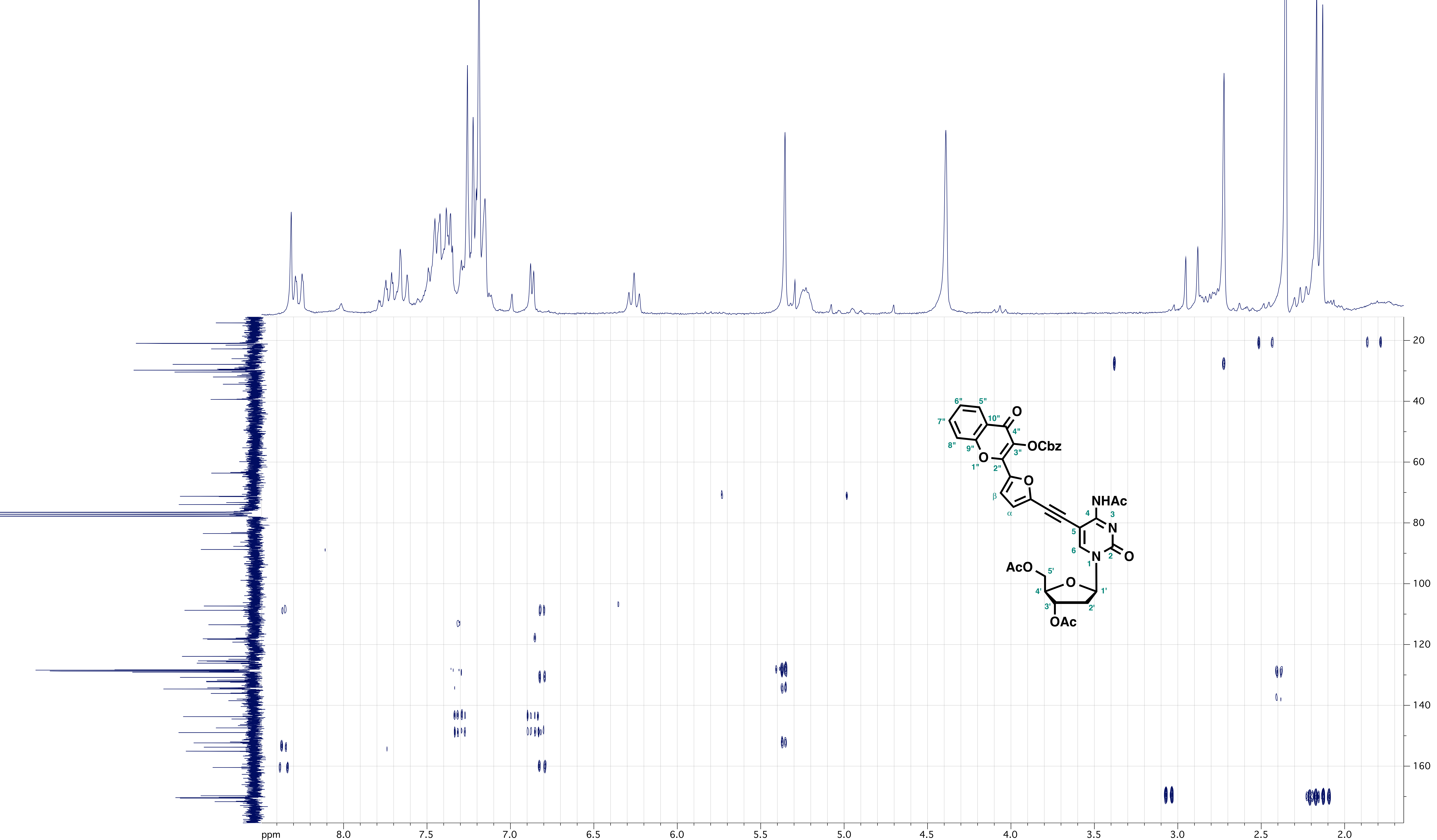

ppm
8.0
7.5
7.0
6.5
6.0
5.5
5.0
4.5
4.0
3.5
3.0
2.5
2.0
20
40
60
80
100
120
140
160
O
OCbz
NHAc
N
AcO
OAc
6"
5"
10"
7"
4"
8"
9"
3"
1"
2"
β
α
4
3
5
6
1
2
5'
4'
1'
3'
2'

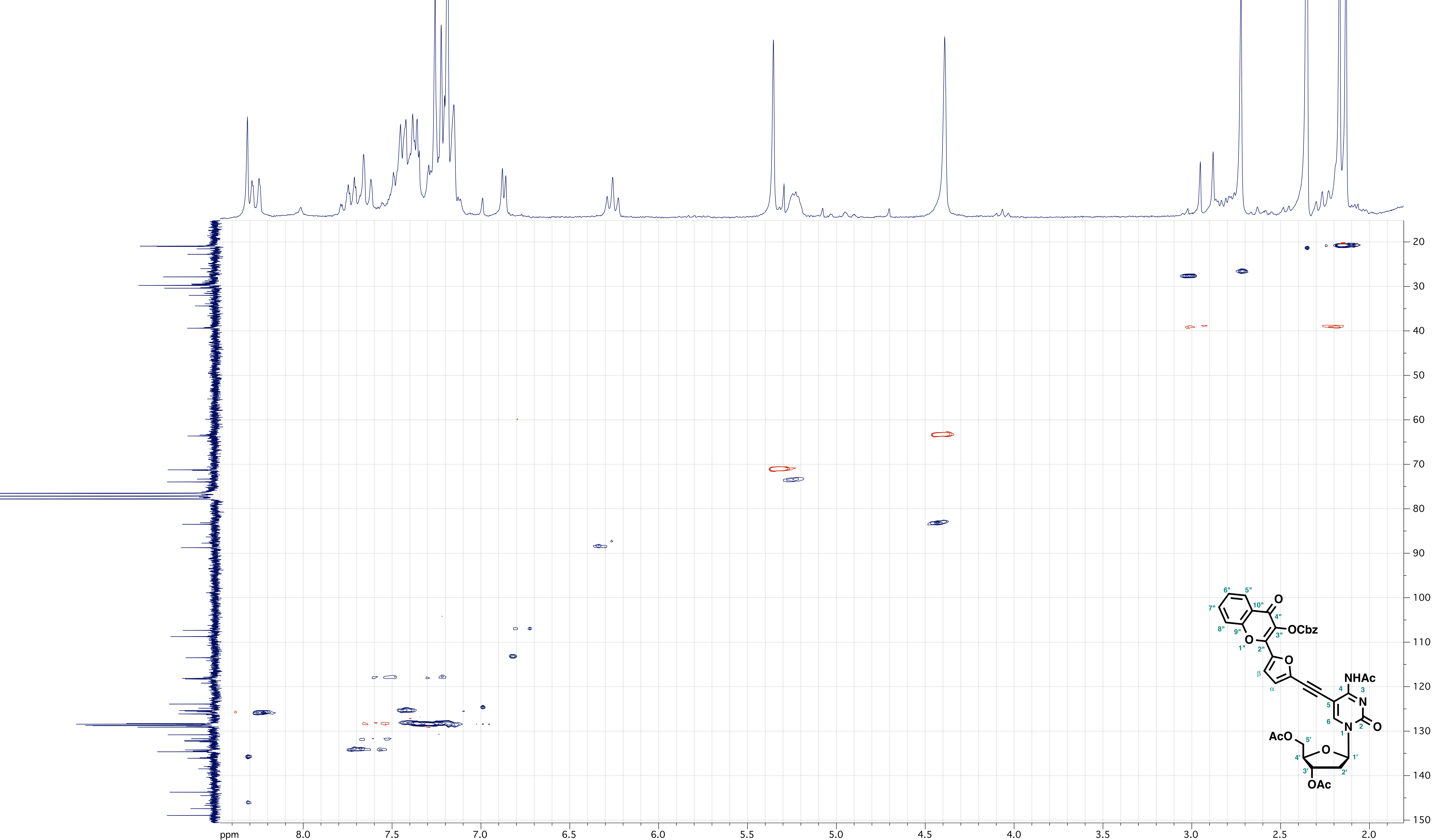
ppm
8.0
7.5
7.0
6.5
6.0
5.5
5.0
4.5
4.0
3.5
3.0
2.5
2.0
20
30
40
50
60
70
80
90
100
110
120
130
140
150
OCbz
NHAc
AcO
OAc

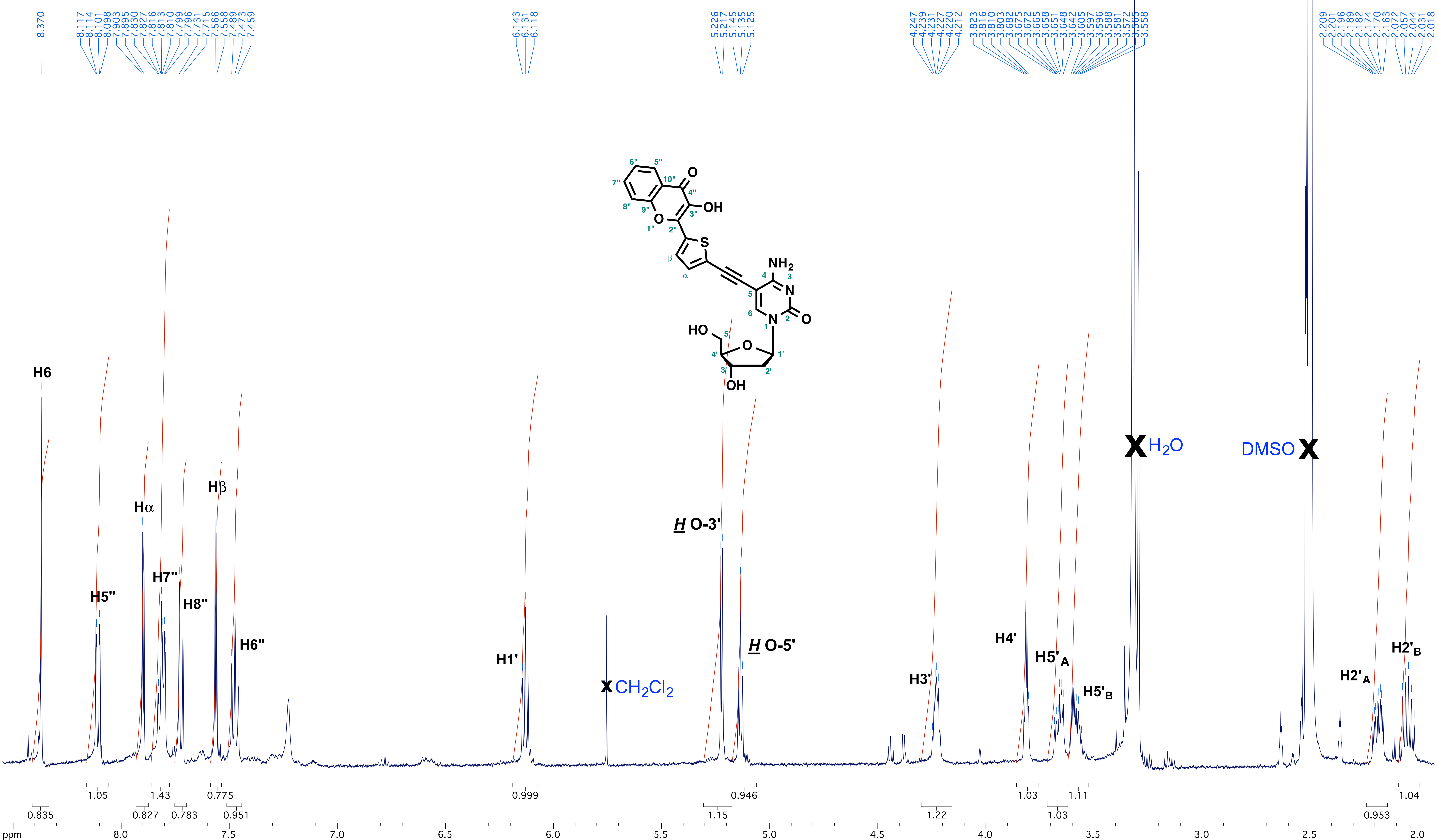
8.370
8.117
8.114
8.101
8.098
7.903
7.895
7.830
7.827
7.816
7.813
7.810
7.799
7.796
7.731
7.715
7.566
7.558
7.489
7.473
7.459
6.143
6.131
6.118
5.226
5.217
5.145
5.135
5.125
4.247
4.239
4.231
4.227
4.220
4.212
3.823
3.816
3.810
3.803
3.682
3.675
3.672
3.665
3.658
3.651
3.648
3.642
3.605
3.597
3.596
3.588
3.581
3.572
3.565
3.558
2.209
2.201
2.196
2.189
2.182
2.174
2.170
2.163
2.057
2.044
2.031
2.018
H6
H5"
Hα
H7"
H8"
Hβ
H6"
H1'
CH2Cl2
H O-3'
H O-5'
H3'
H4'
H5'A
H5'B
H2O
DMSO
H2'A
H2'B
NH2
OH
HO
O
S
0.835
1.05
0.827
1.43
0.783
0.775
0.951
0.999
1.15
0.946
1.22
1.03
1.03
1.11
0.953
1.04
ppm
8.0
7.5
7.0
6.5
6.0
5.5
5.0
4.5
4.0
3.5
3.0
2.5
2.0

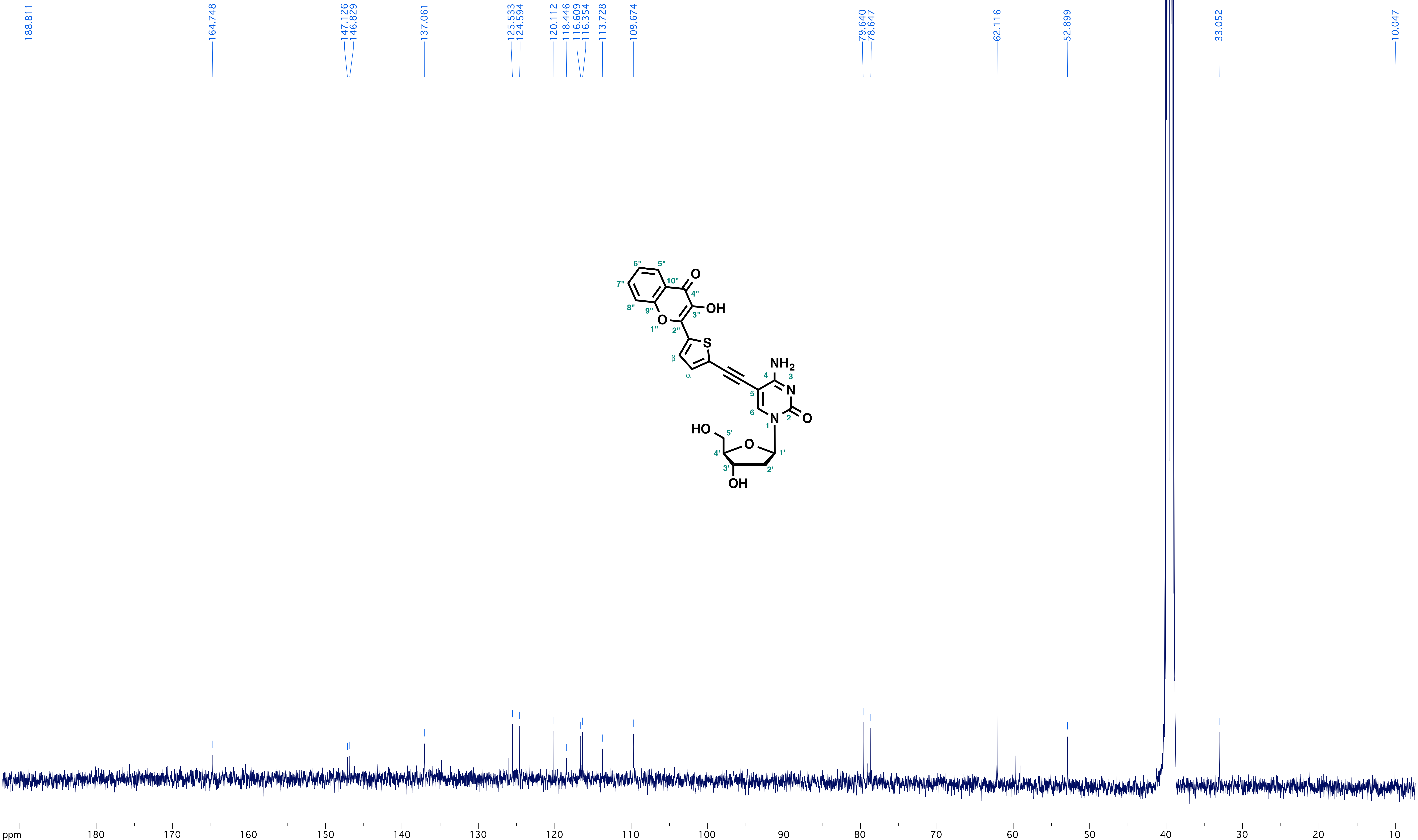
188.811
164.748
147.126
146.829
137.061
125.533
124.594
120.112
118.446
116.609
116.354
113.728
109.674
79.640
78.647
62.116
52.899
33.052
10.047
O
OH
O
S
NH2
N
O
N
HO
O
OH
5"
6"
7"
8"
9"
10"
4"
3"
2"
1"
β
α
4
3
5
6
2
1
5'
4'
3'
2'
1'
ppm
180
170
160
150
140
130
120
110
100
90
80
70
60
50
40
30
20
10

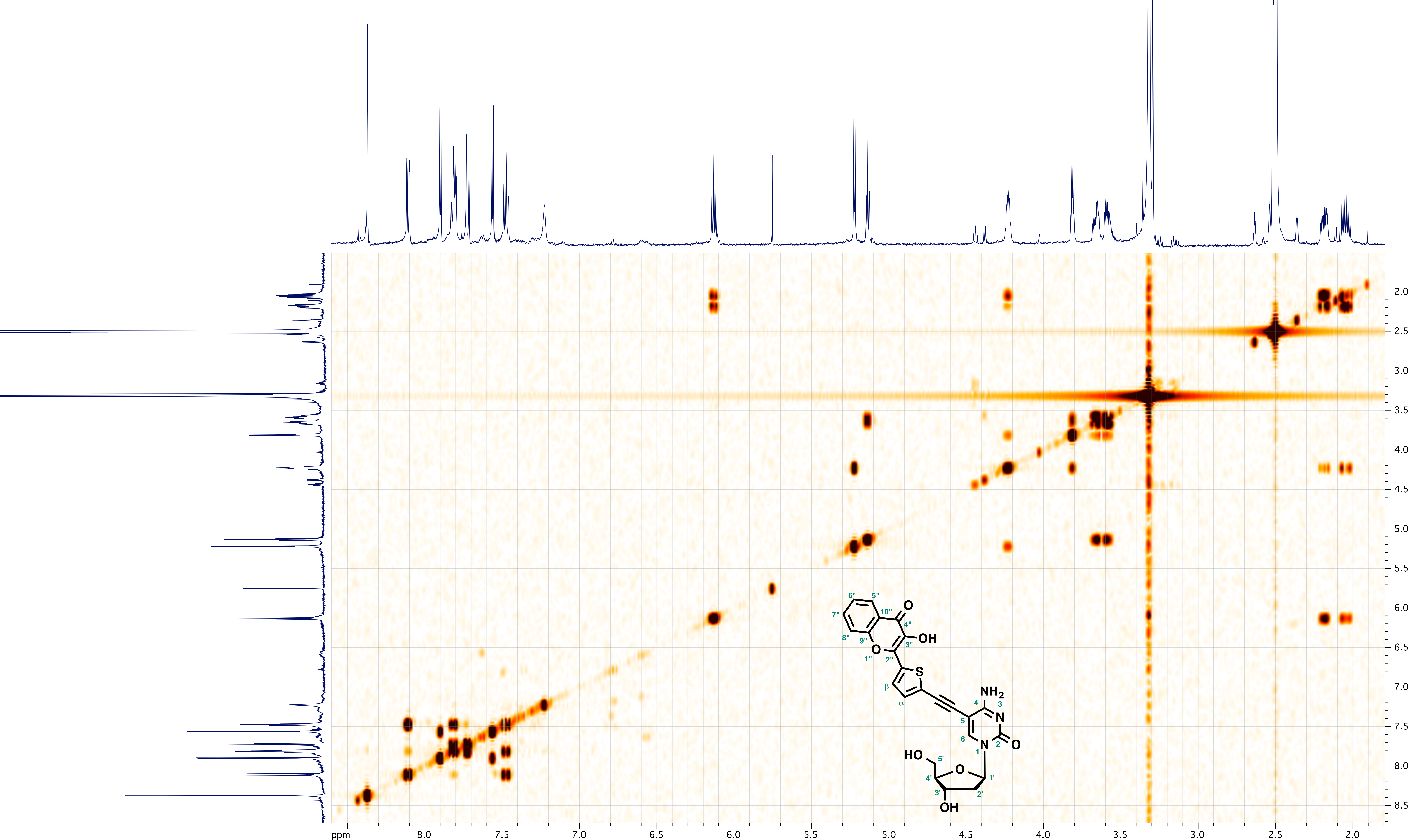
ppm
8.0
7.5
7.0
6.5
6.0
5.5
5.0
4.5
4.0
3.5
3.0
2.5
2.0
8.5
NH2
OH
HO
OH
S
O
N
6"
5"
7"
8"
9"
10"
4"
3"
1"
2"
β
α
4
3
5
6
1
2
5'
4'
3'
2'
1'

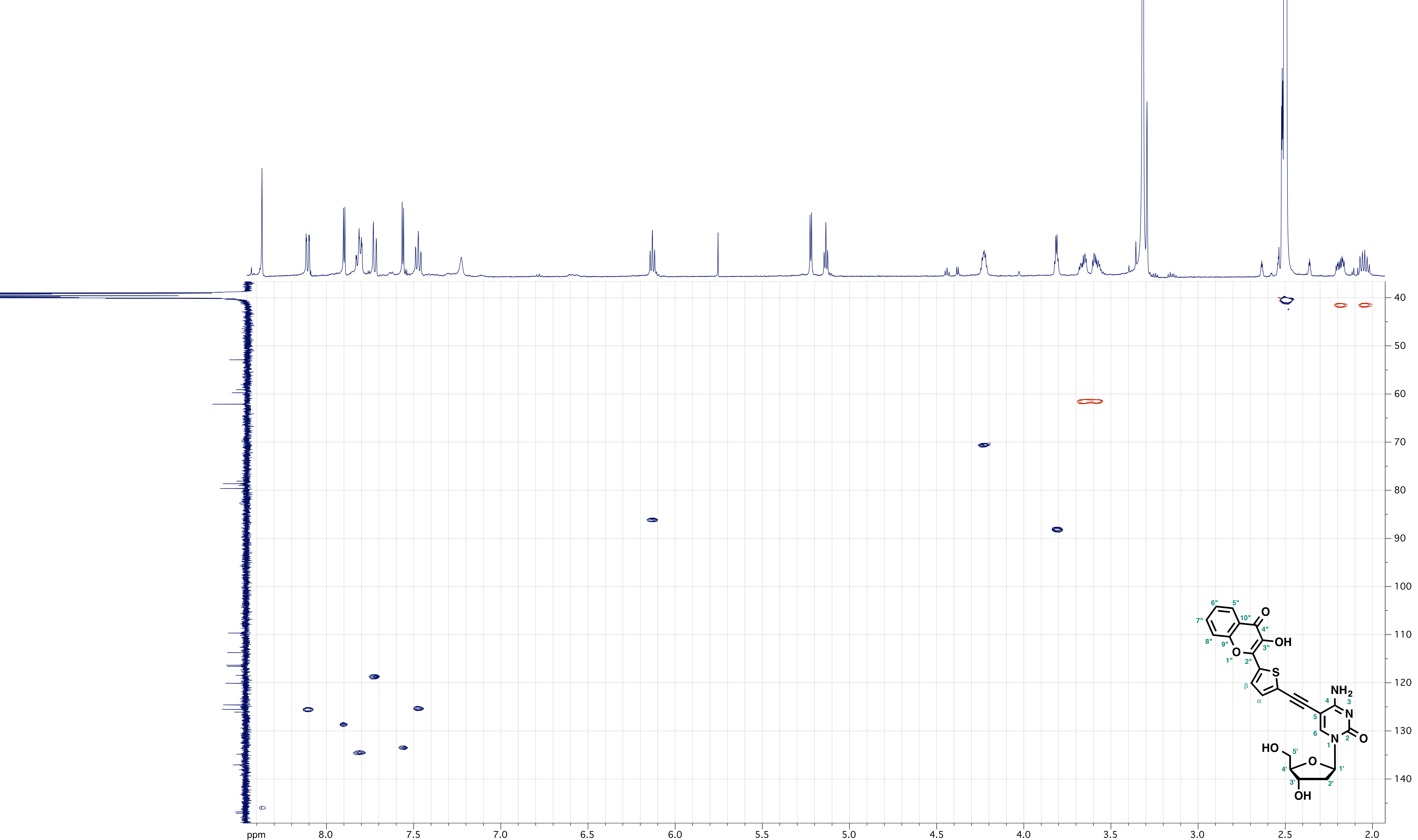
ppm
8.0
7.5
7.0
6.5
6.0
5.5
5.0
4.5
4.0
3.5
3.0
2.5
2.0
40
50
60
70
80
90
100
110
120
130
140
O
OH
O
S
NH2
N
O
N
HO
O
OH
6"
5"
7"
10"
4"
8"
9"
3"
1"
2"
β
α
4
3
5
6
2
1
5'
4'
1'
3'
2'

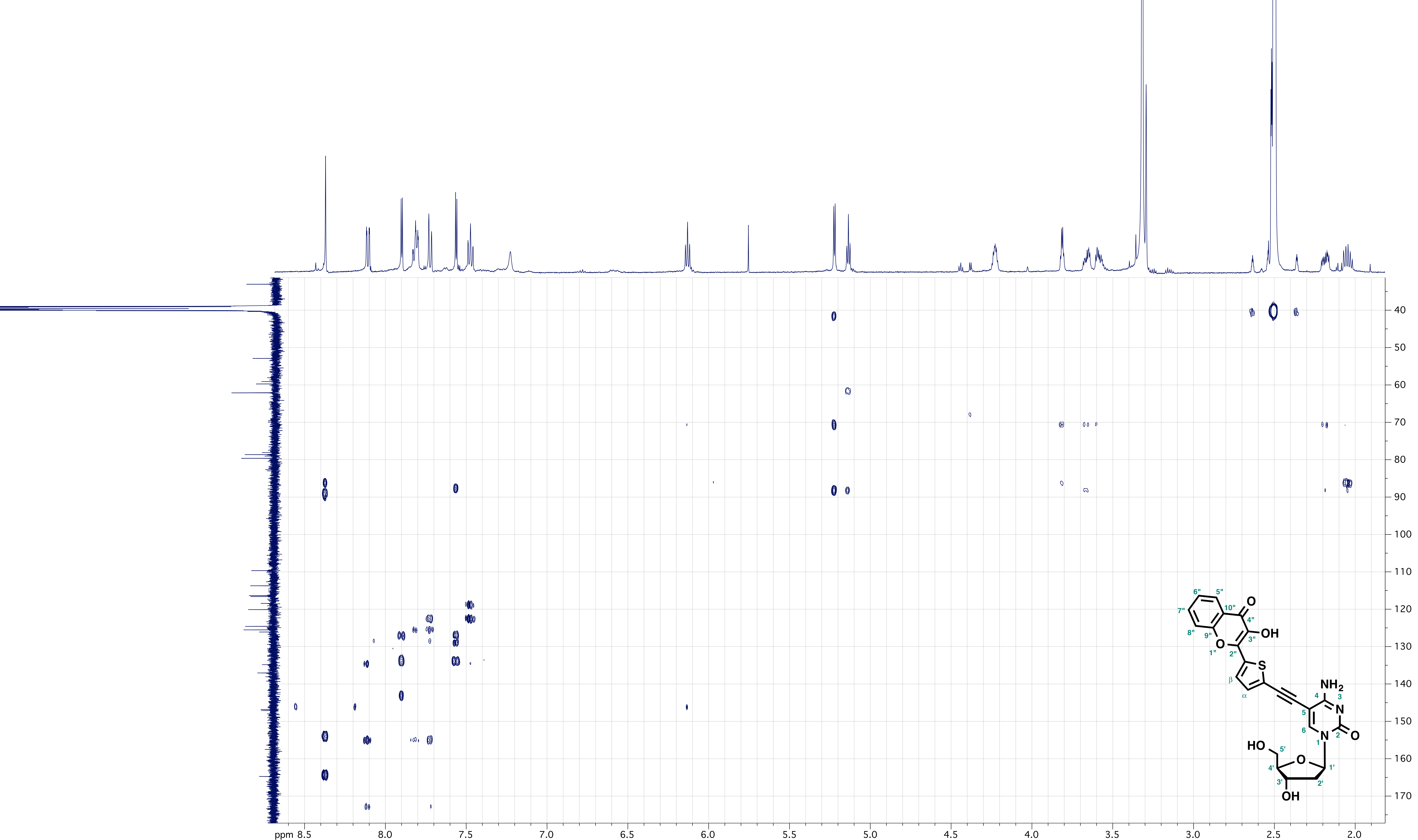

ppm 8.5
8.0
7.5
7.0
6.5
6.0
5.5
5.0
4.5
4.0
3.5
3.0
2.5
2.0
40
50
60
70
80
90
100
110
120
130
140
150
160
170
O
OH
S
NH_2
N
O
HO
O
OH

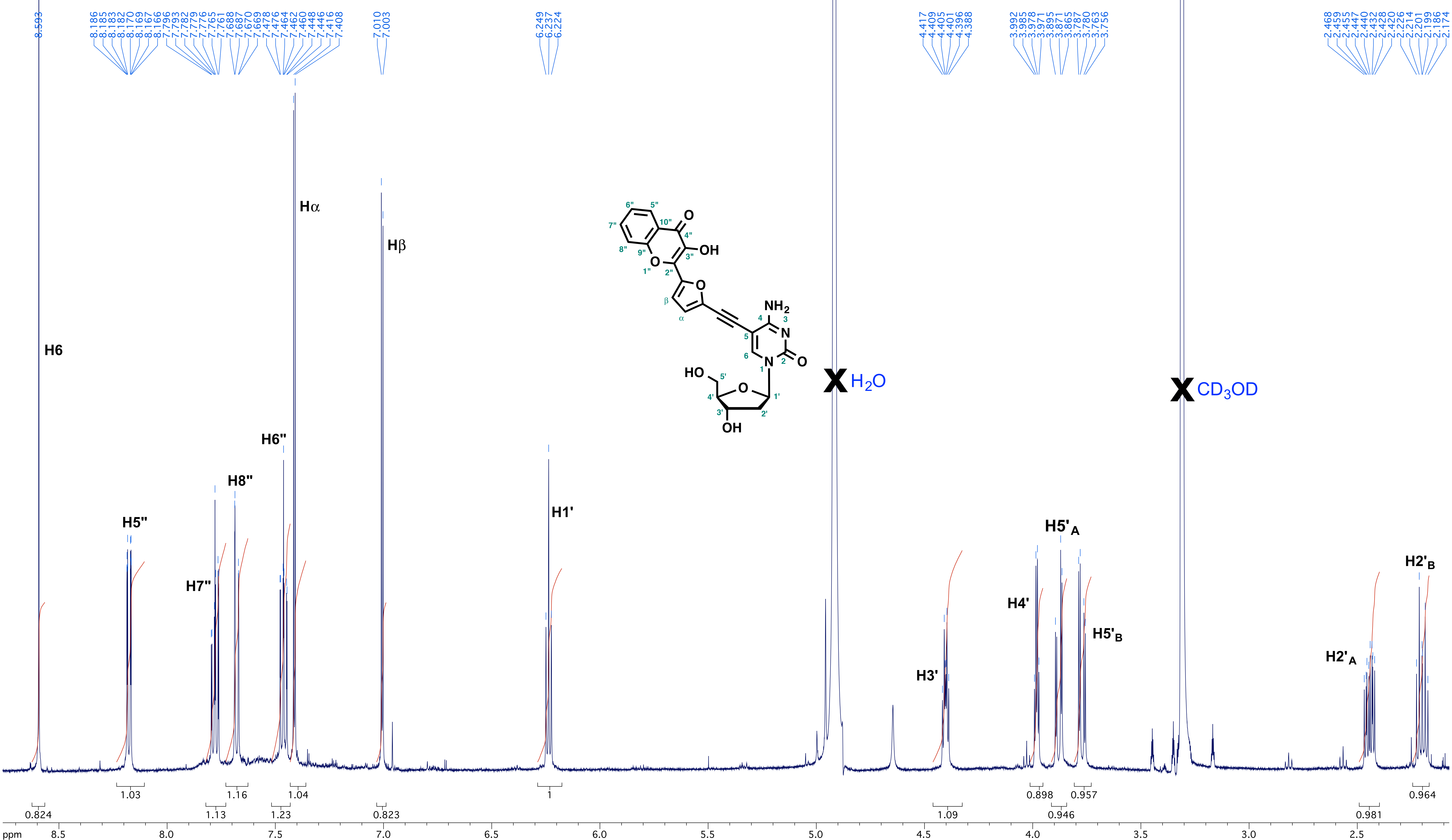

8.593
8.186 8.185 8.183 8.182 8.170 8.169 8.167 8.166 7.796 7.793 7.782 7.779 7.776 7.765 7.761 7.688 7.687 7.670 7.669 7.478 7.476 7.464 7.462 7.460 7.448 7.446 7.416 7.408
7.010 7.003
6.249 6.237 6.224
4.417 4.409 4.405 4.401 4.396 4.388
3.992 3.985 3.978 3.971 3.895 3.871 3.865 3.787 3.780 3.763 3.756
2.468 2.459 2.455 2.447 2.440 2.432 2.428 2.420 2.226 2.214 2.201 2.199 2.186 2.174
H6
H5"
H7"
H8"
H6"
Hα
Hβ
H1'
H3'
H4'
H5'A
H5'B
H2'A
H2'B
X H2O
X CD3OD
6" 5" 7" 10" O 4" 8" 9" O 3" OH 1" 2" O β α NH2 4 3 5 N 6 2 O 1 N HO 5' O 4' 1' 3' 2' OH
0.824 1.03 1.13 1.16 1.23 1.04 0.823 1 1.09 0.898 0.946 0.957 0.981 0.964
ppm 8.5 8.0 7.5 7.0 6.5 6.0 5.5 5.0 4.5 4.0 3.5 3.0 2.5

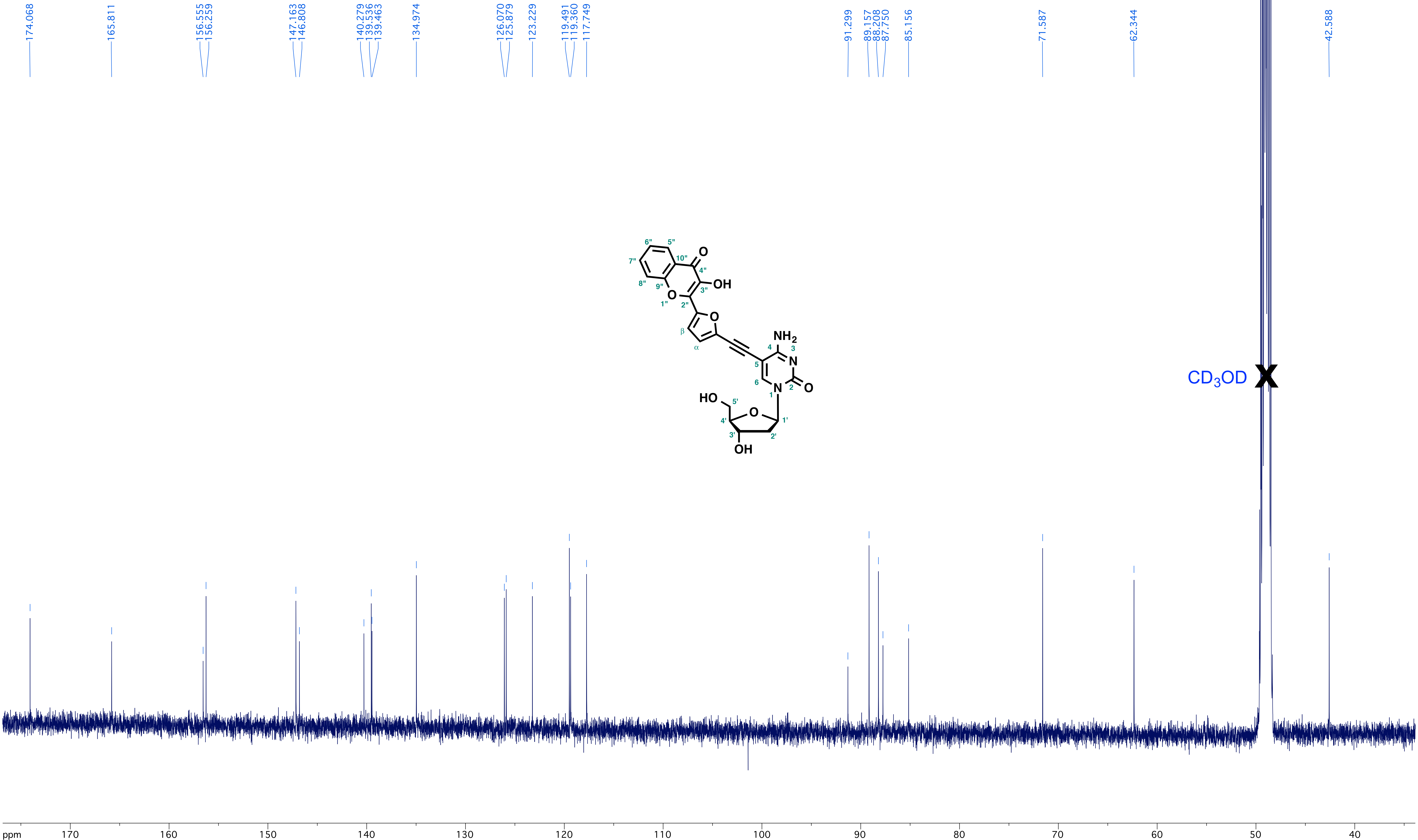
174.068
165.811
156.555
156.259
147.163
146.808
140.279
139.536
139.463
134.974
126.070
125.879
123.229
119.491
119.360
117.749
91.299
89.157
88.208
87.750
85.156
71.587
62.344
42.588
CD3OD
X
NH2
OH
HO
OH
ppm
170
160
150
140
130
120
110
100
90
80
70
60
50
40

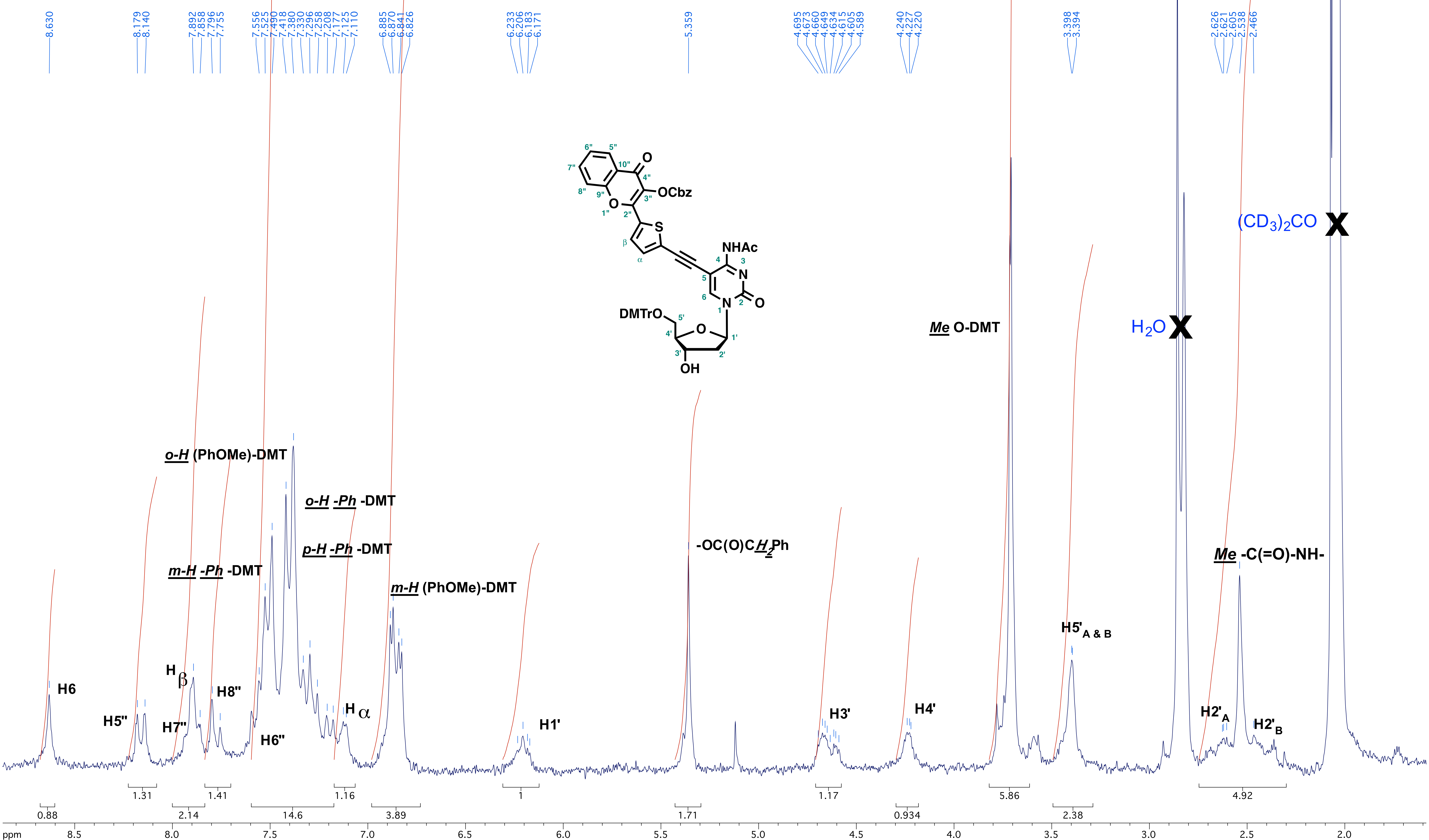

8.630
8.179
8.140
7.892
7.858
7.796
7.755
7.556
7.525
7.490
7.418
7.380
7.330
7.296
7.258
7.208
7.177
7.125
7.110
6.885
6.870
6.841
6.826
6.233
6.206
6.183
6.171
5.359
4.695
4.673
4.660
4.649
4.634
4.615
4.605
4.589
4.240
4.227
4.220
3.398
3.394
2.626
2.621
2.605
2.538
2.466
O
OCbz
S
NHAc
N
O
DMTrO
OH
Me O-DMT
H_2O X
$(CD_3)_2CO$ X
o-H (PhOMe)-DMT
o-H -Ph -DMT
p-H -Ph -DMT
m-H -Ph -DMT
m-H (PhOMe)-DMT
-OC(O)CH2Ph
Me -C(=O)-NH-
H5'A & B
H6
H5"
Hβ
H7"
H8"
H6"
Hα
H1'
H3'
H4'
H2'A
H2'B
1.31
1.41
1.16
1
1.17
5.86
4.92
0.88
2.14
14.6
3.89
1.71
0.934
2.38
ppm
8.5
8.0
7.5
7.0
6.5
6.0
5.5
5.0
4.5
4.0
3.5
3.0
2.5
2.0

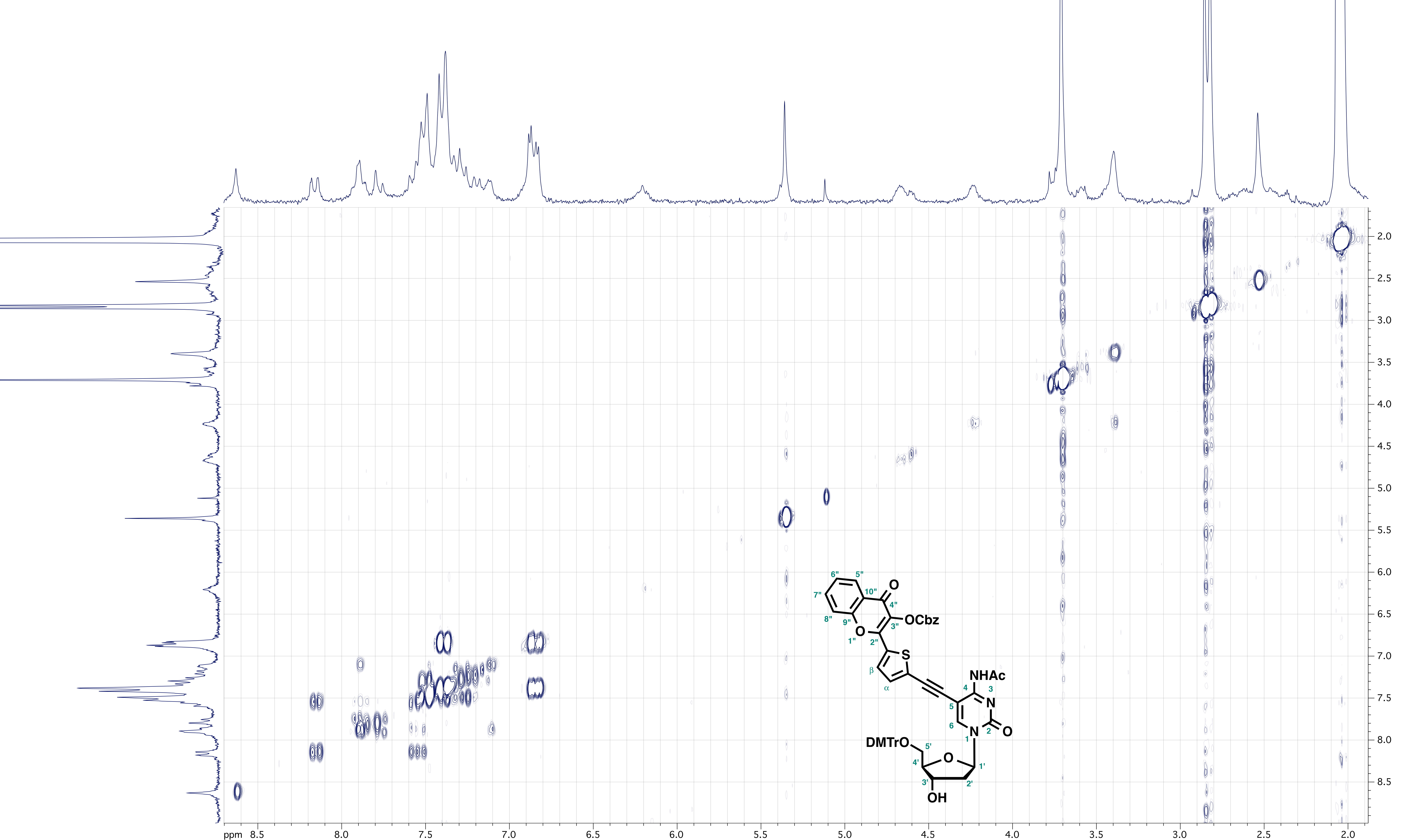
ppm
8.5
8.0
7.5
7.0
6.5
6.0
5.5
5.0
4.5
4.0
3.5
3.0
2.5
2.0
OCbz
NHAc
DMTrO
OH
S
O
N
α
β

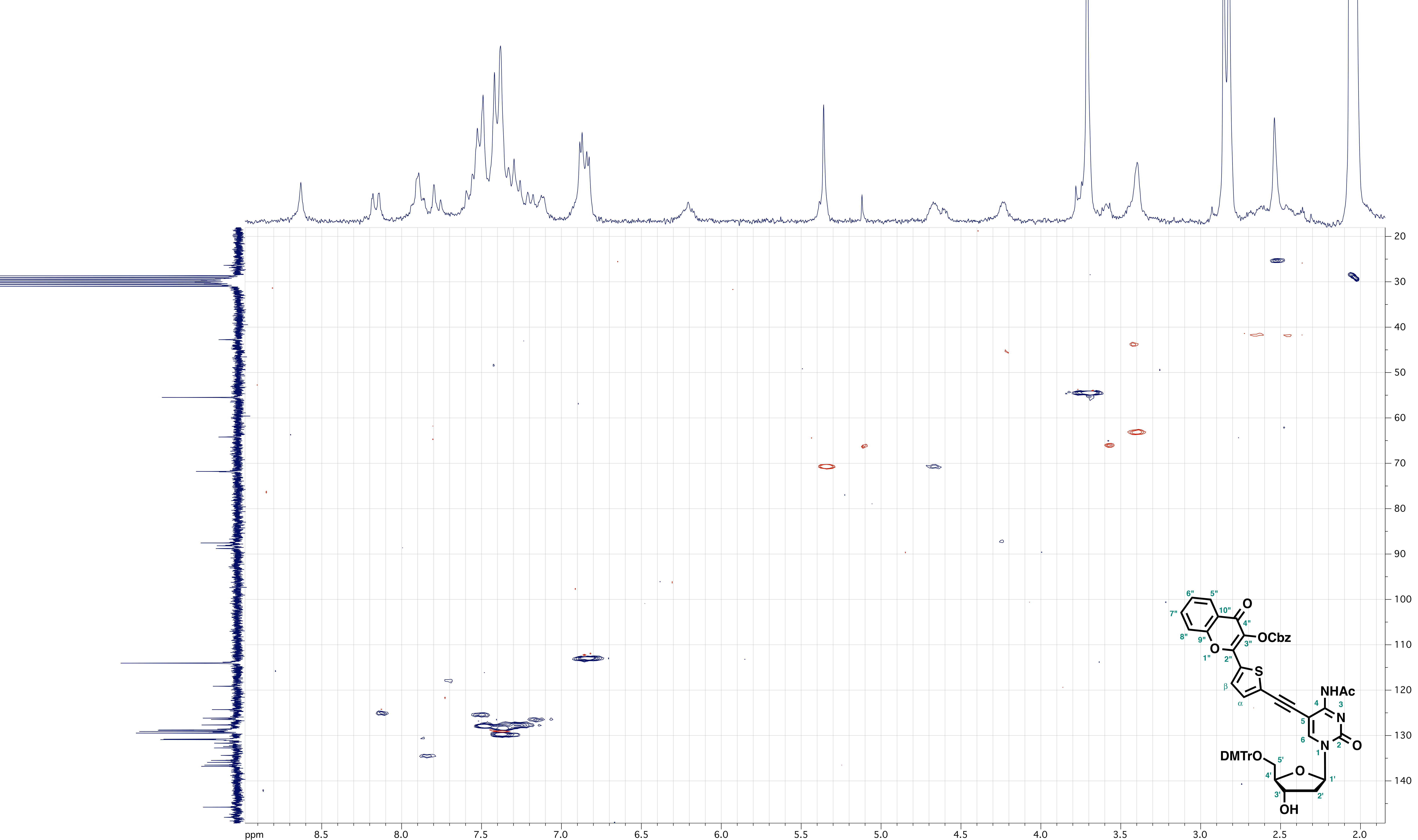

ppm
8.5
8.0
7.5
7.0
6.5
6.0
5.5
5.0
4.5
4.0
3.5
3.0
2.5
2.0
20
30
40
50
60
70
80
90
100
110
120
130
140
O
OCbz
S
NHAc
N
O
N
DMTrO
O
OH

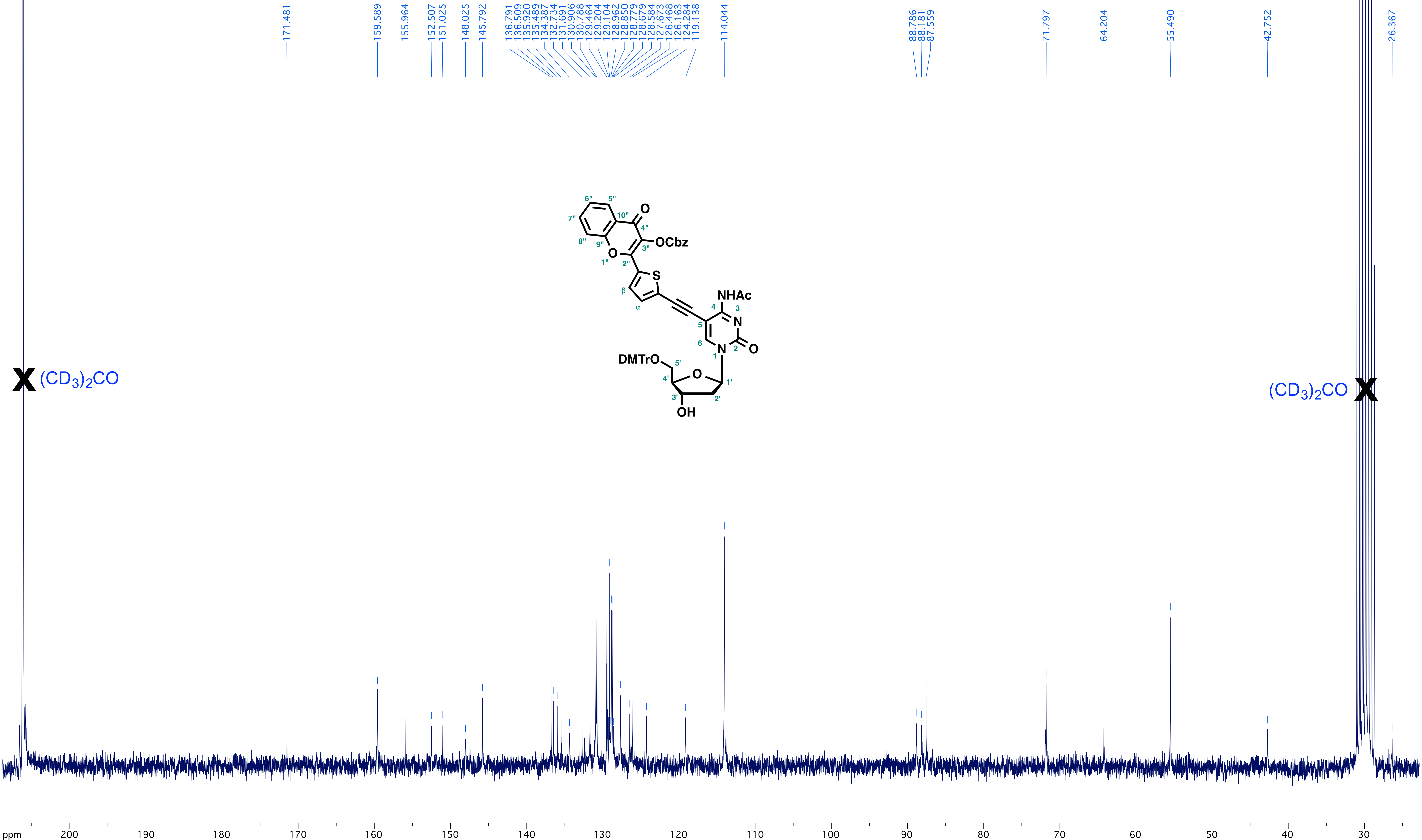
171.481
159.589
155.964
152.507
151.025
148.025
145.792
136.791
136.509
135.920
135.489
134.387
132.734
131.691
130.906
130.788
129.464
129.204
129.104
128.962
128.850
128.779
128.679
128.584
127.673
126.468
126.163
124.284
119.138
114.044
88.786
88.181
87.559
71.797
64.204
55.490
42.752
26.367
(CD3)2CO
(CD3)2CO
OCbz
NHAc
DMTrO
OH
ppm
200
190
180
170
160
150
140
130
120
110
100
90
80
70
60
50
40
30

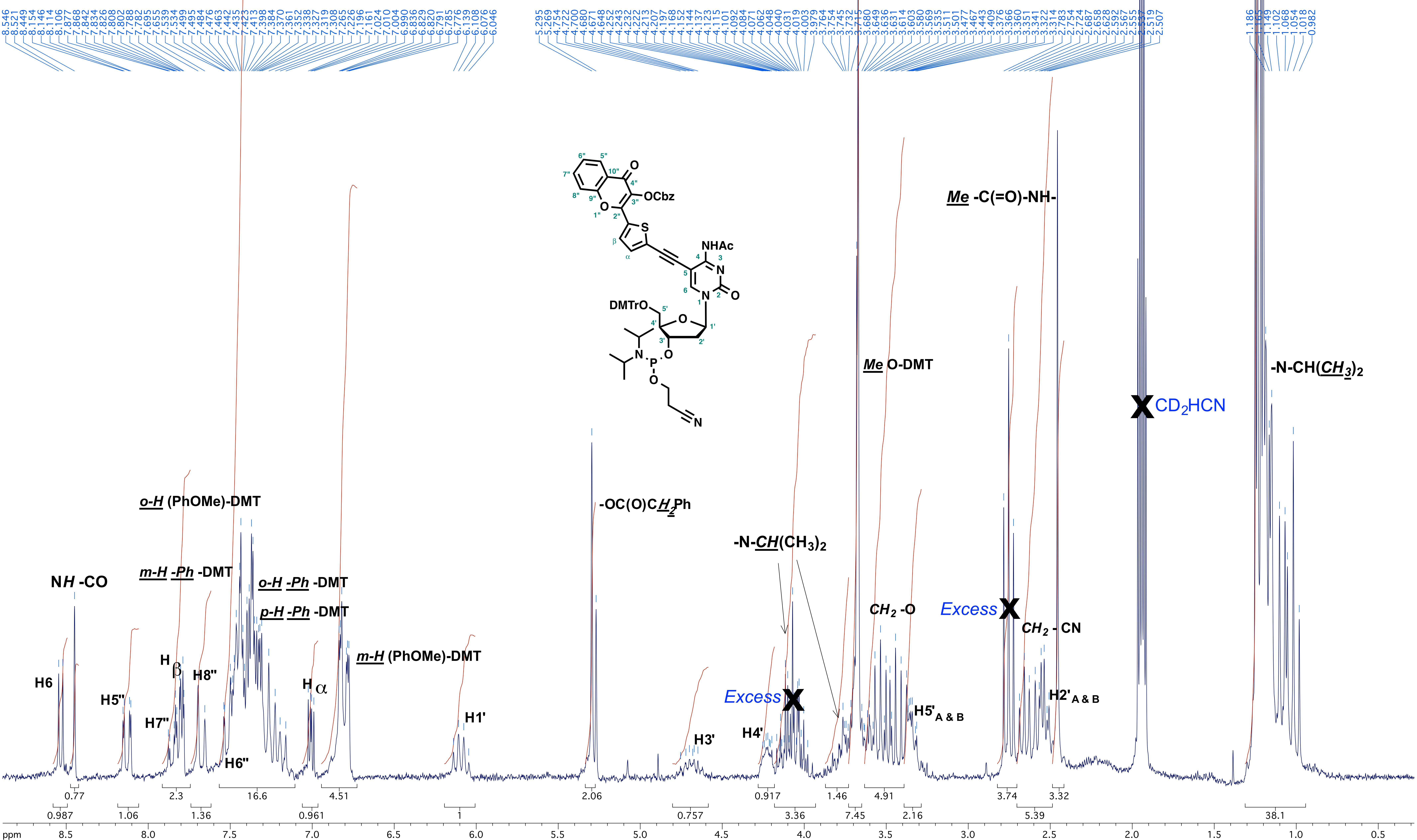

O
OCbz
S
NHAc
N
N
O
DMTrO
O
N
P
O
O
N
NH -CO
H6
H5''
H7''
H β
H8''
H6''
o-H (PhOMe)-DMT
m-H -Ph -DMT
o-H -Ph -DMT
p-H -Ph -DMT
m-H (PhOMe)-DMT
H α
H1'
-OC(O)CH2Ph
H3'
H4'
-N-CH(CH3)2
Excess
Me O-DMT
CH2 -O
H5'A & B
Excess
CH2 - CN
H2'A & B
Me -C(=O)-NH-
CD2HCN
-N-CH(CH3)2
0.987
0.77
1.06
2.3
1.36
16.6
0.961
4.51
1
2.06
0.757
0.917
3.36
1.46
7.45
4.91
2.16
3.74
5.39
3.32
38.1
ppm
8.5
8.0
7.5
7.0
6.5
6.0
5.5
5.0
4.5
4.0
3.5
3.0
2.5
2.0
1.5
1.0
0.5

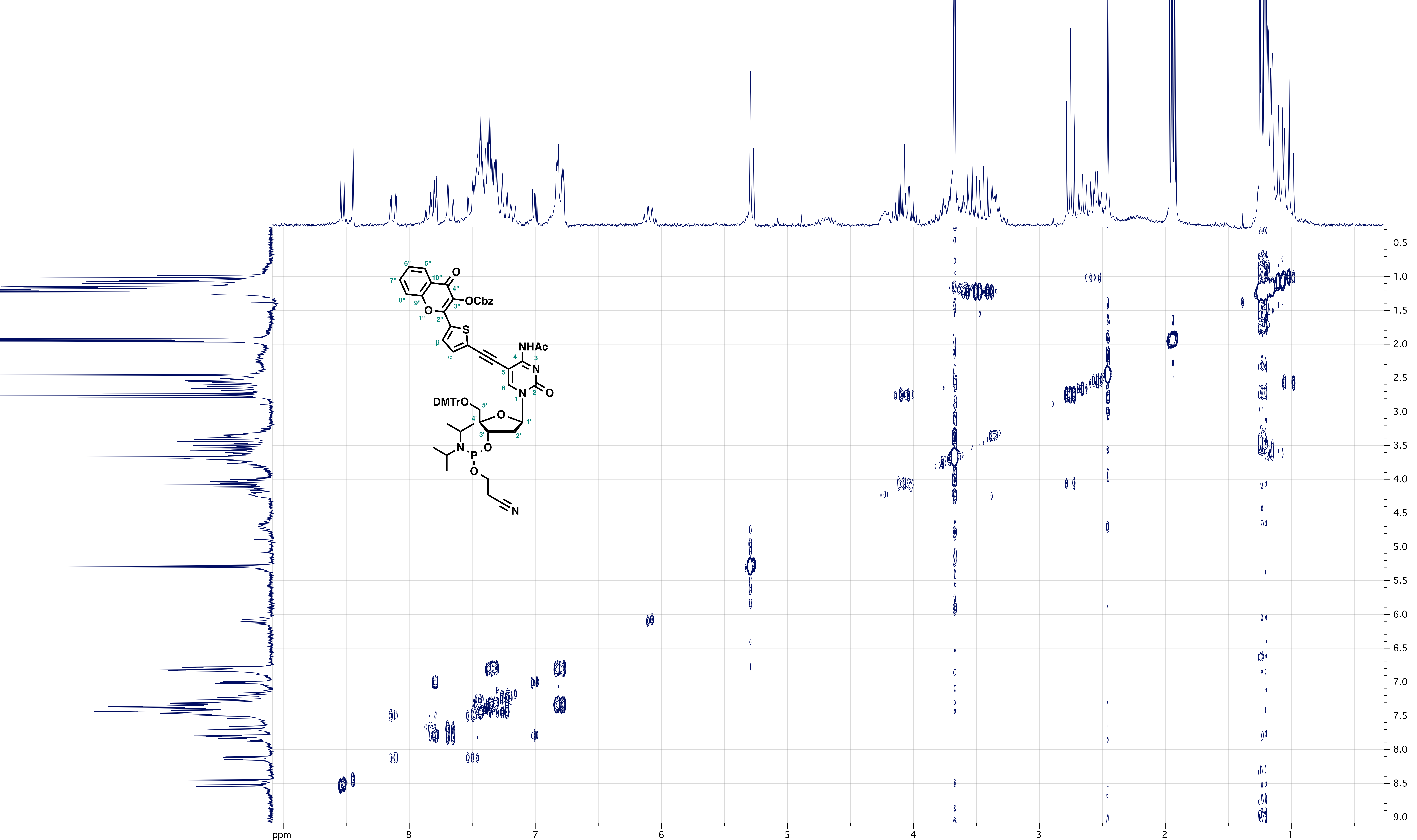
DMTrO
NHAc
OCbz
ppm

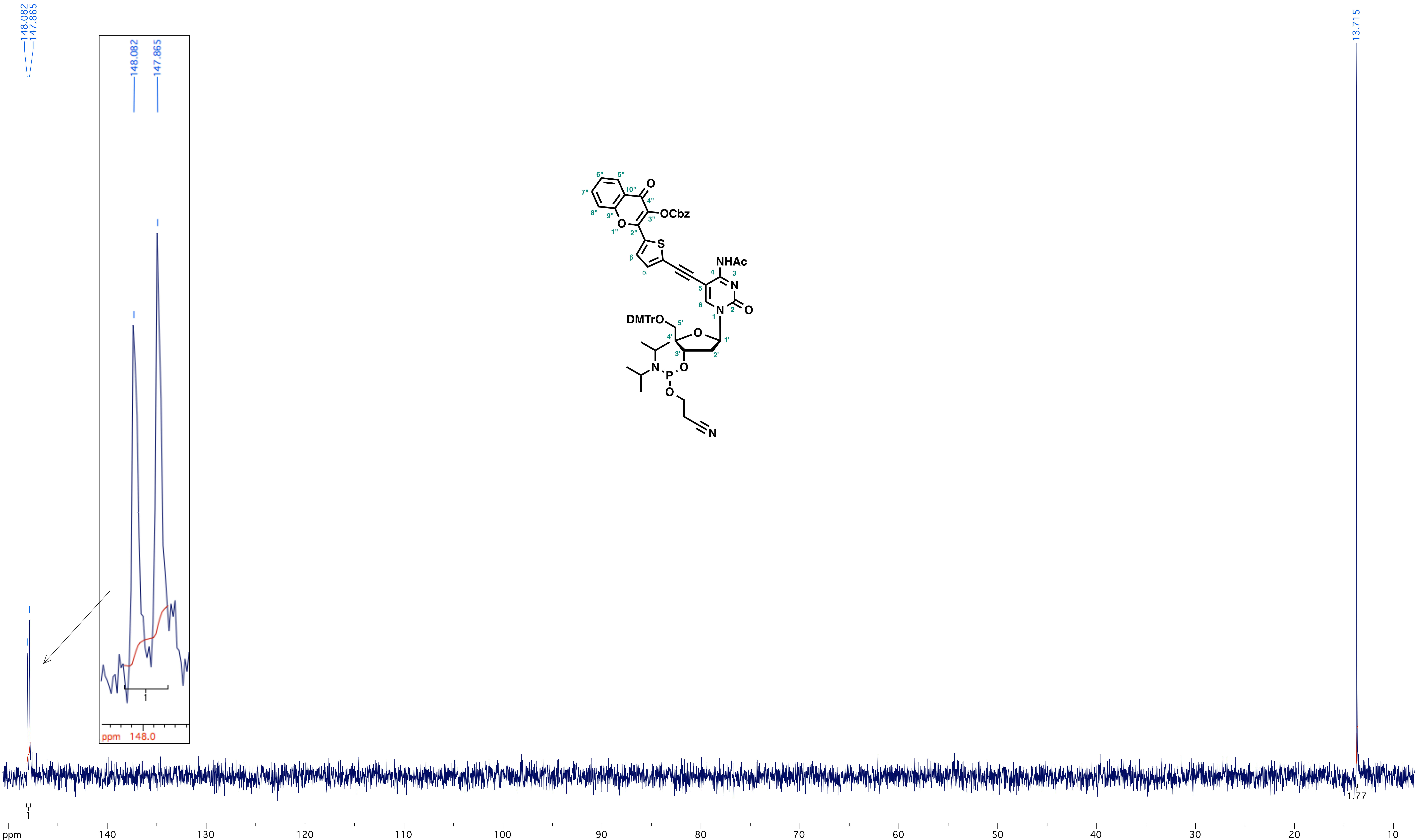
148.082
147.865
13.715
ppm 148.0
1
1.77
ppm
140
130
120
110
100
90
80
70
60
50
40
30
20
10
OCbz
NHAc
DMTrO
O
S
N
P
β
α
